# Design, Synthesis and Structure-Activity Relationship Studies of Nicotinamide Derivatives as Potent Antifungal Agents by Disrupting Cell Wall

**DOI:** 10.3390/molecules28031135

**Published:** 2023-01-23

**Authors:** Tingjunhong Ni, Fei Xie, Liping Li, Yumeng Hao, Xiaochen Chi, Lan Yan, Dazhi Zhang, Yuanying Jiang, Quanzhen Lv

**Affiliations:** 1 Department of Pharmacy, Shanghai Tenth People’s Hospital, School of Medicine, Tongji University, No. 1239 Siping Road, Shanghai 200092, China; 2 School of Pharmacy, Naval Medical University, No. 325 Guohe Road, Shanghai 200433, China; 3 School of Chinese Materia Medica, Shenyang Pharmaceutical University, Shenyang 110016, China

**Keywords:** antifungal, structure-activity relationship, nicotinamide, hit-to-lead, synthesis

## Abstract

Fungal infections pose a serious challenge to human health due to the limited paucity of antifungal treatments. Starting as a hit compound screened from our compound library, a series of nicotinamide derivatives have been successfully synthesized via a facile one-step coupling reaction of aromatic carboxylic acid and amine. The synthesized compounds were evaluated for their antifungal activity against *Candida albicans* SC5314. Among the 37 nicotinamide derivatives screened, compound **16g** was found to be the most active against *C. albicans* SC5314, with an MIC value of 0.25 μg/mL and without significant cytotoxicity. The rudimentary structure-activity relationships study revealed that the position of the amino and isopropyl groups of **16g** was critical for its antifungal activity. In particular, compound **16g** showed potent activity against six fluconazole-resistant *C. albicans* strains with MIC values ranging from 0.125–1 μg/mL and showed moderate activity against the other seven species of *Candida*, three strains of *Cryptococcus neoformans,* and three strains of *Trichophyton*. Furthermore, compound **16g** showed fungicidal, anti-hyphal, and anti-biofilm activities *in vitro*, which were related to its ability to disrupt the cell wall of *C. albicans*. Taken together, **16g** is a promising compound that is fungal-specific by targeting the cell wall and could be used as a lead compound for further investigation.

## 1. Introduction

Invasive fungal infections (IFIs) are increasingly threatening the lives of immunocompromised patients (e.g., following organ transplantation, patients with HIV/AIDS, immunosuppression, or receiving chemotherapy for cancer) [1]. It was estimated that more than 300 million people suffered from serious fungal-related diseases, and over 1.6 million people were killed by IFIs annually [2]. Notably, the mortality rate of IFIs is frequently greater than 50%. Approximately 90% of mycotic deaths are caused by three major fungal species: *Candida*, *Cryptococcus*, and *Aspergillus* [3,4]. To date, the classes of available antifungal drugs for the treatment of invasive fungal infections are still limited to azoles, polyenes, flucytosine, echinocandins, and a triterpenoid. Moreover, the clinical application of these drugs is not only limited by their drawbacks but also by drug resistance, which is a commonly occurring treatment complication. Therefore, new antifungal drugs are urgently needed.

**Figure 1:**
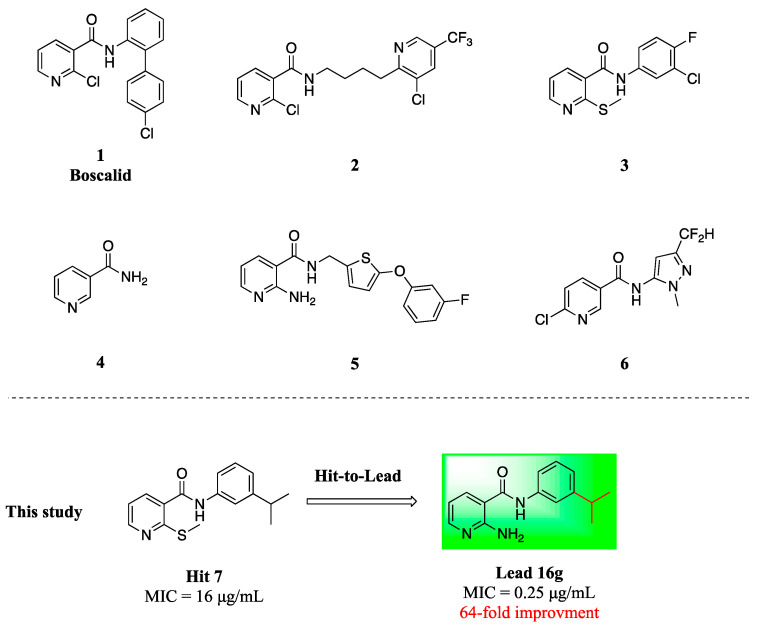
The structures of nicotinamide derivatives.

As an important class of heterocyclic derivatives, nicotinamide analogues play an important role in the development of antifungals [5,6,7,8,9,10,11]. Despite the development and commercialization of several nicotinamide analogues, most have been studied as pesticides (Figure 1). An example of such a fungicide is boscalid (**1**), which was discovered by BASF for the control of *Alternaria* late blight of pistachio [5,6]. In 2008, Queron et al. reported a 4-(3-chloro-5-(trifluoromethyl)pyridin-2-yl)butyl nicotinamide (**2**) possessing good fungicidal activities against *Alternaria alternate* [7]. In 2010, Nakamoto et al. described a nicotinamide analogue **5** possessing excellent antifungal activities against *C. albicans*, *C. neoformans,* and *A. fumigatus* [8]. In 2014, Ye et al. described that the compound *N*-(3-chloro-4-fluorophenyl)-2-(methylthio)nicotinamide (**3**) displayed moderate antifungal activity against two phytopathogenic fungi, *Rhizoctonia solani* and *Sclerotinia sclerotiorum* [9]. In 2019, our team reported that nicotinamide (**4**, Vitamin B3) showed moderate antifungal activity against *C. albicans*, including fluconazole-resistant isolates [10]. More recently, Wang and his co-workers also reported several nicotinamide derivatives (**6**) exhibiting good fungicidal activities after modification of the boscalid structure [11].

Through measuring the MIC of each compound in our in-house library, we discovered several new scaffolds with antifungal activities against *C. albicans* [12]. In this study, we focused on another moderately active antifungal hit compound, **7**, which showed an MIC value of 16 μg/mL against *C. albicans*. Although the structure of compound **7** is very similar to that of compound **3** in Figure 1, compound **3** was demonstrated to be inactive against *C. albicans* (MIC > 64 μg/mL), which suggested a possible structure-activity relationship (SAR) divergence between activity against pathogenic fungi and phytopathogenic fungi. Herein, based on compound **7**, the hit-to-lead optimization was preliminarily completed, and a series of 37 nicotinamide derivatives were designed, synthesized, and screened for their antifungal activity, which resulted in the identification of a potent and broad-spectrum lead nicotinamide derivative, namely 2-amino-*N-*(3-isopropylphenyl)nicotinamide (**16g**). Meanwhile, we assessed the cytotoxicity, anti-hyphae, and anti-biofilm activities of lead compound **16g** against *Candida albicans* in vitro. The ADMET properties of **16g** were also predicted by the SwissADME online tool to evaluate its theoretical druggability [13]. Our results indicated that the lead compound **16g** warrants further in-depth investigation as a promising antifungal agent.

## 2. Results and Discussion

### 2.1. Screening and Hit Identification

More than 30,000 compounds were screened for their antifungal activities against three of the most common pathogenic fungi *C. albicans*, *C. neoformans,* and *A. fumigatus*. After filtering out pan-assay interference compounds (PAINS, by SwissADME) and cytotoxic compounds, several hits were obtained [12,14]. Among them, compound **7** attracted our attention, which possessed a simple structure and showed moderate antifungal activity against *C. albicans* with an MIC value of 16 μg/mL and without obvious cytotoxicity against human umbilical vein endothelial cells (HUVECs) with an IC_50_ value of > 64 μg/mL. Notably, neither compound **1** nor **3** (Figure 1) showed superior antifungal activities over compound **7** (Table 1). Encouraged by these results, we decided to conduct further structure-activity relationship studies on the hit compound **7**.

### 2.2. Chemistry

**Scheme 1:**
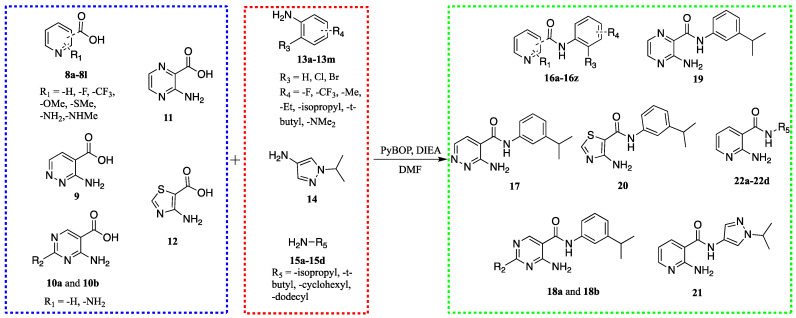
Synthesis of the target compounds. Reagents and conditions: PyBOP, DIEA, DMF, r.t, 1 h.

As depicted in Scheme 1, all compounds (**16a**-**16z**, **17**, **18a**-**18d**, **19**, **20**, **21,** and **22a**-**22d**) were synthesized facilely by a one-step coupling reaction of aromatic carboxylic acids (**8a-8l**, **9**, **10a**-**10b**, **11,** and **12**) and amines (**13a**-**13m**, **14,** and **15a**-**15d**) in the presence of PyBOP and TEA.

### 2.3. Structure-Activity Relationship

To analyze the structural determinants for antifungal activity of compound **7** against *C. albicans*, we initially replaced the *meta*-isopropyl group with *meta*-ethyl, *meta*-t-butyl, and *para*-isopropyl, giving **16a**, **16b,** and **16c**, respectively. The results are shown in Table 1. These modifications led to a decrease in potency. Furthermore, we replaced -SMe with -Ome, -CF_3_, -NHMe, -NH_2_, and -H, giving **16d**, **16e**, **16f**, **16g,** and **16h**, respectively. All these modifications were found unfavorable for antifungal activity with the exception of compound **16g,** which showed excellent antifungal activity against *C. albicans* SC5314. The MIC value of **16g** for *C. albicans* was 0.25 μg/mL, which was comparable to FLC.

Due to the high potency exhibited by **16g,** we used it as a new starting point for further structural optimization. The results are shown in Table 2. Initially, modifications focused on the *meta*-isopropyl aniline moiety. Moving the *meta*-isopropyl group to the *ortho*- or *para*-position of the aniline moiety gave the compounds **16i** and **16j**, respectively. *Para*-substitution (**16j**) was relatively well-tolerated with a 2-fold decrease in potency (MIC = 0.5 μg/mL), whereas *ortho*-substitution (**16i**) resulted in a complete loss of activity (MIC > 64 μg/mL). In addition, the replacement of the isopropyl group with fluorine and trifluoromethyl, methyl, ethyl, tert-butyl, and dimethylamino groups to give compounds **16k**-**16r** resulted in varying degrees of potency reduction. Moreover, the introduction of chlorine and bromine into aniline, giving **16s** and **16t**, respectively, proved unfavorable with a 32-fold and 16-fold reduction in activity. Subsequently, we investigated the effect of the 2-amino pyridine moiety of **16g** by varying the position of the amino group and nitrogen atom while fixing *meta*-isopropyl aniline to give compounds **16u**-**16z**. Unfortunately, these modifications appeared to be unhelpful. Furthermore, the incorporation of a second nitrogen atom into the pyridine ring gave the diazine analogue **17**, the pyrimidine analogues **18a** and **18b**, and the pyrazine analogue **19**, which also yielded disappointing results, despite **19** exhibiting moderate antifungal activity with an MIC value of 4 μg/mL. Replacement of the pyridine ring with it’s bioisosteric thiazole ring, giving **20**, also demonstrated an unfavorable result. In a final study, we replaced the phenyl of the aniline moiety with pyrazole (**21**), isopropyl (**22a**), tert-butyl (**22b**), cyclohexyl (**22c**), and dodecyl (**22d**), all of which were found to be detrimental to antifungal activity.

Overall, the results discussed above shed light on 2-aminopyridine and *meta*-isopropyl as the essential moieties for the antifungal activity of this scaffold. Therefore, the most active compound, **16g,** was selected for further investigation.

### 2.4. Compounds ***16g*** and ***16j*** Exhibit Low Toxicity to Mammalian Cells

**Figure 2:**
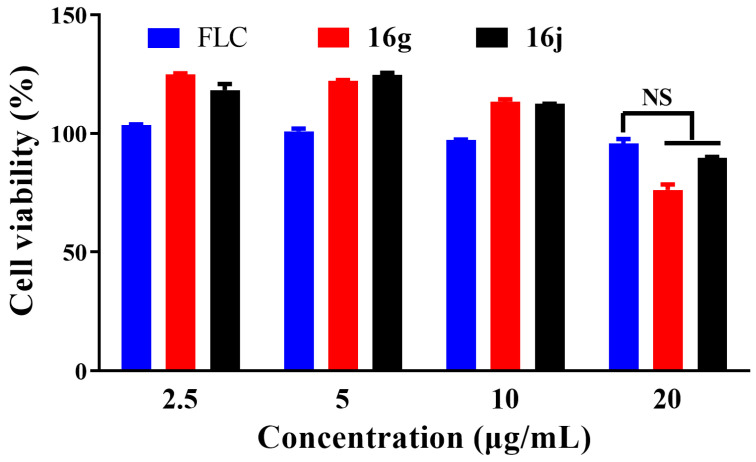
The in vitro toxicity evaluation of **16g**, **16j** and FLC. The cytotoxic effects of compounds **16g** and **16j**, compared to that of FLC, on HUVECs viability was assessed by the CCK-8 test following a 2 h treatment. FLC: fluconazole.

To confirm the antifungal effects were not due to the cytotoxicities of compounds **16g** and **16j**, HUVEC cells were treated with various concentrations (2.5, 5, 10, and 20 μg/mL) of FLC, **16g,** and **16j** for 24 h. Cell viability was measured by the CCK8 assay. As shown in Figure 2, we found that FLC, **16g,** and **16j** did not cause significant cytotoxicity below 20 μg/mL, and no significant difference was observed between the two compounds and fluconazole.

### 2.5. Compound ***16g*** Exhibits Broad-Spectrum Antifungal Activity

To investigate the antifungal effect of this chemotype, compound **16g** was selected to screen for in vitro activity against two strains of fluconazole-sensitive *C. albicans* and six strains of fluconazole-resistant *C. albicans* (Table 3). The antifungal activity of **16g** against the two fluconazole-sensitive strains was comparable to that of fluconazole, with MIC values ranging from 0.125–0.5 μg/mL. In contrast, the activity of **16g** against the six fluconazole-resistant strains was significantly superior to fluconazole, with MIC values ranging from 0.125–1 μg/mL. In addition, we further evaluated the antifungal spectrum of **16g** (Table 4). Our results demonstrated that the compound **16g** had moderate activities against *Candida*, *Cryptococcus,* and *Trichophyton*, which were equivalent to FLC and ineffective against *A. fumigatus*.

### 2.6. Fungicidal Activity of ***16g*** against C. albicans

**Figure 3:**
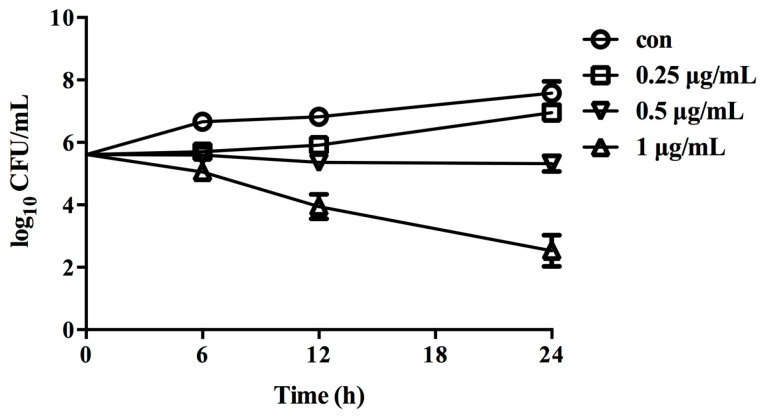
Time-killed curves for **16g** against the *C. albicans*. The concentrations of **16g** were adjusted to 0.25 μg/mL (1 × MIC), 0.5 μg/mL (2 × MIC), 1 μg/mL (4 × MIC) in RPMI 1640 medium. con: control.

Time-kill curves showed that **16g** at concentrations of 0.25 μg/mL and 0.5 μg/mL had a slight inhibitory effect on the growth of *C. albicans* (Figure 3). However, 1 μg/mL of **16g** killed *C. albicans* after 24 h of treatment, which resulted in a log10 CFU/mL decrease from 5.5 to 2, approximately. Our results indicated that higher concentrations of **16g** showed fungicidal activities.

### 2.7. Compound ***16g*** Inhibits the Hyphae Formation of C. albicans

**Figure 4:**
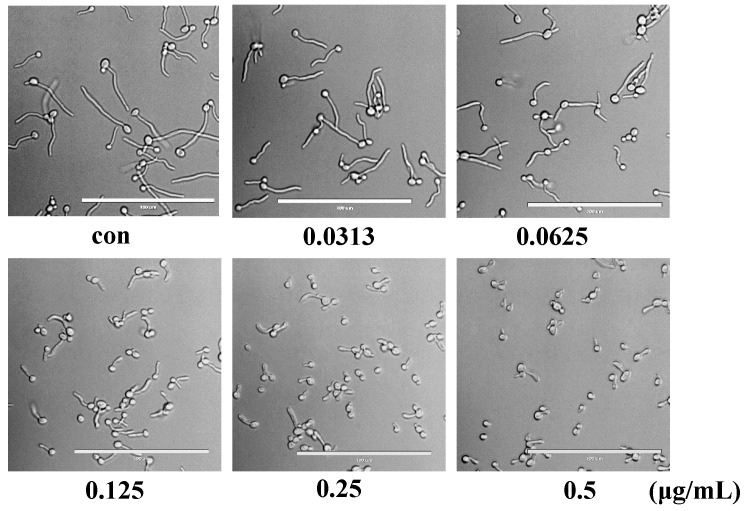
Anti-hyphae effects of different concentrations of compound **16g**. The inhibition of **16g** on the hypha formation of *C. albicans*. *C. albicans* SC5314 were incubated at 37 °C for 3 h in RPMI 1640 medium. The concentrations of **16g** were ranged from 0.0313–0.5 μg/mL. con: control.

The morphological transition from yeast to hypha is the major contributor to the in vivo pathogenicity of *C. albicans* [15,16]. Therefore, we further investigated the activity of compound **16g** against the yeast-to-hypha transition of *C. albicans*. As shown in Figure 4, 0.125 μg/mL or higher concentrations of compound **16g** exhibited potent activity against *C. albicans* hypha formation, which showed fewer hyphae and more pseudohyphal cells.

### 2.8. Compound ***16g*** Inhibits the Biofilm Formation in C. albicans

**Figure 5:**
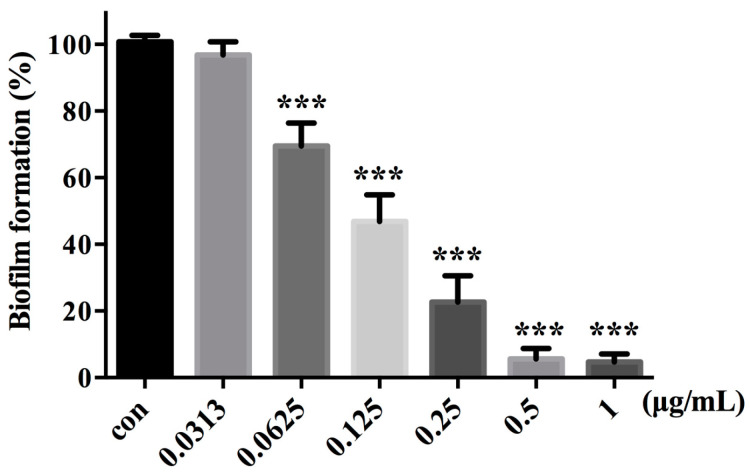
**16g** inhibits *C. albicans* biofilm formation in vitro. Biofilm formation was evaluated by XTT reduction assay using *C. albicans* SC5314. The results were repeated for two times. *** *p* < 0.001, (*t* test).

Biofilm formation is an important factor in the pathogenesis of *C. albicans*, which leads to high resistance to a wide range of antifungals [17]. In this study, we examined the effect of **16g** on the biofilm formation of *C. albicans*. XTT reduction assays revealed that **16g** showed an inhibitory effect on the biofilm formation in a dose-dependent manner (Figure 5). More specifically, 0.0625 μg/mL of **16g** inhibited the biofilm formation by approximately 30%, and the inhibitory activity on the biofilm was enhanced as the concentrations of **16g** increased. The addition of 0.125 μg/mL of **16g** inhibited biofilm formation by 50%, while over 90% of the biofilms were inhibited in the presence of 0.5 μg/mL of **16g**.

### 2.9. ***16g*** Treatment Significantly Disrupted the Cell Wall Morphology of C. albicans

**Figure 6:**
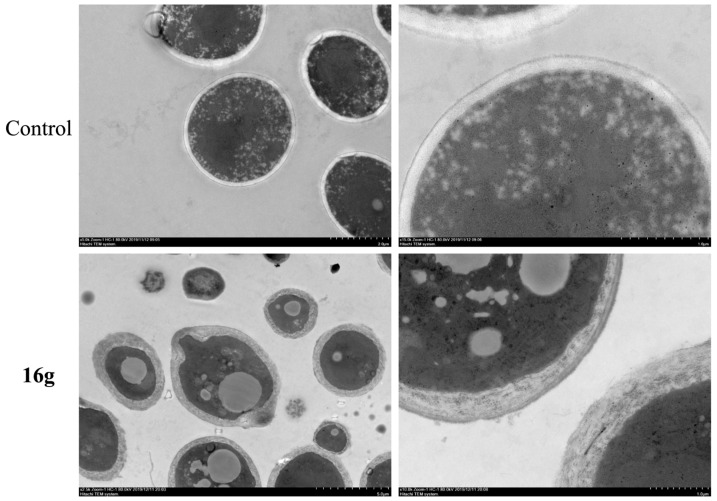
Cell wall damage caused by compound **16g** in *C. albicans*. *C. albicans* SC5314 was treated with 1 μg/mL of **16g** for 16 h. The structural changes of cell wall were observed by transmission electron microscope. con: control.

To further explore the antifungal mechanisms of nicotinamide derivatives, we investigated structural changes in cells treated with 1 μg/mL of **16g**. As shown in Figure 6, the cell wall of **16g**-treated cells displays broken edges and a thickened distance between the cell wall and cell membrane. Meanwhile, the gaps between the cell wall and cell membrane were filled with cytosolic fluid, which suggested the weakened protective effect of the cell wall was disrupted by **16g**. Our results indicated that **16g** could inhibit the growth of *C. albicans* by disrupting the cell walls.

### 2.10. ADMET Prediction

**Figure 7:**
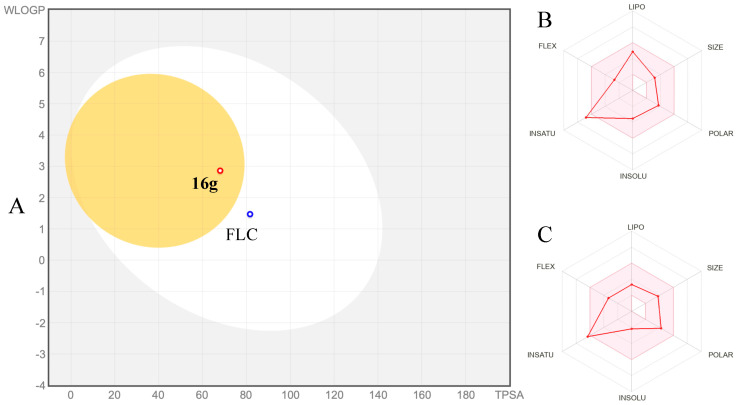
(**A**)The BOILED-Egg model prediction of **16g** and FLC; (**B**) Bioavailability radar for **16g**; (**C**) Bioavailability radar for FLC; FLC: fluconazole.

In silico ADMET prediction of compounds **16g** and FLC was performed using the free SwissADME online tool [13]. The Brain Or IntestinaL EstimateD permeation (BOILED-Egg) method is a graphical model that works by calculating the polarity and lipophilicity of small molecules. According to Figure 7A, compound **16g** was located in the yellow circle while FLC was in the white circle, representing that both compounds were highly absorbed through the gastrointestinal tract, but compound **16g** penetrates the blood-brain barrier more readily than FLC. In addition, the compound **16g** with the red spot is not a substrate for P-glycoprotein, which perhaps facilitated overcoming efflux pump-mediated resistance mechanisms in pathogenic fungi. Furthermore, as shown in the bioavailability radar (Figure 7B,C), the physicochemical characteristics, lipophilicity, solubility, pharmacokinetics, and drug-likeness properties of compound **16g** and FLC were all located in the pink area (optimal range), indicating that compound **16g**, such as FLC, is a good lead compound.

## 3. Materials and Methods

### 3.1. General Procedure for the Synthesis of Target Compounds

To a solution of aromatic carboxylic acid (**8a-8l**, **9**, **10a**, **10b**, **11,** and **12**, 1 mmol), DIEA (2 mmol) and PyBOP (1.1 mmol) in DMF (5 mL) were added to various anilines (**13a**-**13m** and **14**) or amines (**15a**-**15d**, 1 mmol) at room temperature. The mixture was stirred at this temperature for 2 h. The reaction was monitored by TLC. After the reaction was finished, the mixture was poured into water, and then the mixture was extracted with EtOAc (2 × 10 mL). After washing with brine (2 × 10 mL) and drying over anhydrous Na_2_SO_4_, the organic phase was evaporated in a vacuum. The crude product was purified by silica gel column chromatography using EtOAc/PE (1:1) as the eluent to give target compounds (**16a**-**16z**, **17**, **18a**, **18b**, **19**, **20**, **21,** and **22a**-**22d**). The NMR spectra can be found in the Supplementary Materials.

**16a**: 238 mg, yield: 87%. White solid. ^1^H NMR (300 MHz, CDCl_3_-*d*_1_) *δ* 8.52 (d, *J* = 4.0 Hz, 1H), 8.24 (s, 1H), 7.88 (d, *J* = 7.5 Hz, 1H), 7.52 (s, 1H), 7.45 (d, *J* = 8.1 Hz, 1H), 7.28 (dd, *J* = 8.8, 6.8 Hz, 2H), 7.12 − 6.97 (m, 2H), 2.74 − 2.55 (m, 5H), 1.25 (t, *J* = 7.6 Hz, 3H). ^13^C NMR (151 MHz, DMSO-*d*_6_) *δ* 165.33, 157.91, 150.58, 144.78, 139.38, 135.79, 131.03, 129.09, 123.86, 119.65, 119.22, 117.76, 28.75, 16.00, 13.46.

**16b**: 273 mg, yield: 90%. White solid. ^1^H NMR (300 MHz, CDCl_3_-*d*_1_) *δ* 8.55 (dd, *J* = 4.8, 1.5 Hz, 1H), 8.17 (s, 1H), 7.92 (d, *J* = 7.3 Hz, 1H), 7.63 (s, 1H), 7.53 (d, *J* = 7.8 Hz, 1H), 7.31 (d, *J* = 7.9 Hz, 1H), 7.22 (d, *J* = 7.9 Hz, 1H), 7.10 (dd, *J* = 7.6, 4.9 Hz, 1H), 2.63 (s, 3H), 1.35 (s, 9H). ^13^C NMR (151 MHz, DMSO-*d*_6_) *δ* 165.31, 157.95, 151.74, 150.57, 139.17, 135.81, 131.00, 128.82, 121.31, 119.19, 117.49, 117.27, 34.92, 31.57, 13.47.

**16c**: 254 mg, yield: 89%. White solid. ^1^H NMR (300 MHz, CDCl_3_-*d*_1_) *δ* 8.56 (d, *J* = 3.1 Hz, 1H), 8.05 (s, 1H), 7.95 (d, *J* = 7.1 Hz, 1H), 7.57 (d, *J* = 8.2 Hz, 2H), 7.25 (d, *J* = 8.2 Hz, 2H), 7.17 − 7.09 (m, 1H), 2.96 − 2.87 (m, 1H), 2.64 (s, 3H), 1.25 (d, *J* = 6.9 Hz, 6H). ^13^C NMR (75 MHz, DMSO-*d*_6_) *δ* 165.18, 157.91, 150.56, 144.50, 137.12, 135.76, 131.04, 126.90, 120.39, 119.22, 33.40, 24.42, 13.46.

**16d**: 239 mg, yield: 88%. Yellow solid. ^1^H NMR (300 MHz, DMSO-*d*_6_) *δ* 10.34 (s, 1H), 9.56 (s, 1H), 8.78 (d, *J* = 1.4 Hz, 1H), 8.20 (d, *J* = 8.5 Hz, 1H), 8.10 (dd, *J* = 8.6, 1.8 Hz, 1H), 7.65 (d, *J* = 8.8 Hz, 2H), 7.27 (t, *J* = 7.8 Hz, 1H), 6.99 (d, *J* = 7.7 Hz, 1H), 2.96 − 2.79 (m, 1H), 1.21 (d, *J* = 6.9 Hz, 7H). ^13^C NMR (151 MHz, DMSO-*d*_6_) *δ* 165.40, 159.49, 155.27, 149.34, 139.62, 134.10, 132.69, 128.99, 126.24, 123.20, 122.95, 122.34, 118.74, 118.39, 33.99, 24.35.

**16e**: 263 mg, yield: 85%. Semi-solid. ^1^H NMR (300 MHz, DMSO-*d*_6_) *δ* 10.62 (s, 1H), 8.90 − 8.78 (m, 1H), 8.20 (dd, *J* = 7.8, 0.8 Hz, 1H), 7.84 (dd, *J* = 7.8, 4.8 Hz, 1H), 7.62 − 7.42 (m, 2H), 7.27 (t, *J* = 7.8 Hz, 1H), 7.02 (d, *J* = 7.7 Hz, 1H), 2.93 − 2.79 (m, 1H), 1.19 (d, *J* = 6.9 Hz, 6H). ^13^C NMR (151 MHz, DMSO-*d*_6_) *δ* 164.24, 150.49, 149.64, 142.96, 142.74, 139.11, 137.88, 132.88, 129.25, 127.67, 122.68, 118.01, 117.68, 33.95, 24.29.

**16f**: 231 mg, yield: 86%. White solid. ^1^H NMR (300 MHz, DMSO-*d*_6_) *δ* 10.10 (s, 1H), 8.21 (dd, *J* = 4.8, 1.7 Hz, 1H), 8.02 (dd, *J* = 7.7, 1.8 Hz, 1H), 7.85 (d, *J* = 4.7 Hz, 1H), 7.58 (d, *J* = 1.7 Hz, 1H), 7.55 − 7.46 (m, 1H), 7.24 (t, *J* = 7.8 Hz, 1H), 6.97 (d, *J* = 7.7 Hz, 1H), 6.61 (dd, *J* = 7.6, 4.8 Hz, 1H), 2.97 − 2.78 (m, 4H), 1.20 (d, *J* = 6.9 Hz, 6H). ^13^C NMR (151 MHz, DMSO-*d*_6_) *δ* 167.10, 158.39, 151.77, 149.25, 139.33, 137.27, 128.88, 122.32, 119.10, 118.73, 111.25, 110.65, 33.98, 28.19, 24.33.

**16g**: 204 mg, yield: 80%. White solid. Melting point:127.3 °C. ^1^H NMR (300 MHz, CDCl_3_-*d*_1_) *δ* 8.24 − 8.23 (d, *J* = 3 Hz, 1H), 7.81 − 7.78 (d, *J* = 9 Hz, 1H), 7.71 (s, 1H), 7.44 − 7.42 (t, *J* = 6.3 Hz, 2 H), 7.36 − 7.33 (d, *J* = 9 Hz, 1 H), 7.09 − 7.07 (d, *J* = 9 Hz, 1H), 6.72−6.68 (dd, *J* = 7.7, 4.9 Hz, 1H), 6.38 (s, 2H), 3.00 − 2.91 (m, 1H), 1.31 − 1.28 (d, *J* = 9 Hz, 6H). ^13^C NMR (75 MHz, DMSO-*d*_6_) *δ* 166.93, 159.15, 151.77, 149.26, 139.34, 137.72, 128.90, 122.31, 119.09, 118.74, 111.82, 110.77, 33.97, 24.34.

**16h**: 198 mg, yield: 82%. Semi-solid. ^1^H NMR (300 MHz, DMSO-*d*_6_) *δ* 10.37 (s, 1H), 9.09 (d, *J* = 1.7 Hz, 1H), 8.75 (dd, *J* = 4.8, 1.6 Hz, 1H), 8.33 – 8.23 (m, 1H), 7.66 − 7.50 (m, 3H), 7.26 (t, *J* = 7.8 Hz, 1H), 7.00 (d, *J* = 7.7 Hz, 1H), 2.92 − 2.82 (m, 1H), 1.20 (d, *J* = 6.9 Hz, 6H). ^13^C NMR (151 MHz, DMSO-*d*_6_) *δ* 164.41, 152.51, 149.39, 149.13, 139.29, 135.85, 131.11, 129.03, 123.92, 122.57, 118.77, 118.43, 33.96, 24.33.

**16i**: 214 mg, yield: 84%. White solid. ^1^H NMR (300 MHz, DMSO-*d*_6_) *δ* 9.86 (s, 1H), 8.14 − 8.09 (m, 2H), 7.40 − 7.16 (m, 4H), 7.05 (s, 2H), 6.68 − 6.64 (m, 1H), 3.21 − 3.03 (m, 1H), 1.15 (d, *J* = 6.9 Hz, 6H). ^13^C NMR (75 MHz, DMSO-*d*_6_) *δ* 167.74, 159.50, 152.07, 145.50, 137.38, 135.23, 128.73, 127.46, 126.31, 126.10, 111.90, 109.88, 28.02, 23.63.

**16j**: 203 mg, yield: 80%. White solid. Melting point: 180.0 °C. ^1^H NMR (300 MHz, DMSO-*d*_6_) *δ* 10.07 (s, 1H), 8.11 (dd, *J* = 4.8, 1.8 Hz, 1H), 8.01 (dd, *J* = 7.7, 1.8 Hz, 1H), 7.58 (d, *J* = 8.5 Hz, 2H), 7.20 (d, *J* = 8.5 Hz, 2H), 6.95 (s, 2H), 6.66 − 6.62 (m, 1H), 2.93 − 2.75 (m, 1H), 1.18 (d, *J* = 6.9 Hz, 6H). ^13^C NMR (75 MHz, DMSO-*d*_6_) *δ* 166.89, 159.26, 151.94, 144.34, 137.52, 137.09, 126.72, 121.29, 111.83, 110.68, 33.38, 24.42.

**16k**: 187 mg, yield: 81%. White solid. ^1^H NMR (300 MHz, CDCl_3_-*d*_1_) *δ* 8.28 (s, 1H), 8.13 − 8.12 (d, *J* =3 Hz, 1H), 7.95 − 7.92 (d, *J* = 9 Hz, 1H), 7.62 − 7.59 (d, *J* = 9 Hz, 1 H), 7.40 − 7.30 (m, 2H), 6.94 − 6.88 (t, *J* = 8.7 Hz, 1H), 6.77 − 6.73 (dd, *J* = 7.5, 5.1 Hz, 3H). ^13^C NMR (151 MHz, DMSO-*d*_6_) *δ* 167.25, 163.29, 161.69, 159.24, 152.33, 141.25, 141.18, 137.74, 130.66, 130.60, 116.69, 111.83, 110.63, 110.50, 110.25, 107.77, 107.60.

**16l**: 172 mg, yield: 76%. White solid. ^1^H NMR (300 MHz, CDCl_3_-*d*_1_) *δ* 8.24 − 8.22 (d, *J* = 6 Hz, 1H), 7.79 − 7.73 (t, *J* = 9.6Hz 2H), 7.44 (s, 1H), 7.37 − 7.26 (m, 2H), 7.03 − 7.01 (d, *J* = 6 Hz, 1H), 6.71 − 6.67 (dd, *J* = 7.5, 4.9 Hz, 1H), 6.36 (s, 2H), 2.40(s, 3H). ^13^C NMR (151 MHz, DMSO-*d*_6_) *δ* 167.00, 159.27, 151.99, 139.30, 138.17, 137.59, 128.85, 124.88, 121.69, 118.33, 111.83, 110.65, 21.65.

**16m**: 179 mg, yield: 79%. White solid. ^1^H NMR (300 MHz, CDCl_3_-*d*_1_) *δ* 8.26 − 8.24 (d, *J* = 6Hz, 1H), 7.82 − 7.77 (t, *J* = 8.5 Hz, 2H), 7.58 (s, 1H), 7.32 (s, 1H), 7.27 (s, 1H), 7.21 − 7.18 (t, *J* = 7.3 Hz, 1H), 6.73 − 6.69 (dd, *J* = 7.6, 4.9 Hz, 1H), 6.40 (s, 2H), 2.35 (s, 3H). ^13^C NMR (151 MHz, DMSO-*d*_6_) *δ* 167.04, 159.48, 152.07, 137.52, 136.68, 134.39, 130.74, 127.24, 126.51, 126.45, 111.87, 109.94, 46.33, 46.31, 26.39, 26.34, 18.34.

**16n**: 163 mg, yield: 72%. White solid. ^1^H NMR (300 MHz, CDCl_3_-*d*_1_) *δ* 8.23 − 8.21 (dd, *J* = 4.8, 1.4 Hz, 1H), 7.80 − 7.77 (m, 2H), 7.47 − 7.44 (d, *J* = 9 Hz, 2H), 7.22 − 7.19 (d, *J* = 8.2 Hz, 2H), 6.71 − 6.67 (dd, *J* = 7.7, 4.9 Hz, 1H), 6.36 (s, 2H), 2.37 (s, 3H). ^13^C NMR (151 MHz, DMSO) *δ* 166.88, 159.27, 151.93, 137.53, 136.83, 133.17, 129.41, 121.19, 111.83, 110.67, 20.95.

**16o**: 237 mg, yield: 84%. White solid. ^1^H NMR (300 MHz, CDCl_3_-*d*_1_) *δ* 8.23 − 8.21 (d, *J* = 6 Hz, 1H), 8.05 (s, 1H), 7.94 (s, 1H), 7.87 − 7.79 (dd, *J* = 16.7, 7.7 Hz, 2H), 7.56 − 7.44 (m, 2H), 6.75 − 6.71 (dd, *J* = 7.5, 4.9 Hz, 1H), 6.53 (s, 2H). ^13^C NMR (151 MHz, DMSO-*d*_6_) *δ* 167.39, 159.28, 152.45, 140.26, 137.81, 130.24, 124.48, 120.36, 117.05, 111.82, 110.03.

**16p**: 198 mg, yield: 82%. White solid. Melting point: 164.7 °C. ^1^H NMR (300 MHz, CDCl_3_-*d*_1_) *δ* 8.20 − 8.18 (dd, *J* = 4.9, 1.5 Hz, 1H), 7.85 − 7.82 (d, *J* = 9Hz, 2H), 7.45 − 7.32 (m, 3H), 7.07 − 7.04 (d, *J* = 9 Hz, 1H), 6.73 − 6.69 (dd, *J* = 7.7, 5.0 Hz, 1H), 6.52 (s, 2H), 2.74 − 2.66 (q, *J* = 7.6 Hz, 2H),1.31 − 1.26 (t, *J* = 7.6 Hz, 3H). ^13^C NMR (151 MHz, DMSO-*d*_6_) *δ* 167.00, 159.27, 151.99, 144.56, 139.37, 137.59, 128.91, 123.70, 120.53, 118.59, 111.82, 110.65, 28.74, 15.99.

**16q**: 221 mg, yield: 82%. White solid. Melting point: 117.5 °C. ^1^H NMR (300 MHz, CDCl_3_-*d*_1_) *δ* 8.23 − 8.21 (d, *J* = 6 Hz, 1H), 7.81 − 7.79 (d, *J* = 6 Hz, 2H), 7.52 − 7.46 (m, 2H), 7.37 − 7.32 (t, *J* = 15 Hz, 1H), 7.25 − 7.22 (d, *J* = 9 Hz, 1H), 6.71 − 6.66 (m, 1H), 6.36 (s, 2H), 1.36(s, 9H). ^13^C NMR (151 MHz, DMSO-*d*_6_) *δ* 167.00, 159.25, 151.97, 151.56, 139.11, 137.59, 128.63, 121.13, 118.38, 118.19, 111.81, 110.71, 34.91, 31.59.

**16r**: 201 mg, yield: 78%. White solid. Melting point:168.2 °C. ^1^H NMR (300 MHz, CDCl_3_-*d*_1_) *δ* 8.23 − 8.21 (d, *J* = 6 Hz, 1H), 7.80 − 7.77 (d, *J* = 9 Hz, 1H), 7.69 (s, 1H), 7.27 − 7.22 (t, *J* = 8.3 Hz, 1H), 7.06 (s, 1H), 6.86 − 6.83 (d, *J* = 9 Hz, 1H), 6.71 − 6.67 (m, 1H), 6.59 − 6.56 (d, *J* = 9 Hz, 1H), 6.33(s, 1H), 3.00(s, 6H). ^13^C NMR (151 MHz, DMSO-*d*_6_) *δ* 166.95, 159.25, 151.87, 151.21, 140.12, 137.53, 129.32, 111.80, 110.89, 109.42, 108.78, 105.32.

**16s**: 65 mg, yield: 22%. White solid. ^1^H NMR (300 MHz, DMSO-*d*_6_) *δ* 10.21 (s, 1H), 8.12 − 8.14 (dd, 1H, *J* = 1.5, 4.8 Hz), 8.02 − 8.05 (dd, 1H, *J* = 1.5, 4.8 Hz), 7.71 − 7.72 (d, 1H, *J* = 2.4 Hz), 7.62 − 7.66 (dd, 1H, *J* = 2.7, 8.7 Hz), 7.35 − 7.38 (d, 1H, *J* = 8.4 Hz), 6.98 (s, 2H), 6.64 − 6.68 (dd, 1H, *J* = 4.8, 7.8 Hz), 3.26 − 3.30 (m, 1H), 1.22 (s, 3H), 1.20 (s, 3H).

**16t**: 48 mg, yield: 14%. White solid. Melting point:198.1 °C. ^1^H NMR (300 MHz, CDCl_3_-*d*_1_) *δ* 8.19 − 8.21 (dd, 1H, *J* = 1.5, 4.8 Hz), 7.76 − 7.79 (dd, 2H, *J* = 1.5, 7.5 Hz), 7.50 − 7.53 (d, 1H, *J* = 8.7 Hz), 7.41 − 7.42 (d, 1H, *J* = 2.7 Hz), 7.34 − 7.37 (dd, 1H, *J* = 1.5, 8.4 Hz), 6.64 − 6.69 (dd, 1H, *J* = 1.8, 7.5 Hz), 6.37 (s, 2H), 3.31 − 3.41 (m, 1H), 1.26 (s, 3H), 1.24 (s,3H). ^13^C NMR (75 MHz, DMSO-*d*_6_) *δ* 167.07, 159.25, 152.23, 147.17, 139.44, 137.68, 132.88, 120.44, 119.42, 117.55, 111.79, 110.32, 33.00, 23.08.

**16u**: 195 mg, yield: 76%. White solid. ^1^H NMR (300 MHz, DMSO-*d*_6_) *δ* 10.15 (s, 1H), 8.16 (s, 1H), 7.80 (d, *J* = 5.1 Hz, 1H), 7.63 − 7.45 (m, 3H), 7.24 (t, *J* = 7.8 Hz, 1H), 6.99 (d, *J* = 7.7 Hz, 1H), 6.36 (s, 2H), 2.94−2.74 (m, 1H), 1.20 (d, *J* = 6.9 Hz, 6H). ^13^C NMR (75 MHz, DMSO-*d*_6_) *δ* 166.57, 149.30, 144.76, 140.48, 139.11, 136.05, 128.92, 122.49, 121.57, 120.36, 119.13, 118.76, 33.97, 24.33.

**16v**: 203 mg, yield: 80%. Semi-solid. ^1^H NMR (300 MHz, DMSO-*d*_6_) *δ* 10.14 (s, 1H), 7.69 − 7.49 (m, 3H), 7.35 − 7.18 (m, 2H), 6.98 (d, *J* = 7.7 Hz, 1H), 6.69 (dd, *J* = 8.3, 0.7 Hz, 1H), 6.28 (s, 2H), 2.96 − 2.75 (m, 1H), 1.20 (d, *J* = 6.9 Hz, 6H). ^13^C NMR (151 MHz, DMSO-*d*_6_) *δ* 162.97, 159.02, 149.68, 148.16, 138.95, 138.65, 129.25, 122.30, 117.80, 117.38, 112.28, 110.63, 33.96, 24.30.

**16w**: 189 mg, yield: 74%. Semi-solid. ^1^H NMR (300 MHz, DMSO-*d*_6_) *δ* 10.33 (s, 1H), 7.87 (dd, *J* = 4.1, 1.4 Hz, 1H), 7.76 − 7.58 (m, 2H), 7.41 – 7.14 (m, 3H), 7.05 − 6.78 (m, 3H), 2.92−2.79 (m, 1H), 1.20 (d, *J* = 6.9 Hz, 6H). ^13^C NMR (151 MHz, DMSO-*d*_6_) *δ* 166.22, 149.46, 147.35, 138.80, 136.05, 129.01, 128.59, 128.23, 125.43, 122.00, 118.32, 117.81, 33.99, 24.33.

**16x**: 207 mg, yield: 81%. White solid. ^1^H NMR (300 MHz, DMSO-*d*_6_) *δ* 10.20 (s, 1H), 8.04 (d, *J* = 5.2 Hz, 1H), 7.67 − 7.52 (m, 2H), 7.24 (t, *J* = 7.8 Hz, 1H), 6.98 (d, *J* = 7.7 Hz, 1H), 6.90 (dd, *J* = 5.3, 1.5 Hz, 1H), 6.85 (s, 1H), 6.19 (s, 2H), 2.94 − 2.76 (m, 1H), 1.19 (d, *J* = 6.9 Hz, 6H). ^13^C NMR (75 MHz, DMSO-*d*_6_) *δ* 165.32, 160.71, 149.34, 148.85, 143.96, 139.28, 128.97, 122.47, 118.79, 118.43, 109.86, 106.63, 33.95, 24.32.

**16y**: 214 mg, yield: 84%. White solid. ^1^H NMR (300 MHz, DMSO-*d*_6_) *δ* 9.82 (s, 1H), 8.58 (d, *J* = 2.2 Hz, 1H), 7.91 (dd, *J* = 8.7, 2.5 Hz, 1H), 7.66 − 7.50 (m, 2H), 7.21 (t, *J* = 7.8 Hz, 1H), 6.93 (d, *J* = 7.6 Hz, 1H), 6.59 (s, 2H), 6.46 (d, *J* = 8.8 Hz, 1H), 2.95 − 2.74 (m, 1H), 1.19 (d, *J* = 6.9 Hz, 6H). ^13^C NMR (75 MHz, DMSO-*d*_6_) *δ* 164.67, 162.11, 149.40, 149.19, 139.88, 136.99, 128.83, 121.76, 118.73, 118.62, 118.28, 107.25, 33.97, 24.34.

**16z**: 206 mg, yield: 81%. White solid. ^1^H NMR (300 MHz, DMSO-*d*_6_) *δ* 10.09 (s, 1H), 8.61 (s, 1H), 8.04 (d, *J* = 5.8 Hz, 1H), 7.59 (s, 1H), 7.52 (d, *J* = 8.1 Hz, 1H), 7.23 (t, *J* = 7.8 Hz, 1H), 7.10 − 6.91 (m, 3H), 6.64 (d, *J* = 5.8 Hz, 1H), 2.90 − 2.80 (m, 1H), 1.19 (d, *J* = 6.9 Hz, 6H). ^13^C NMR (151 MHz, DMSO-*d*_6_) *δ* 166.69, 154.99, 150.48, 149.70, 149.27, 139.32, 128.89, 122.25, 119.02, 118.65, 112.37, 110.98, 33.97, 24.33.

**17**: 213 mg, yield 83%. White solid. ^1^H NMR (300 MHz, DMSO-*d*_6_) *δ* 10.46 (s, 1H), 8.65 (d, *J* = 4.9 Hz, 1H), 7.69 (d, *J* = 4.9 Hz, 1H), 7.61 − 7.48 (m, 2H), 7.27 (t, *J* = 7.8 Hz, 1H), 7.10 − 6.88 (m, 3H), 2.96 − 2.77 (m, 1H), 1.20 (d, *J* = 6.9 Hz, 6H). ^13^C NMR (151 MHz, DMSO-*d*_6_) *δ* 165.13, 158.57, 149.43, 143.00, 138.78, 129.05, 125.59, 122.93, 119.04, 118.71, 115.60, 33.95, 24.31.

**18a**: 227 mg, yield: 84%. White solid. ^1^H NMR (300 MHz, DMSO-*d*_6_) *δ* 9.72 (s, 1H), 8.52 (s, 1H), 7.46 (dd, *J* = 21.3, 13.2 Hz, 4H), 7.20 (t, *J* = 7.8 Hz, 1H), 6.92 (d, *J* = 7.7 Hz, 1H), 6.61 (s, 2H), 2.92 − 2.74 (m, 1H), 1.19 (d, *J* = 6.9 Hz, 6H). ^13^C NMR (75 MHz, DMSO-*d*_6_) *δ* 166.23, 164.08, 163.79, 158.58, 149.16, 139.63, 128.79, 121.75, 118.97, 118.62, 99.57, 33.97, 24.34.

**18b**: 216 mg, yield: 84%. White solid. ^1^H NMR (300 MHz, DMSO-*d*_6_) *δ* 10.23 (s, 1H), 8.72 (s, 1H), 8.47 (s, 1H), 7.69 (s, 2H), 7.56 (s, 1H), 7.54 − 7.46 (m, 1H), 7.25 (t, *J* = 7.8 Hz, 1H), 6.99 (d, *J* = 7.7 Hz, 1H), 2.94 − 2.77 (m, 1H), 1.19 (d, *J* = 6.9 Hz, 6H). ^13^C NMR (75 MHz, DMSO-*d*_6_) *δ* 165.32, 162.23, 160.21, 155.85, 149.34, 139.03, 128.96, 122.56, 119.11, 118.76, 109.21, 33.96, 24.32.

**19**: 207 mg, yield: 81%. White solid. Melting point: 81.5 °C. ^1^H NMR (300 MHz, DMSO-*d*_6_) *δ* 10.39 (s, 1H), 8.27 (d, *J* = 2.3 Hz, 1H), 7.90 (d, *J* = 2.3 Hz, 1H), 7.75 − 7.44 (m, 4H), 7.24 (t, *J* = 7.8 Hz, 1H), 6.98 (d, *J* = 7.7 Hz, 1H), 2.96 − 2.76 (m, 1H), 1.20 (d, *J* = 6.9 Hz, 6H). ^13^C NMR (151 MHz, DMSO-*d*_6_) *δ* 164.87, 155.89, 149.42, 147.77, 138.56, 131.38, 128.98, 125.86, 122.46, 118.82, 118.30, 33.98, 24.31.

**20**: 218 mg, yield: 83%. White solid. ^1^H NMR (300 MHz, DMSO-*d*_6_) *δ* 9.43 (s, 1H), 8.90 (s, 1H), 7.52 − 7.44 (m, 2H), 7.23 − 7.14 (m, 1H), 7.02 (s, 2H), 6.92 (d, *J* = 7.7 Hz, 1H), 2.93 − 2.74 (m, 1H), 1.19 (d, *J* = 6.9 Hz, 6H). ^13^C NMR (75 MHz, DMSO-*d*_6_) *δ* 164.40, 162.94, 155.82, 149.12, 139.52, 128.74, 121.83, 119.18, 118.83, 93.85, 33.96, 24.34.

**21**: 192 mg, yield: 78%. White solid. ^1^H NMR (300 MHz, DMSO-*d*_6_) *δ* 10.30 (s, 1H), 8.09 (dd, *J* = 4.8, 1.7 Hz, 1H), 8.05 − 7.93 (m, 2H), 7.54 (s, 1H), 7.05 (s, 2H), 6.63 (dd, *J* = 7.7, 4.8 Hz, 1H), 4.53 − 4.40 (m, 1H), 1.39 (d, *J* = 6.7 Hz, 6H). ^13^C NMR (75 MHz, DMSO-*d*_6_) *δ* 165.20, 159.31, 151.92, 136.99, 130.39, 121.59, 119.01, 111.82, 109.89, 53.44, 23.09.

**22a**: 124 mg, yield: 69%. White solid. ^1^H NMR (300 MHz, CDCl_3_-*d*_1_) *δ* 8.11 − 8.14 (dd, 1H, *J* = 1.6, 4.9 Hz), 7.57 − 7.60 (dd, 1H, *J* = 1.7, 7.6 Hz), 6.58 − 6.62 (dd, 1H, *J* = 5.0, 7.8 Hz), 6.44 (s, 2H), 5.84 (s, 1H), 4.18 − 4.27 (m, 1H), 1.27 (s, 3H), 1.25 (s, 3H). ^13^C NMR (151 MHz, DMSO-*d*_6_) *δ* 166.91, 158.77, 150.46, 137.45, 111.66, 110.90, 41.23, 22.68.

**22b**: 132 mg, yield: 68%. White solid. ^1^H NMR (300 MHz, CDCl_3_-*d*_1_) *δ* 8.10 − 8.13 (dd, 1H, *J* = 1.7, 74.8 Hz), 7.52 − 7.55 (dd, 1H, *J* = 1.7, 7.6 Hz), 6.56 − 6.60 (dd, 1H, *J* = 5.0, 7.8 Hz), 6.28 (s, 2H), 5.82 (s, 1H), 1.45 (s, 9H). ^13^C NMR (151 MHz, DMSO-*d*_6_) *δ* 168.20, 159.05, 151.01, 137.36, 111.76, 111.66, 51.34, 29.01.

**22c**: 173 mg, yield: 79%. White solid. ^1^H NMR (300 MHz, CDCl_3_-*d*_1_) *δ* 8.12 − 8.14 (dd, 1H, *J* = 1.7, 5.1 Hz), 7.55 − 7.58 (dd, 1H, *J* = 1.6, 7.6 Hz), 6.57 − 6.61 (dd, 1H, *J* = 4.9, 7.6 Hz), 6.35 (s, 2H), 5.86 − 5.88 (d, 1H, *J* = 4.0 Hz), 3.85 − 3.97 (m, 1H), 1.92 − 2.03 (m, 2H), 1.72 − 1.79 (m, 2H), 1.62 − 1.69 (m, 2H), 1.15 − 1.49 (m, 4H). ^13^C NMR (75 MHz, DMSO-*d*_6_) *δ* 167.13, 159.24, 151.36, 136.93, 111.63, 110.50, 48.54, 32.79, 25.71, 25.39.

**22d**: 246 mg, yield: 81%. White solid. ^1^H NMR (300 MHz, CDCl_3_-*d*_1_) *δ* 8.12 − 8.14 (dd, 1H, *J* = 1.6, 4.8 Hz), 7.57 − 7.59 (d, 1H, *J* = 7.6 Hz), 6.57 − 6.62 (dd, 1H, *J* = 5.0, 7.6 Hz), 6.39 (s, 2H), 6.02 (s, 1H), 3.36 − 3.43 (m, 2H), 1.25 − 1.61 (m, 20H), 0.85 − 0.89 (m, 3H). ^13^C NMR (151 MHz, DMSO-*d*_6_) *δ* 167.85, 159.26, 151.45, 136.68, 111.67, 110.26, 31.76, 29.51, 29.46, 29.24, 29.18, 26.95, 22.56, 14.40.

### 3.2. Cytotoxicity Tests

The cytotoxic effect of compounds on HUVEC’s viability was assessed by the CCK-8 (Target Molecule Corp., Boston, MA, USA) assay as described previously [18,19]. Briefly, HUVECs were diluted with DMEM complete mediums to 5 × 10^6^ cells/mL, and 200 μL of cell suspension was added to the 96-well plates. After adhesion for 3 h, the cell supernatant was replaced with fresh 100 μL DMEM complete medium containing different concentrations of antifungal agents. Furthermore, the HUVECs were cultured for 24 h at 37 °C with 5% CO_2_. Finally, 10 μL of CCK-8 regent were added to the 96-well plates and incubated for another 2 h. The OD_450_ was measured by the microplate reader.

### 3.3. Drug Susceptibility Testing

MIC was determined in RPMI 1640 medium for 24 h as mentioned in CLSI M27-A [12,20]. Briefly, *C. albicans* or *C. neoformans* were cultured in a YPD medium for 18 h or overnight. The fungi were washed with PBS three times. The fungal suspension was adjusted to 5 × 10^3^ CFU/mL in RPMI 1640 medium. Furthermore, the fungal suspension was added to the 96 well plates. Antifungal compounds were dissolved in DMSO and added to the first column and diluted two-fold serially. Fungal cells were cultured at 30 °C for 24 h and OD_630_ was measured by the microplate reader.

### 3.4. Hyphae Formation Assay

The in vitro hyphae formation assay was determined as described previously [15,16]. *C. albicans* SC5314 was cultured in a YPD medium for 18 h. Exponentially growing cells were diluted with RPMI 1640 medium to 5 × 10^5^ CFU/mL and transferred to 24-well plates. Various concentrations of **16g** were added to the fungal suspension. Finally, the cellular morphology was photographed after incubation at 37 °C for 3 h.

### 3.5. Biofilm Formation Assay

The in vitro biofilm formation assay was performed as described previously [21,22]. In brief, 100 μL of 1 × 10^6^ CFU/mL of *C. albicans* in RPMI 1640 medium were added to a 96-well tissue culture plate and incubated for 90 min at 37 °C. After adhesion, the suspension and non-adherent cells were removed. 150 μL of fresh RPMI 1640 or RPMI 1640 containing different concentrations of compounds **16g** was added. The plate was further incubated at 37 °C for 24 h until the formation of mature biofilms. After incubation, each well was washed with PBS for three times. The formed biofilms were cultured at 37 °C for 3 h with 150 μL of XTT reagents, which contained 0.5 mg/mL of XTT and 1 μM menadione. After incubation, the OD_490_ was measured by the microplate reader.

### 3.6. Time-Kill Curve Studies

The time-kill studies were performed as previously described [23,24,25]. *C. albicans* SC5314 was cultured in a YPD medium for 16–20 h. Cells were adjusted to 1 × 10^6^ CFU/mL in RPMI 1640. Furthermore, 1 mL of the fungal suspension was added to 9 mL of fresh RPMI 1640 and incubated at 30 °C with shaking at 200 rpm. Compound **16g** was dissolved in DMSO and added at each tube to the final concentrations of 0.25, 0.5, and 1 μg/mL. After incubation for 6, 12, 18, and 24 h, 100 μL of fungal suspensions from each tube were serially diluted in PBS and plated onto SDA agar. Finally, the plates were incubated at 30 °C for 48 h, and the *C. albicans* colonies were counted.

### 3.7. PAINS Screening and ADME/T Prediction

The Swiss ADME software (www.swissadme.ch, accessed on 25 May 2022) of the Swiss Institute of Bioinformatics was accessed through a web server displaying the Swiss ADME submission page and was used to evaluate the presence of chemical species belonging to the “Pan-Assay Interference Compounds” (PAINS) chemical class, implemented from the paper by Baell et al. and estimate individual ADME properties of compounds. The list contains one input for each molecule, as defined by the Simplified Molecular Input Line Input System (SMILES), and the results for each molecule are displayed in tables and Excel spreadsheets [13,26,27].

## 4. Conclusions

In summary, a hit-to-lead optimization was preliminarily performed in this study, and a series of 37 nicotinamide derivatives have been designed, synthesized, and their antifungal activities evaluated. Particularly, compound **16g** exhibited excellent to moderate in vitro antifungal activity against species of *Candida* and *Cryptococcus*, including fluconazole-resistant *C. albicans* strains. In addition, potent anti-hyphal and anti-biofilm effects of compound **16g** were also observed. Mechanistically, our results indicated that **16g** inhibited the growth of *C. albicans* by disrupting the cell wall. In silico ADMET prediction suggests antifungal compound **16g** is a good lead. Further structural optimization and the mechanisms of action of compound **16g** are currently under investigation.

## Figures and Tables

**Table 1:** In vitro antifungal activities of nicotinamide derivatives against *C. albicans* SC5314.

Compd	Structure	MIC (μg/mL)	Compd	Structure	MIC (μg/mL)
		*C. alb* SC5314			*C. alb* SC5314
**7**	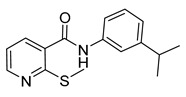	16	**16f**	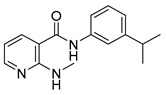	>16
**16a**	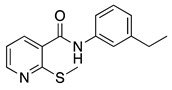	>16	**16g**	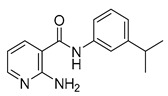	0.25
**16b**	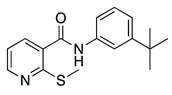	>16	**16h**	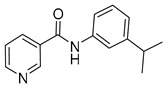	>16
**16c**	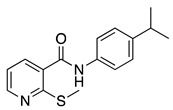	>16	FLC	-	0.25
**16d**	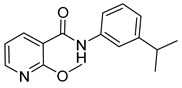	>16	**1**	-	>16
**16e**	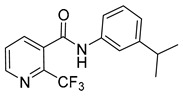	>16	**3**	-	>16

Abbreviations: MIC: minimum inhibitory concentration; *C. alb*: *Candida albicans*; FLC: fluconazole.

**Table 2:** In vitro antifungal activities of compounds against *C. albicans* SC5314.

Compd.	Structure	MIC (μg/mL)	Compd.	Structure	MIC (μg/mL)
		*C. alb* SC5314			*C. alb* SC5314
**16g**	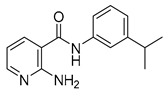	0.25	**16w**	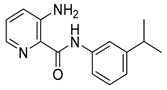	64
**16i**	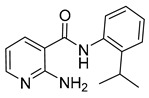	>64	**16x**	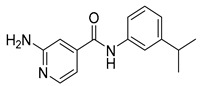	>64
**16j**	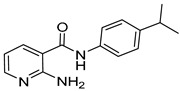	0.5	**16y**	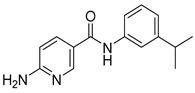	>64
**16k**	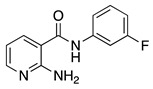	>64	**16z**	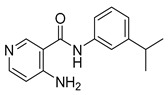	16
**16l**	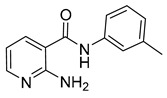	16	**17**	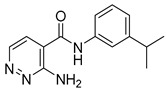	64
**16m**	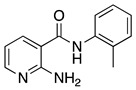	>64	**18a**	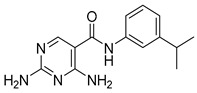	16
**16n**	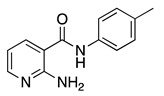	64	**18b**	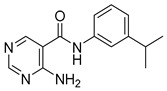	64
**16o**	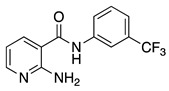	>64	**19**	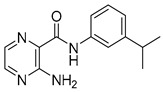	4
**16p**	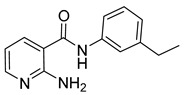	4	**20**	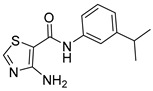	>64
**16q**	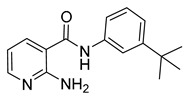	>64	**21**	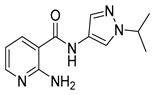	>64
**16r**	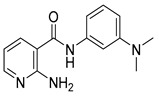	16	**22a**	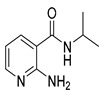	>64
**16s**	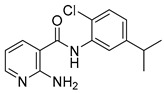	8	**22b**	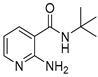	>64
**16t**	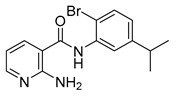	4	**22c**	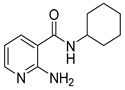	>64
**16u**	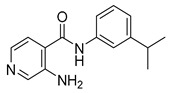	32	**22d**	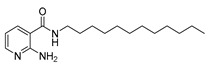	>64
**16v**	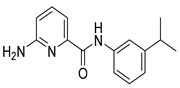	>64	FLC	-	0.25

Abbreviations: MIC: minimum inhibitory concentration; *C. alb*: *Candida albicans*; FLC: fluconazole.

**Table 3:** In vitro antifungal activities against drug-resistant *C. albicans* of **16g** and FLC.

*C. albicans*	Isolate	MIC (μg/mL)	
		16g	FLC
fluconazole-sensitive (2)	Y0109	0.125	0.125
	465	0.5	0.125
fluconazole-resistant (6)	862	0.5	>64
	786	0.5	>64
	100	1	>64
	385	0.125	>64
	898	0.5	>64
	504	0.25	>64

Abbreviations: MIC: minimum inhibitory concentration; *C. albicans*, *Candida albicans*; FLC: fluconazole.

**Table 4:** In vitro antifungal activities of **16g** and FLC.

Species	Isolate	MIC (μg/mL)	
		16g	FLC
*C. parapsilosis*	22019	4	1
	660	2	0.25
*C. krusei*	463	8	4
	629	16	2
*C. glabrata*	537	8	0.5
*C. tropicalis*	752	0.5	0.5
	112936	2	2
*C. neoformans*	32609	32	1
	34877	16	2
	56992	8	1
*A. fumigatus*	7544	>32	>64
	023-2	>32	>64
*T.mentagrophyton*	T5A	16	16
	T5B	32	16
	T5E	32	16

Abbreviations: MIC: minimum inhibitory concentration; *C. parapsilosis*, *Candida parapsilosis*; *C. krusei, Candida krusei*; *C. glabrata*, *Candida glabrata*; *C. tropicalis*, *Candida tropicalis*; *C. neoformans*, *Cryptococcus neoformans*; *A. fumigatus*, *Aspergillus fumigatus*; *T. mentagrophyton*, *Trichophyton mentagrophyton*; FLC: fluconazole.

## Data Availability

Not applicable.

## Supplementary Materials

The following supporting information can be downloaded at: https://www.mdpi.com/article/10.3390/molecules28031135/s1. NMR spectra of target compounds; HPLC spectra of representative compounds.

## Sample Availability

Samples of the compounds are not available from the authors.
