# Supplementary material for: Design, Synthesis and Structure-Activity Relationship Studies of Nicotinamide Derivatives as Potent Antifungal Agents by Disrupting Cell Wall
Source: Molecules. 2023 Jan 23;28(3):1135. doi: 10.3390/molecules28031135 (PMC9919825; doi:10.3390/molecules28031135)
Supplement: Supplementary file 1 [file molecules-28-01135-s001.zip › molecules-2130093-supplementary.pdf]

Supporting Information

Design, Synthesis and Structure-Activity Relationship Studies of Nicotinamide Derivatives as Potent Antifungal Agents by Disrupting Cell Wall

Tingjunhong Ni [1,†], Fei Xie [2,†], Liping Li [1,†], Yumeng Hao [2], Xiaochen Chi [3], Lan Yan [2], Dazhi Zhang [1,2,*], Yuanying Jiang [1,*] and Quanzhen Lv [2,*]

[1] Department of Pharmacy, Shanghai Tenth People's Hospital, School of Medicine, Tongji University, No.1239 Siping Road, Shanghai 200092, China
[2] School of Pharmacy, Naval Medical University, No.325 Guohe Road, Shanghai 200433, China
[3] School of Chinese Materia Medica, Shenyang Pharmaceutical University, Shenyang 110016, China
* Correspondence: zdzhang_yjhx@smmu.edu.cn (D.Z.); 13761571578@163.com (Y.J.); lvquanzhen2011@163.com (Q.L.)
† These authors contributed equally to this work.

Table of Contents

NMR Spectra of Target Compounds……………………………………………..S2
HPLC Spectra of Representative Compounds…………….…………………..S38

NMR Spectra of Target Compounds

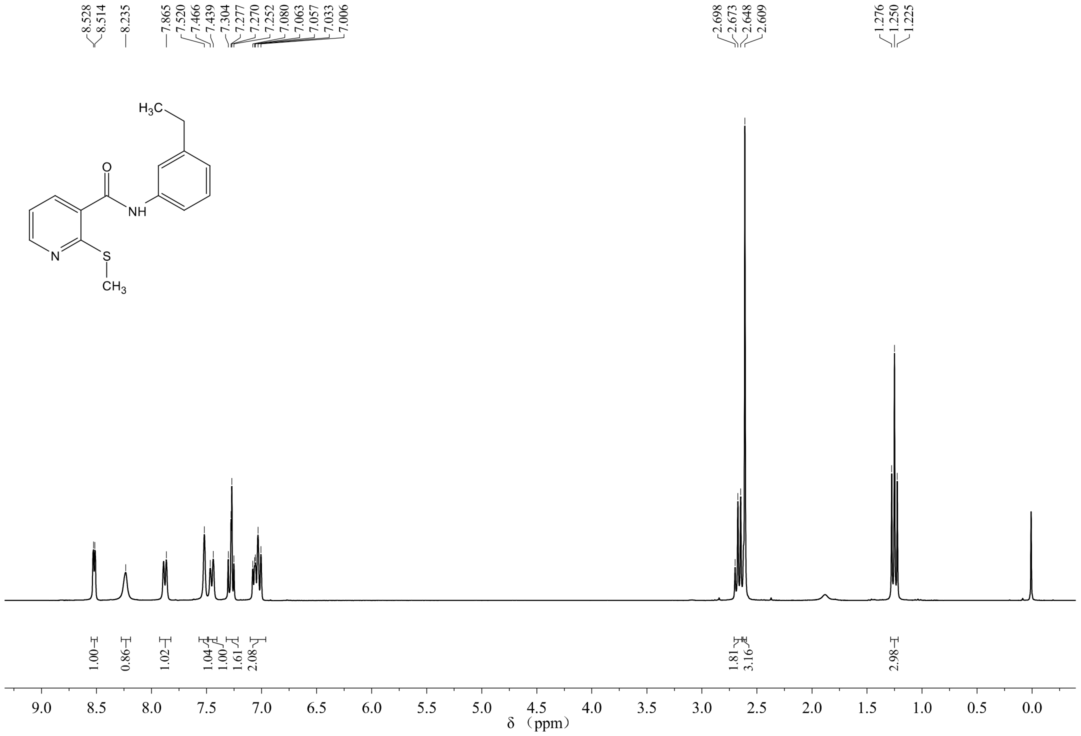

^{1}H NMR spectra of 16a

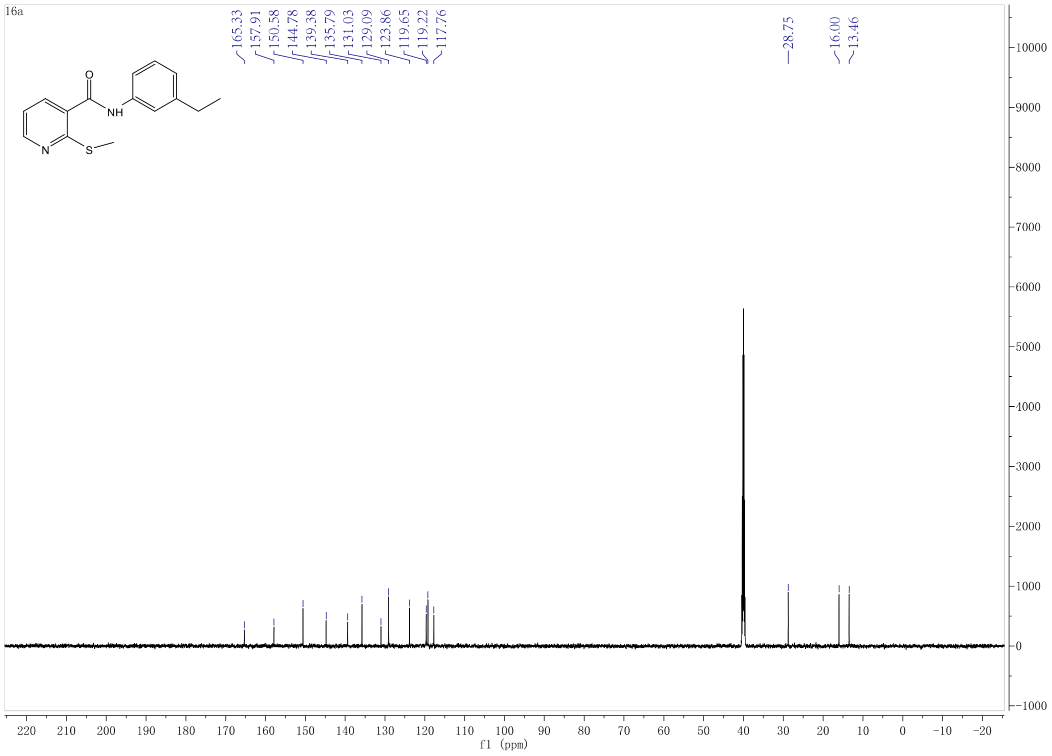

^{13}C NMR spectra of 16a

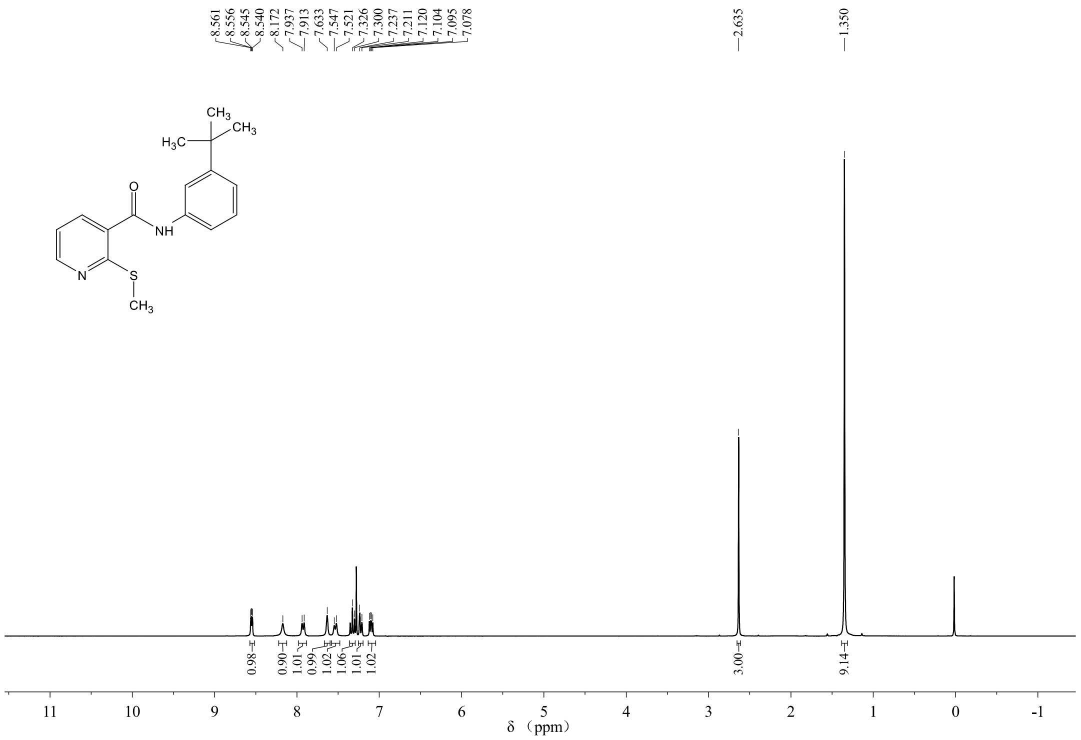

^{1}H NMR spectra of 16b

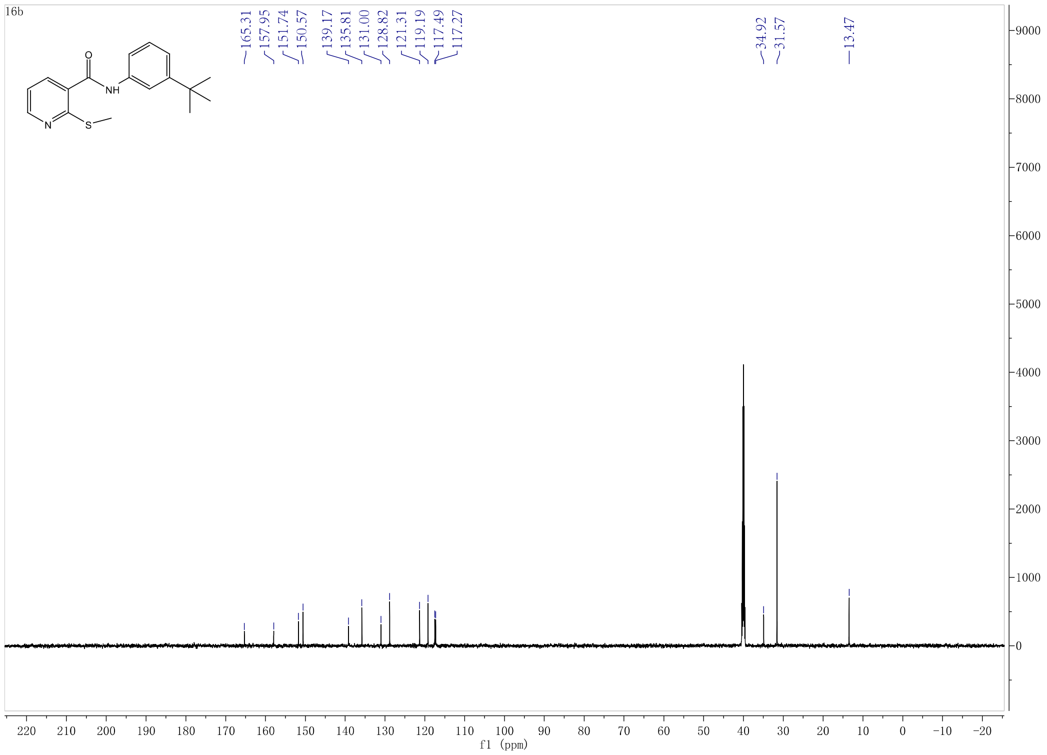

^{13}C NMR spectra of 16b

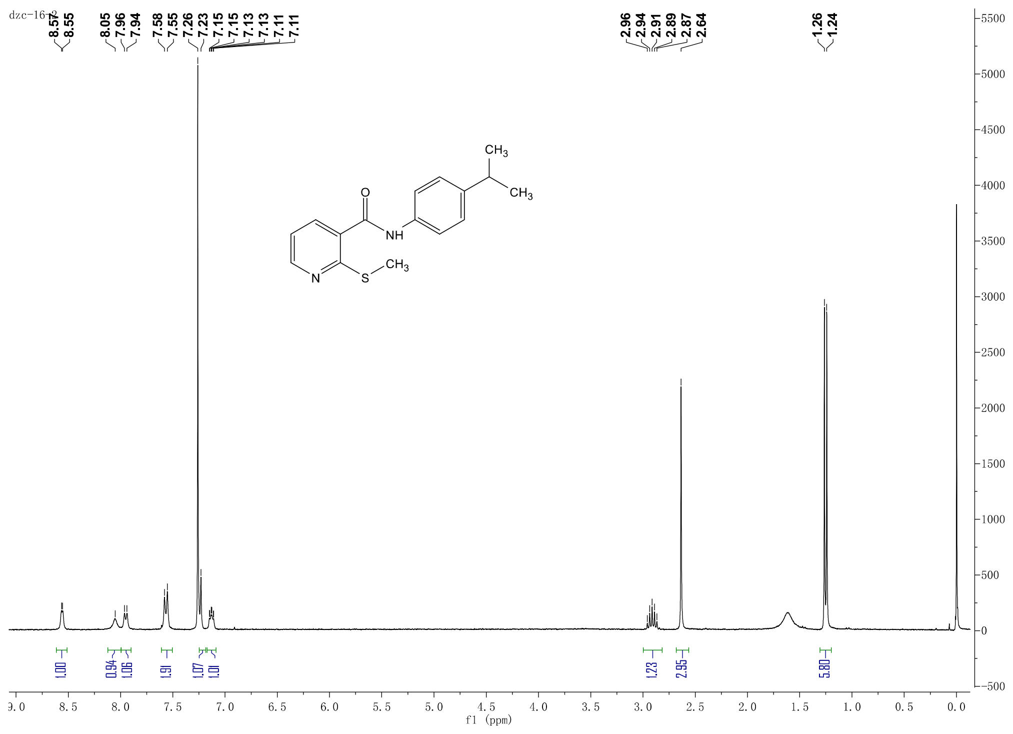

^1H NMR spectra of 16c

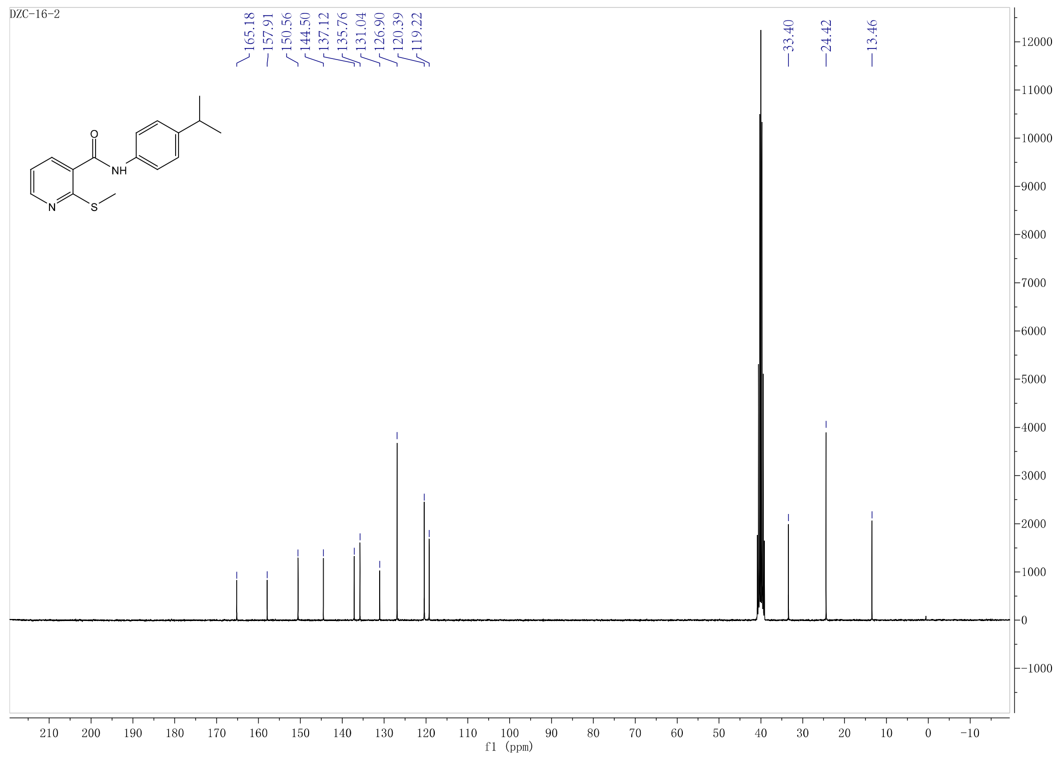

^{13}C NMR spectra of 16c

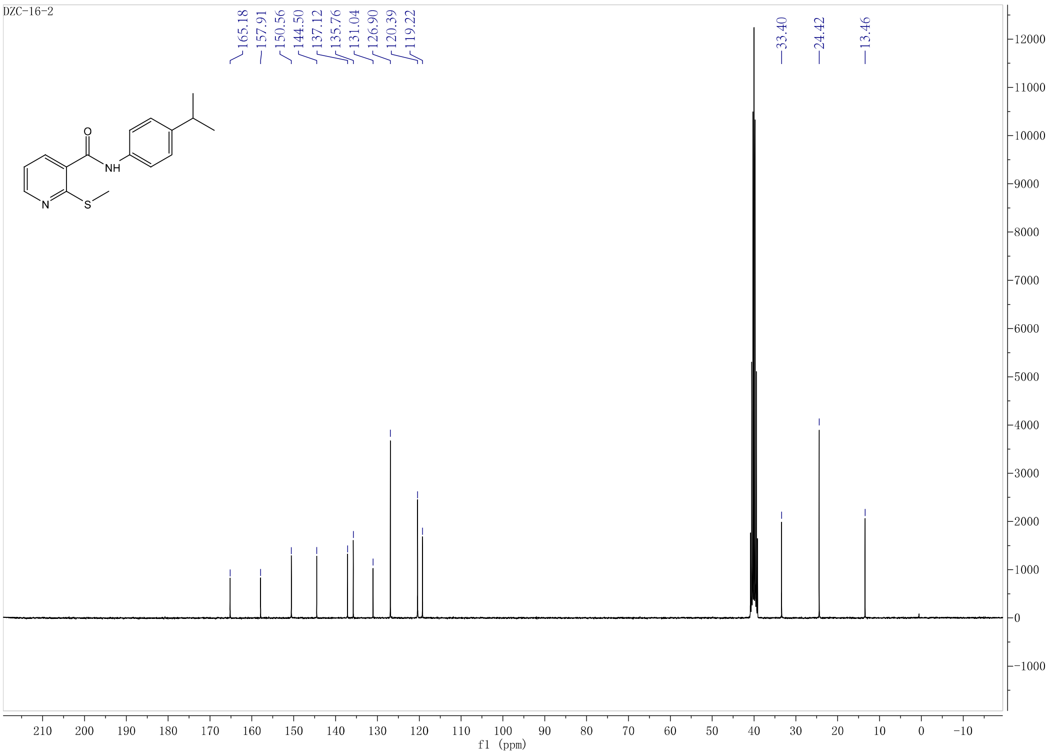

^{13}C NMR spectra of 16c

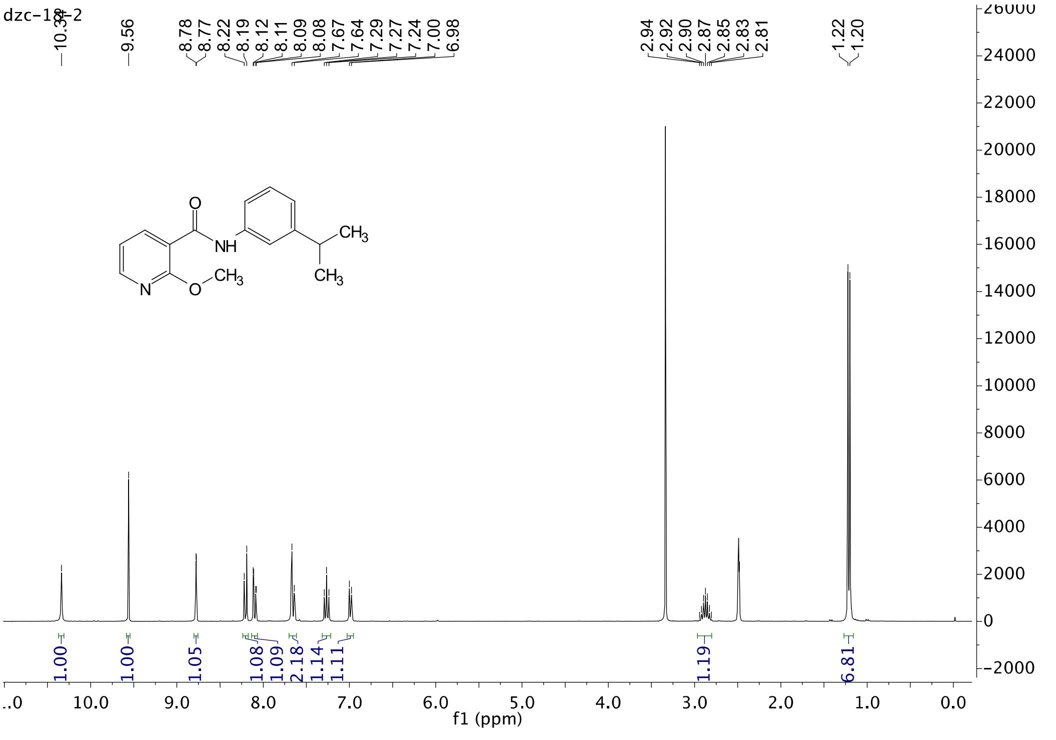

^{1}H NMR spectra of 16d

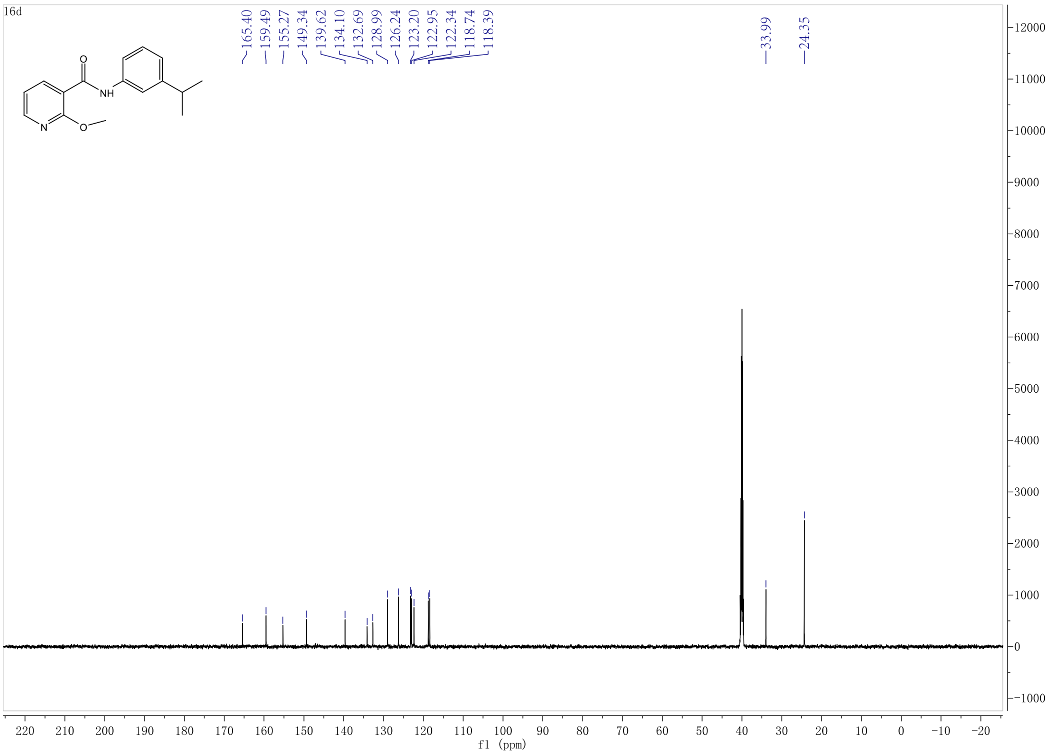

^{13}C NMR spectra of 16d

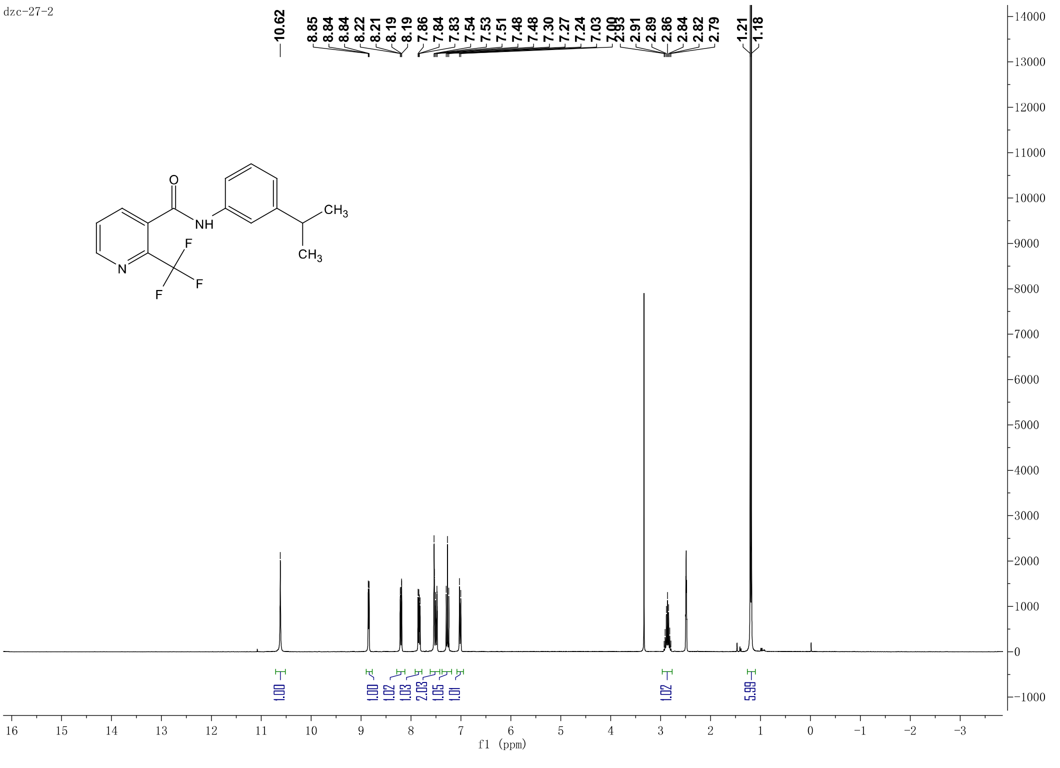

^{1}H NMR spectra of 16e

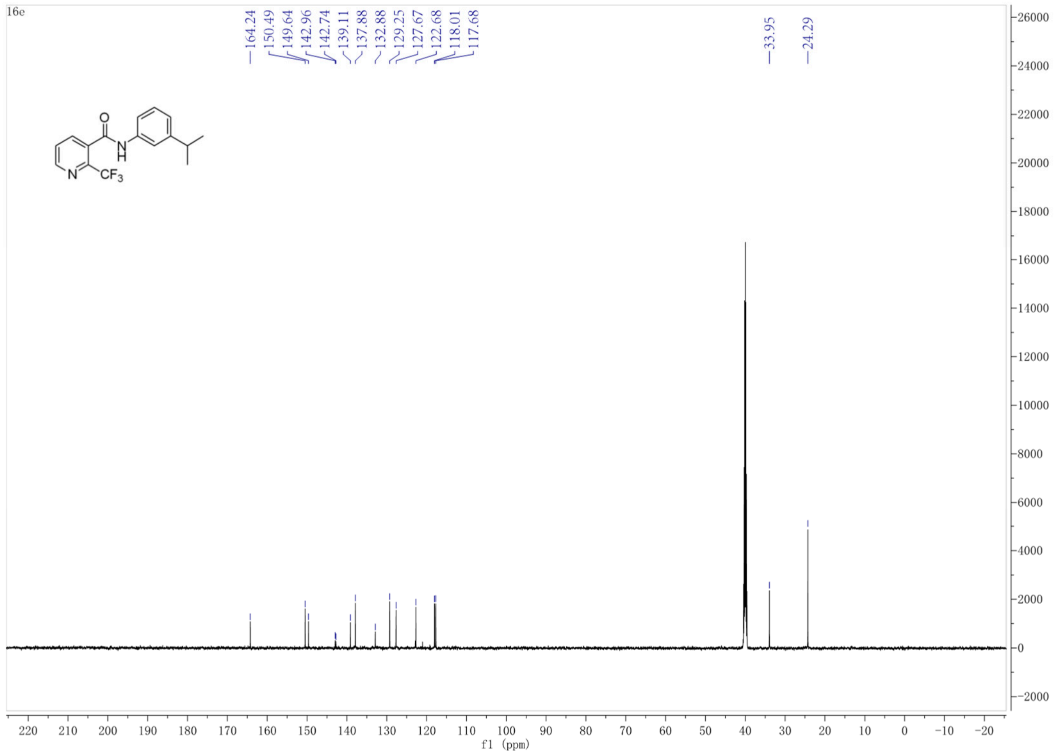

^{13}C NMR spectra of 16e

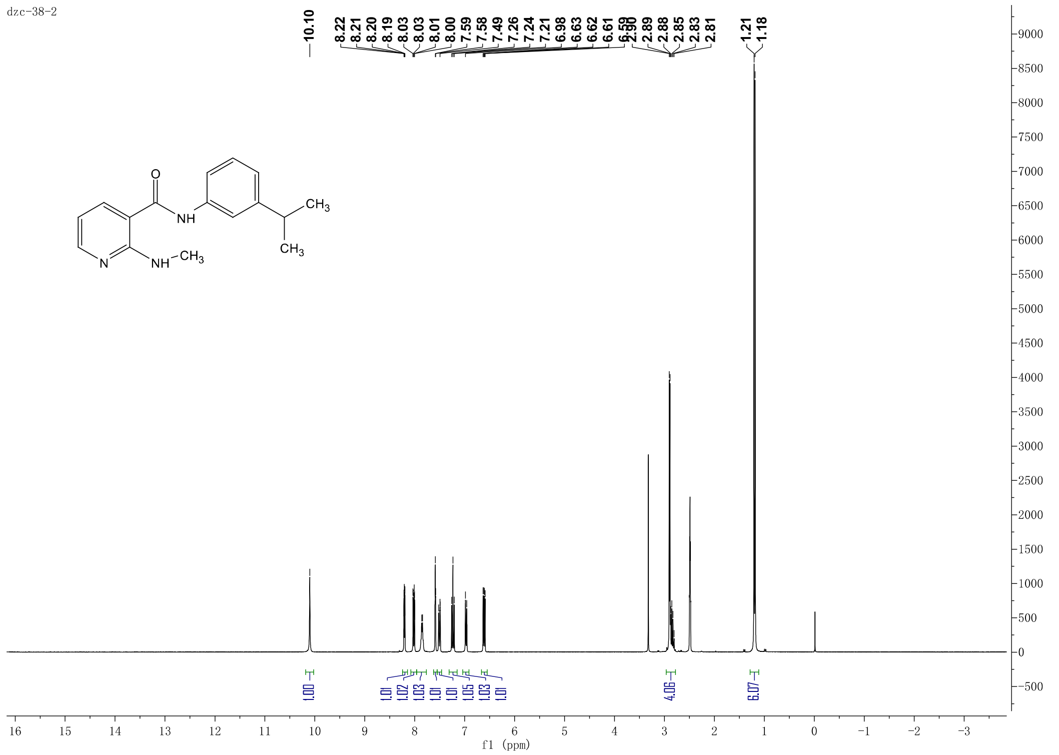

^{1}H NMR spectra of 16f

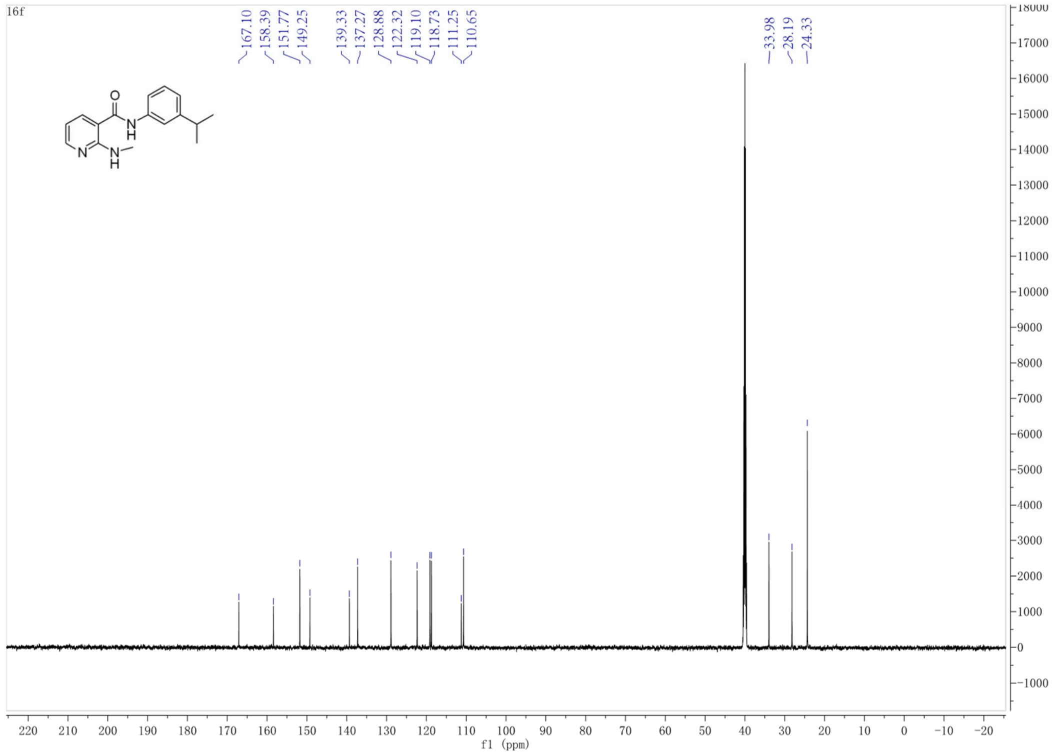

^{13}C NMR spectra of 16f

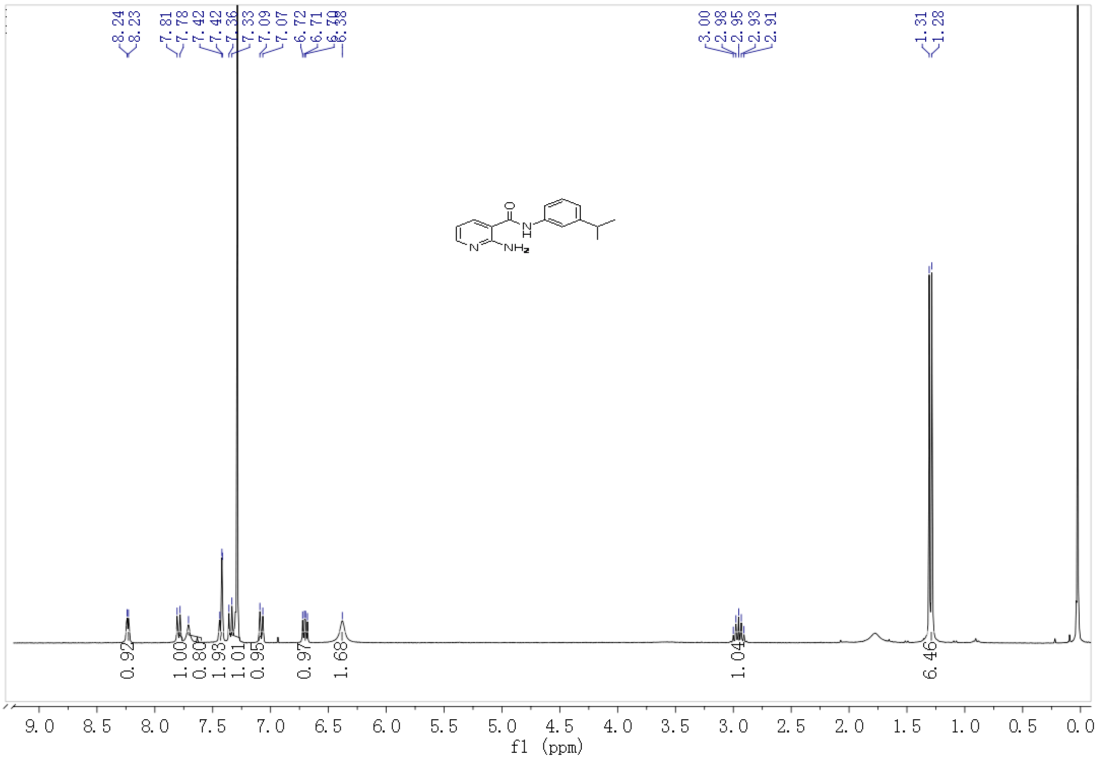

^{1}H NMR spectra of 16g

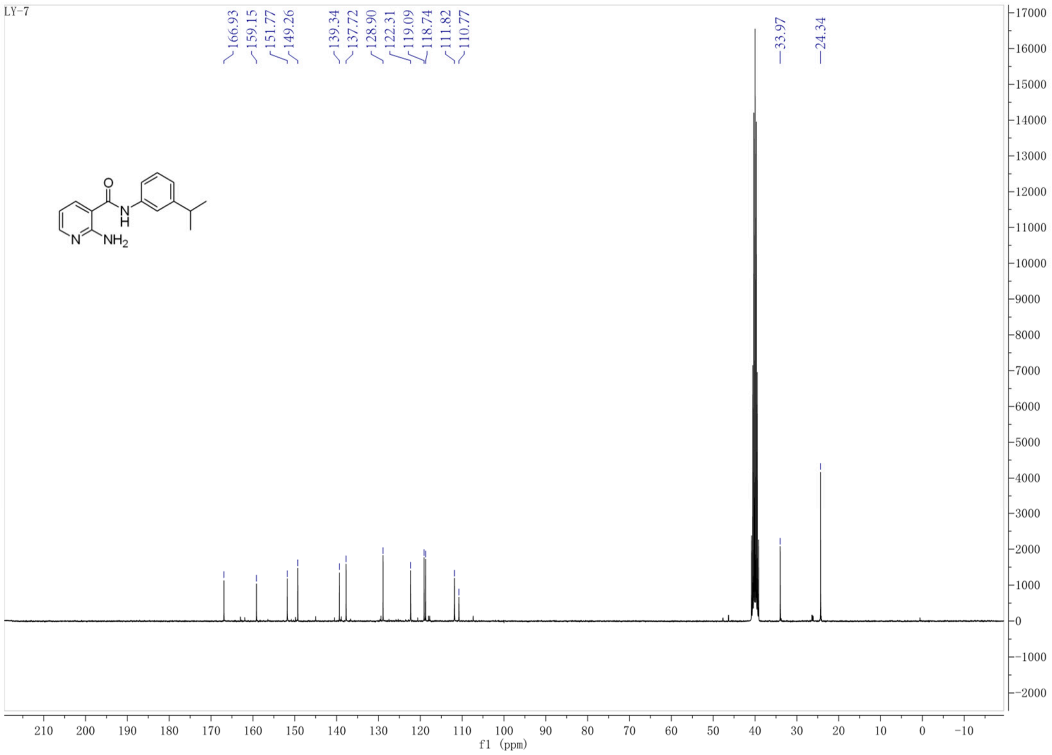

^{13}C NMR spectra of 16g

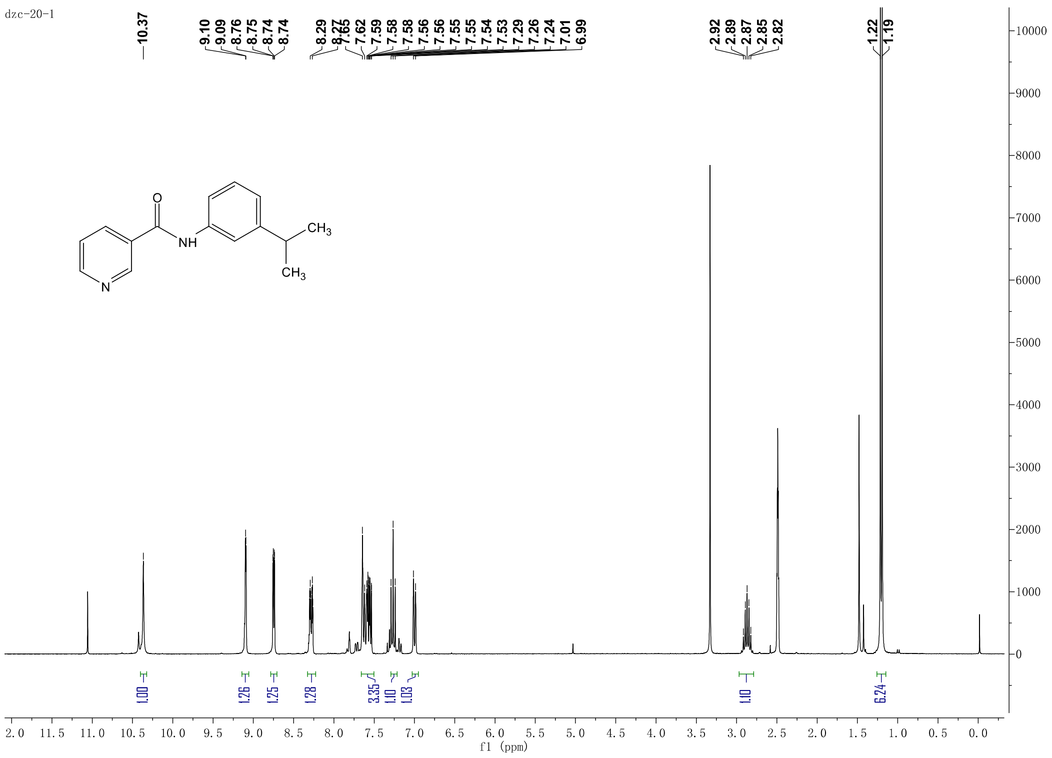

^{1}H NMR spectra of 16h

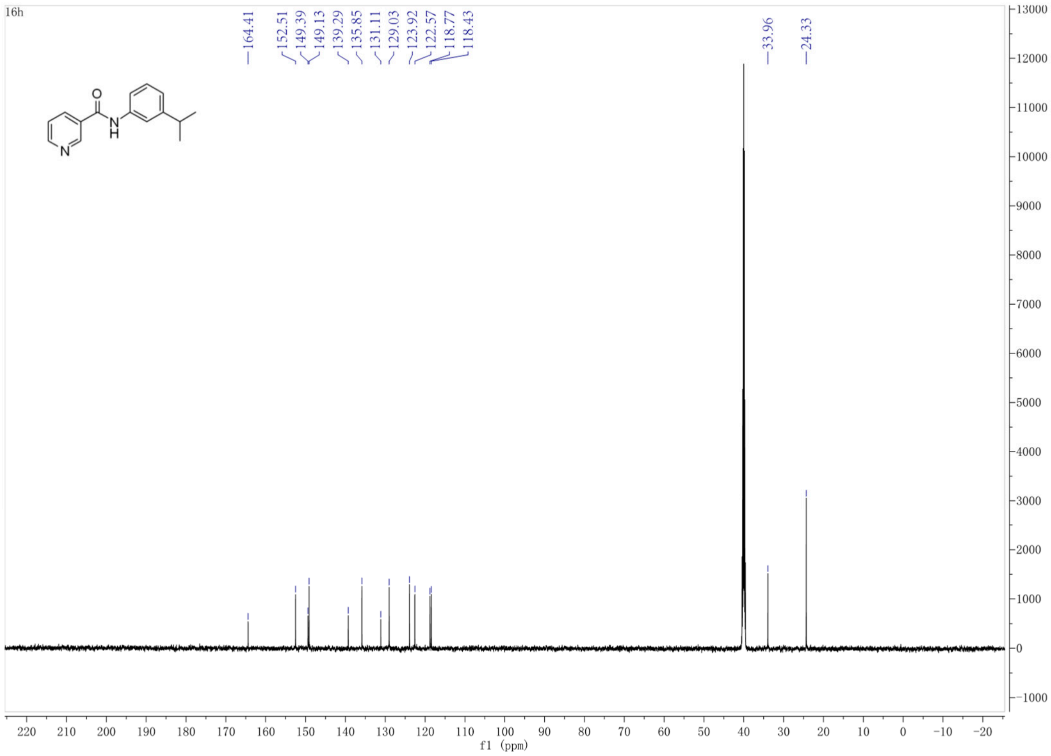

^{13}C NMR spectra of 16h

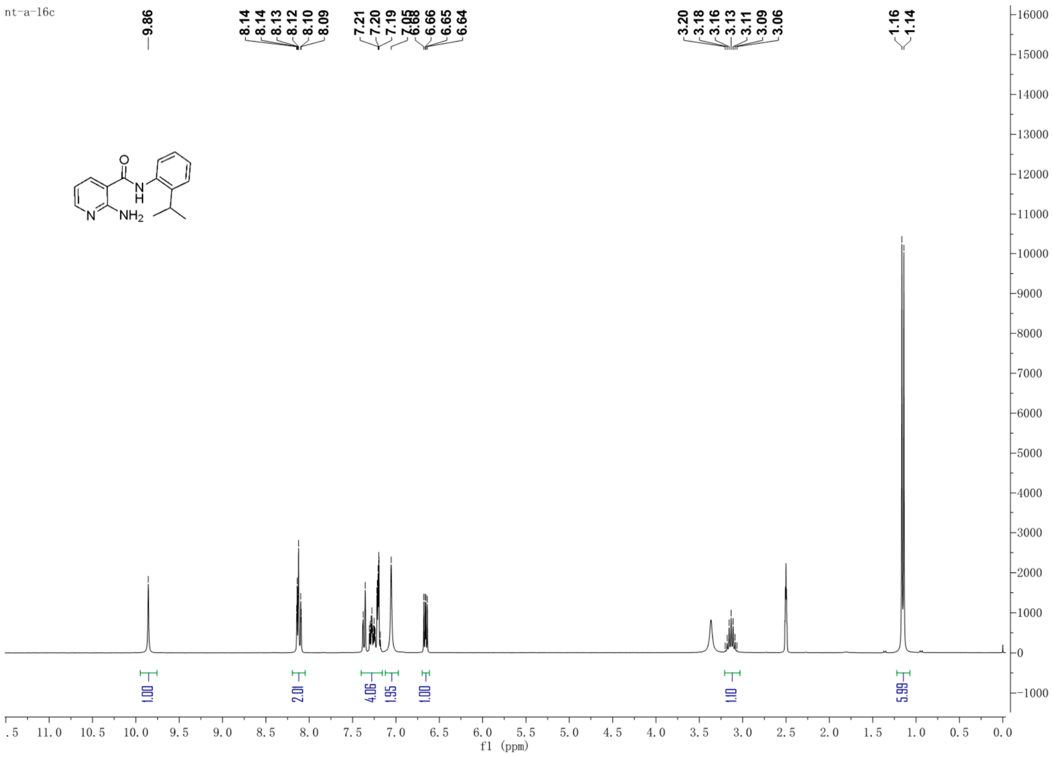

^{1}H NMR spectra of 16i

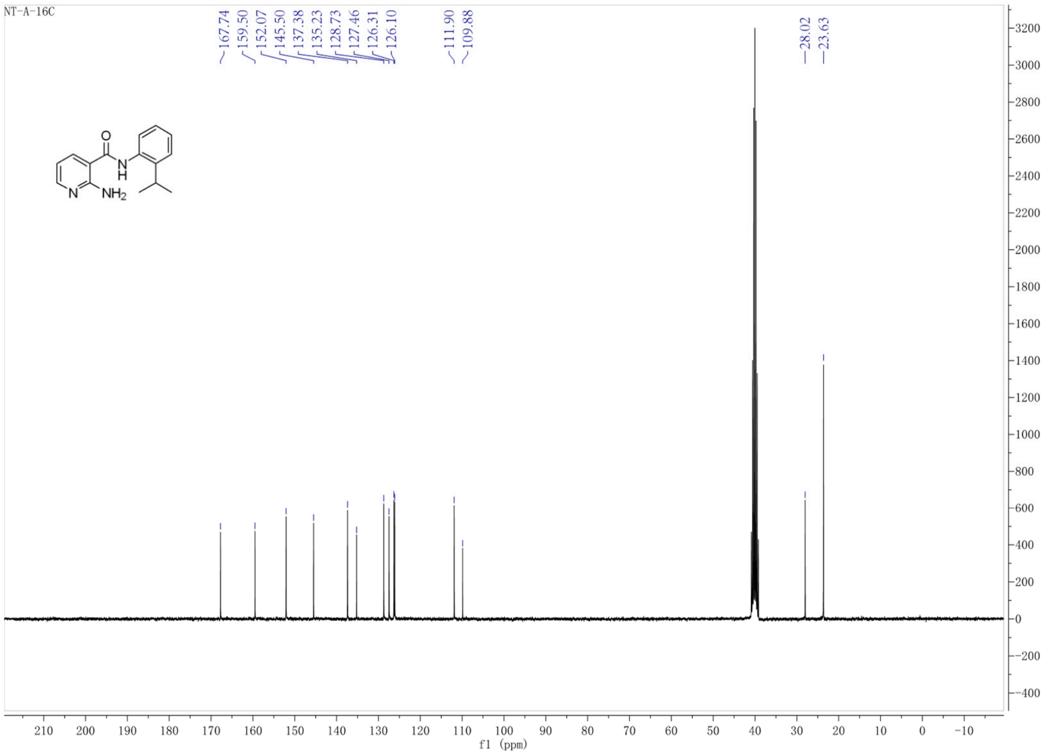

^{13}C NMR spectra of 16i

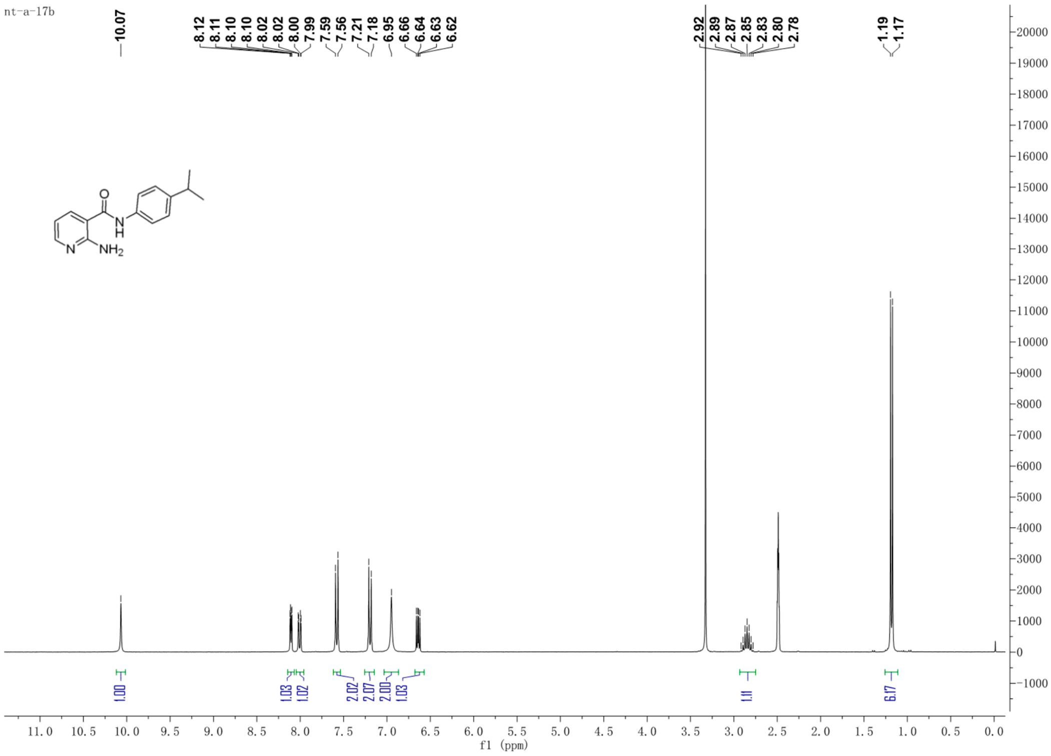

^{1}H NMR spectra of 16j

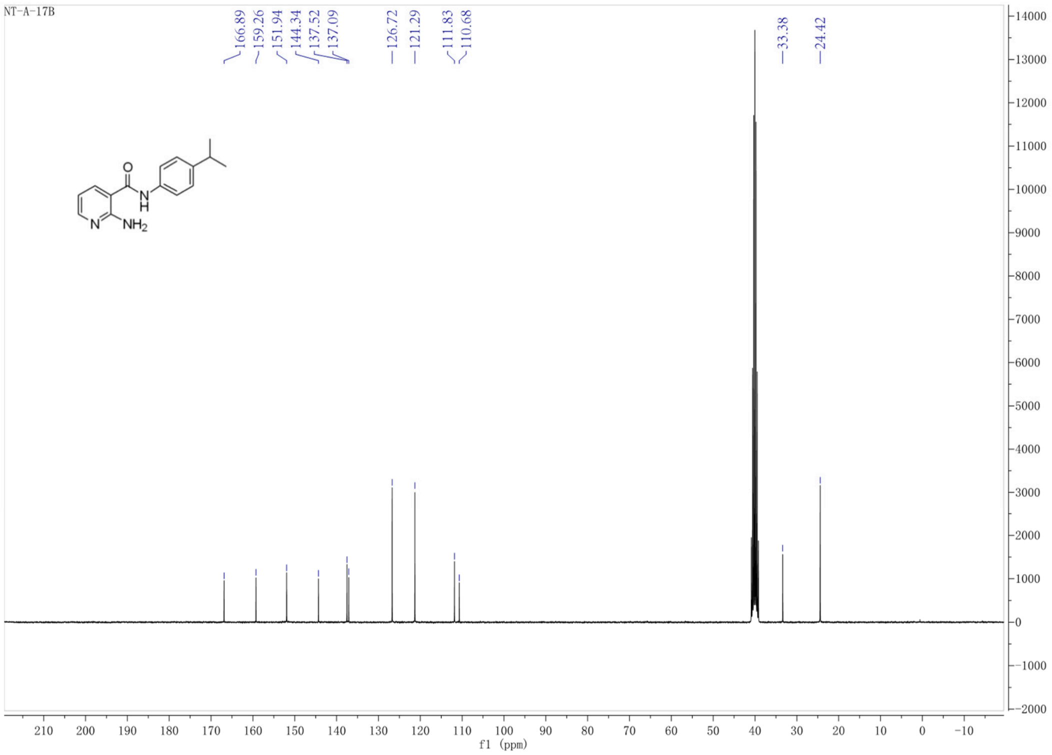

^{13}C NMR spectra of 16j

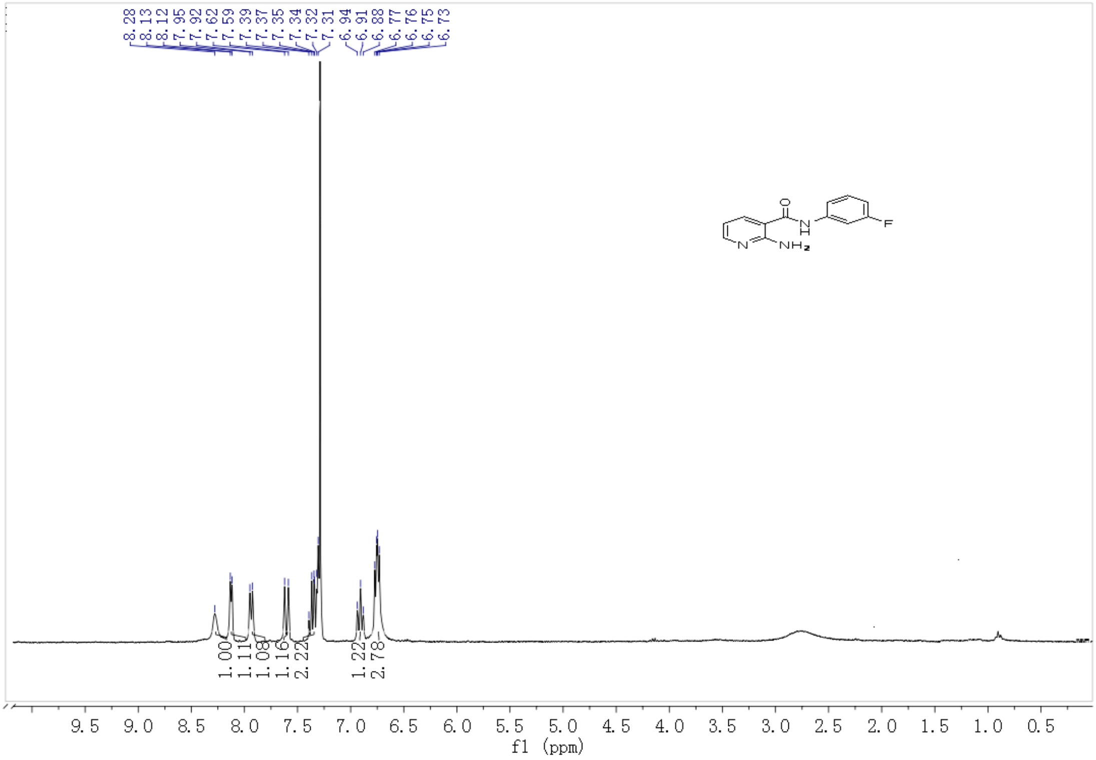

^{1}H NMR spectra of 16k

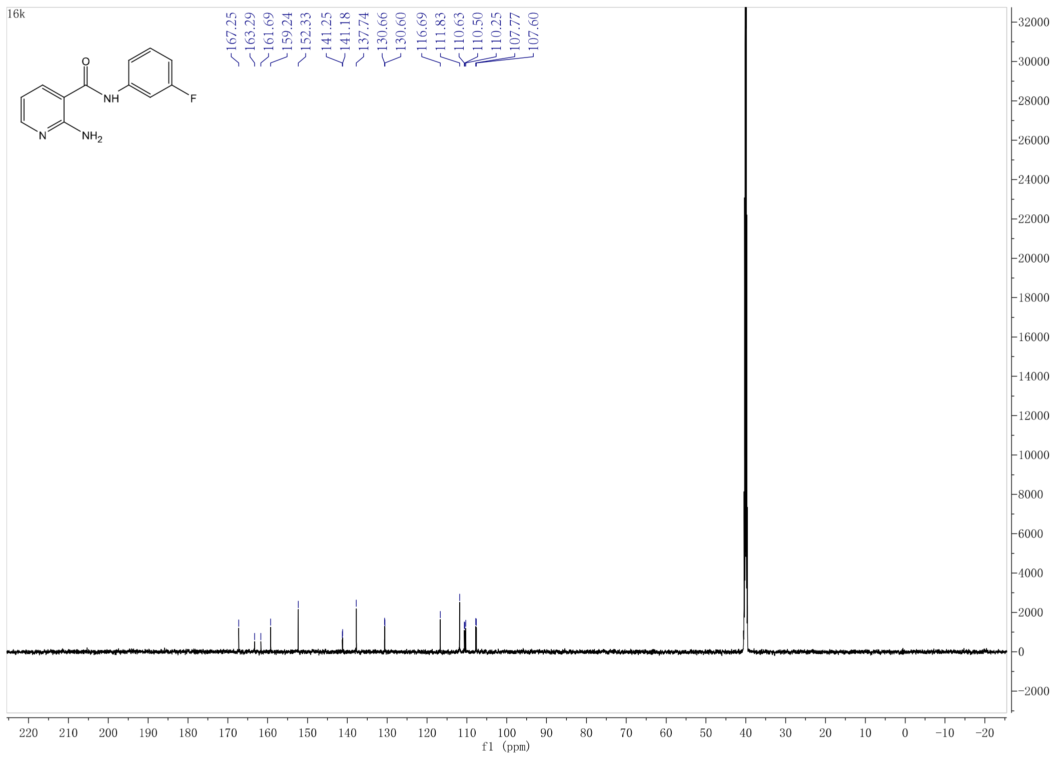

^{13}C NMR spectra of 16k

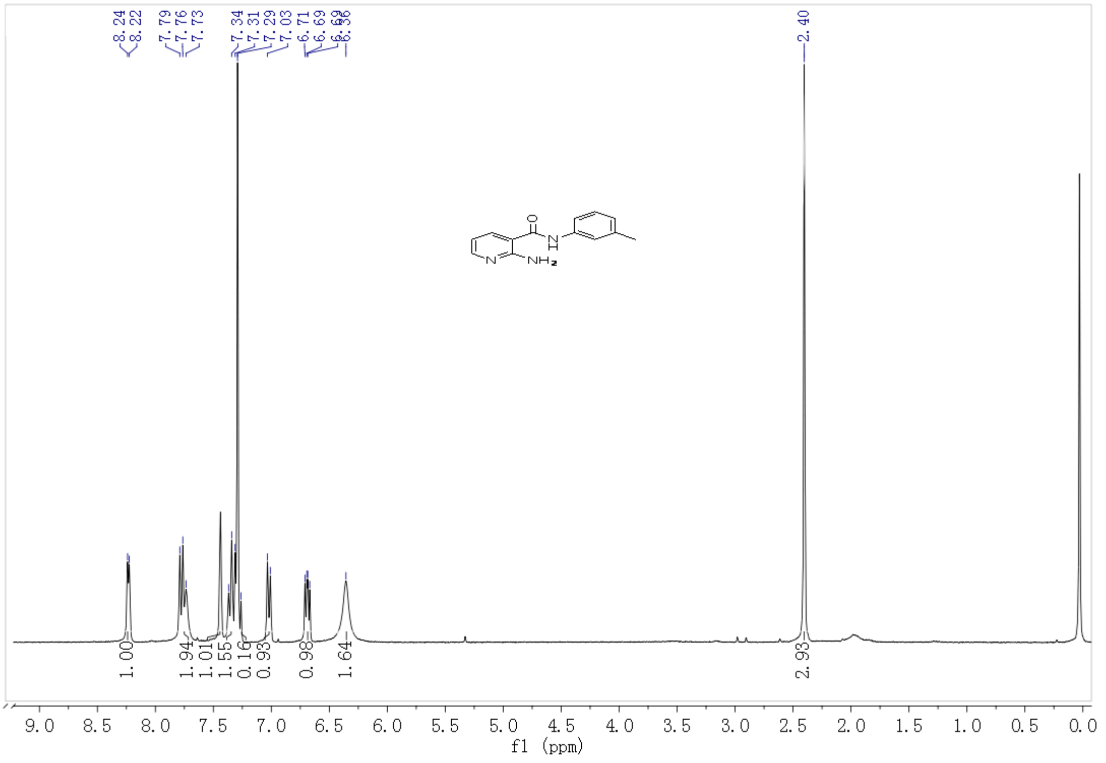

^{1}H NMR spectra of 16l

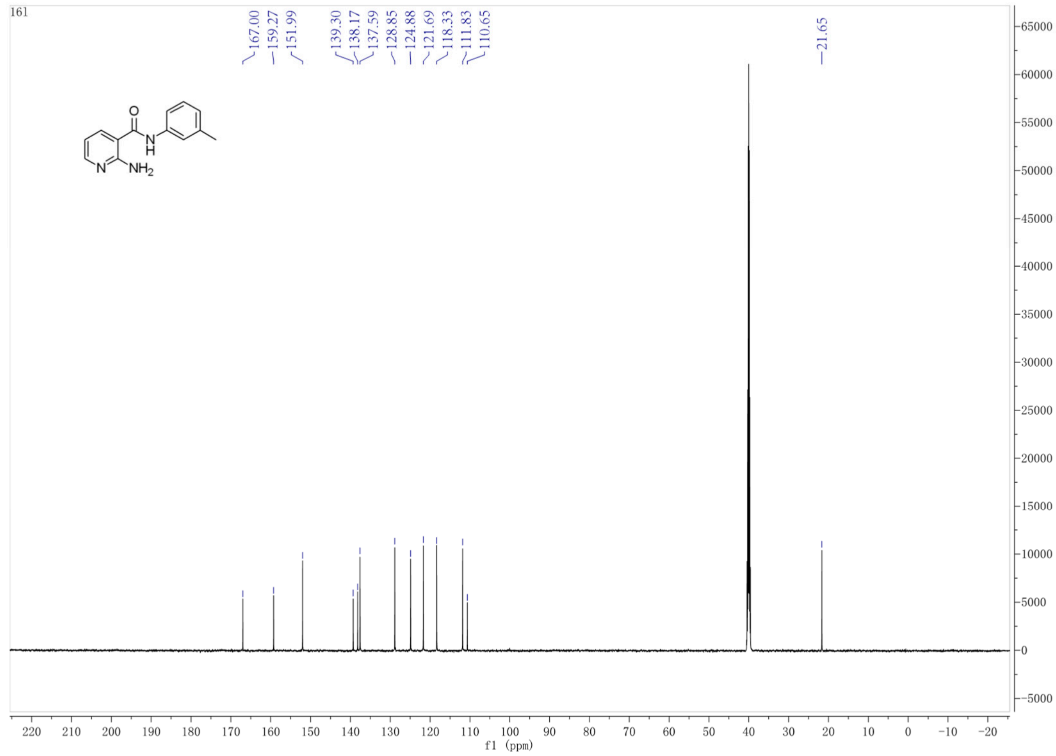

^{13}C NMR spectra of 16l

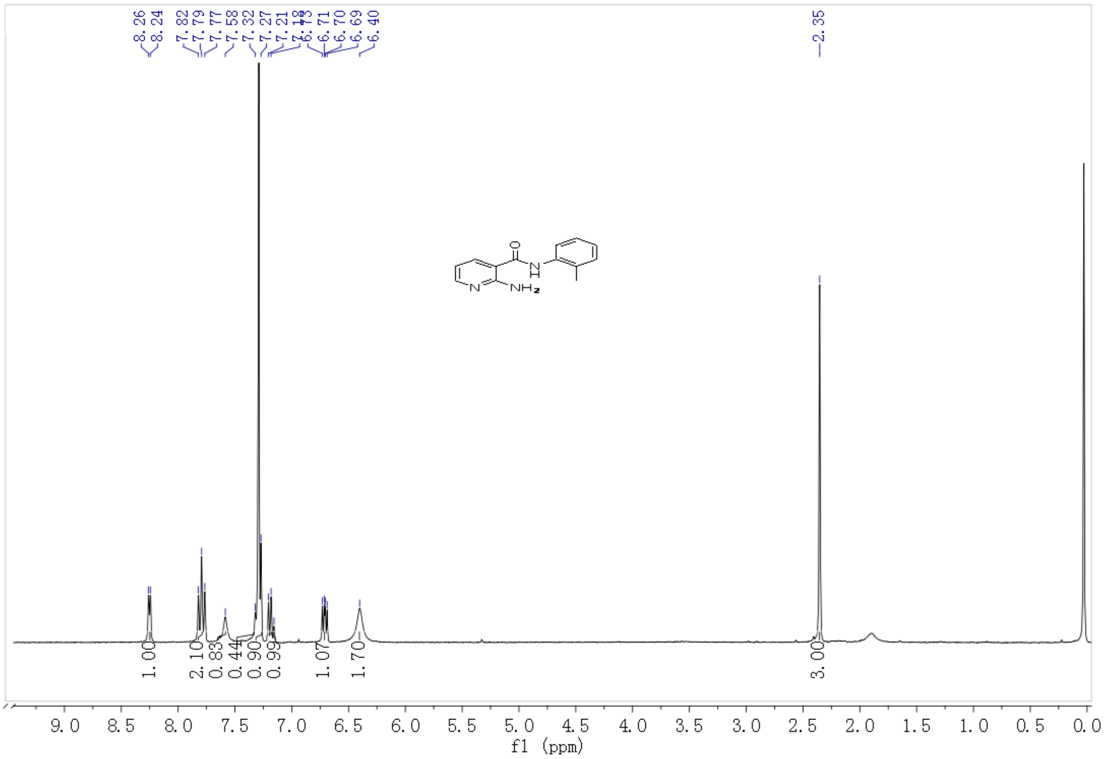

^{1}H NMR spectra of 16m

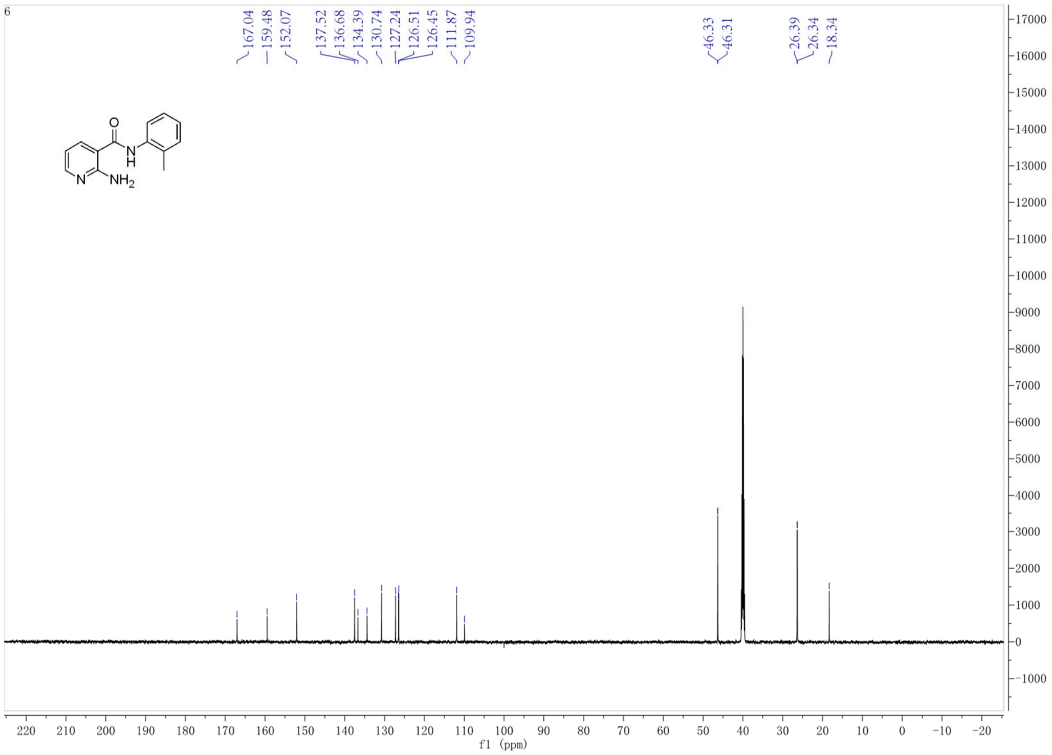

^{13}C NMR spectra of 16m

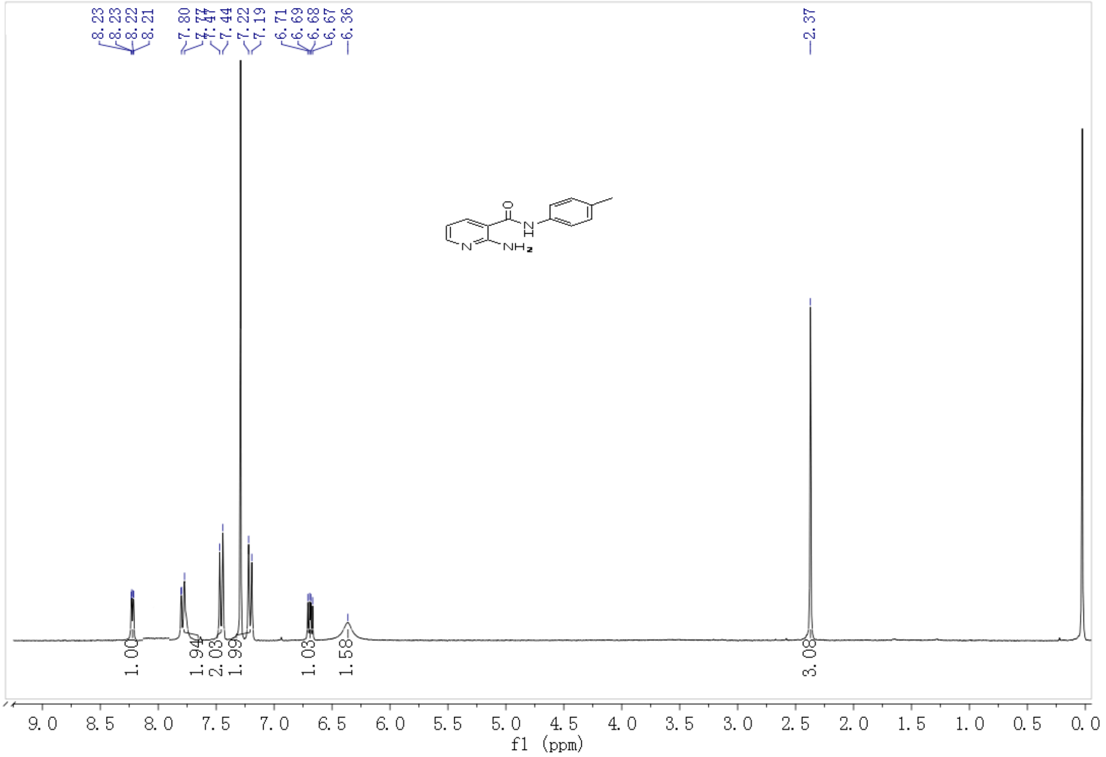

^{1}H NMR spectra of 16n

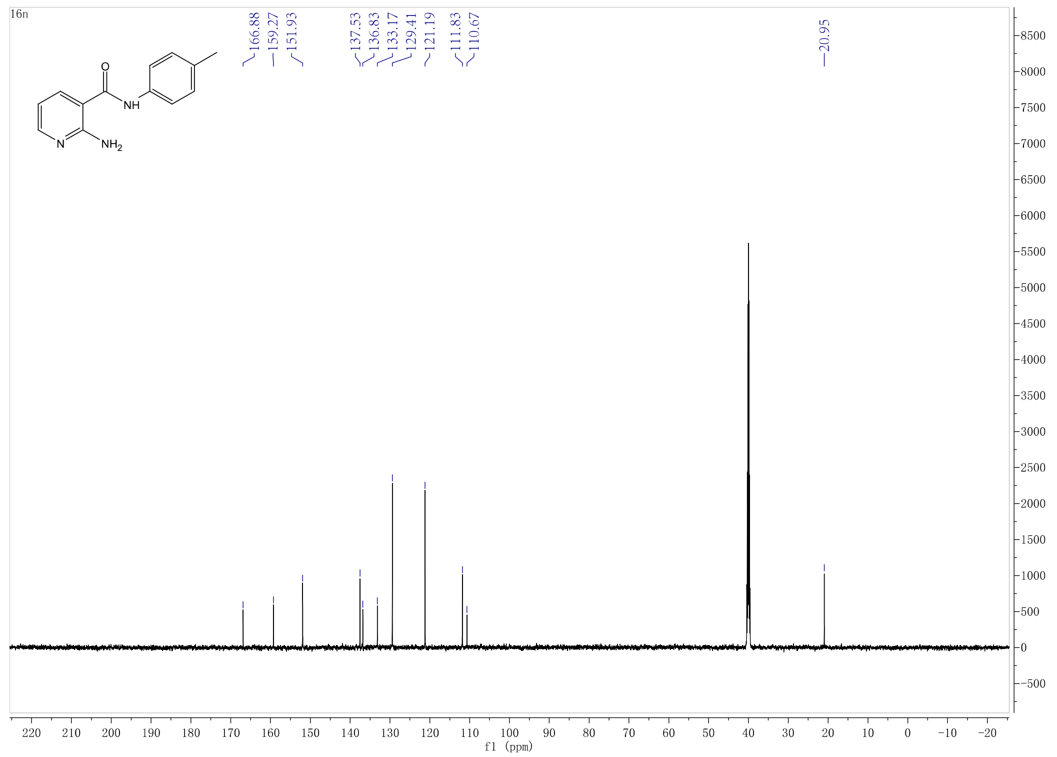

^{13}C NMR spectra of 16n

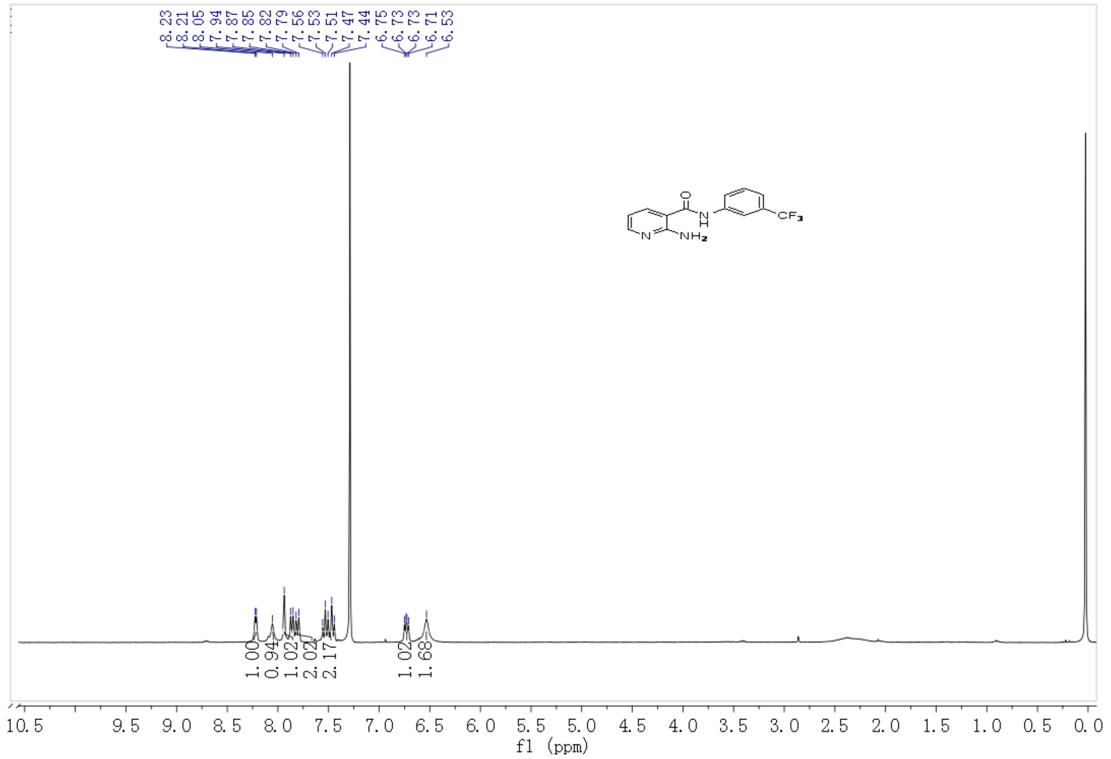

^{1}H NMR spectra of 16o

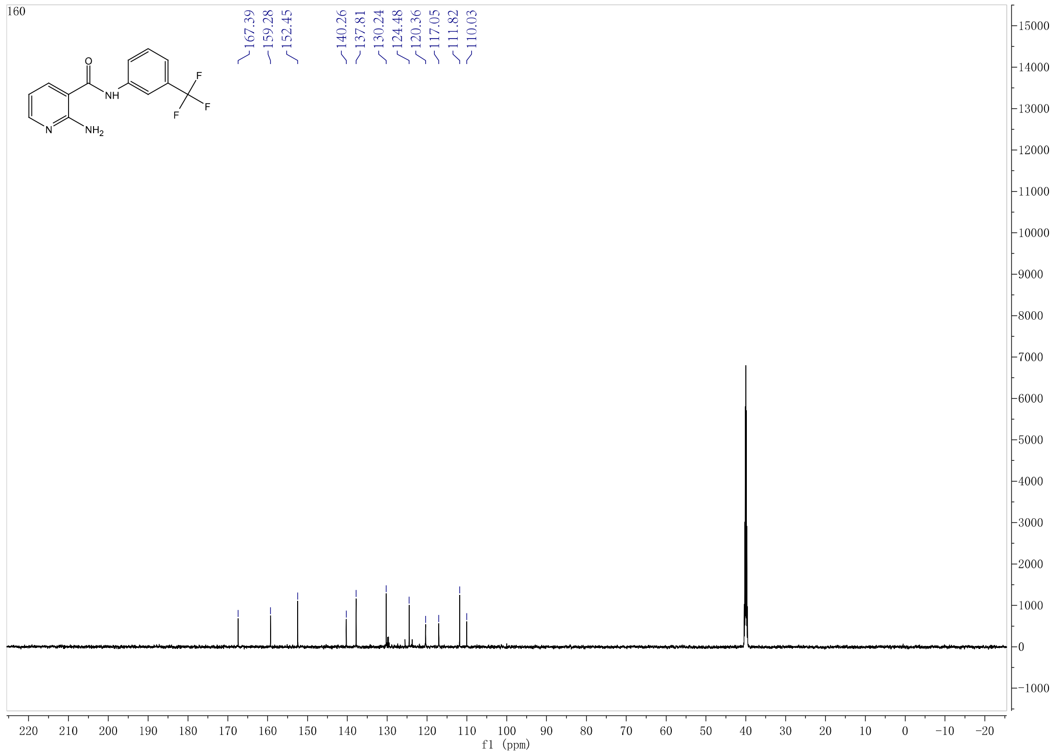

^{13}C NMR spectra of 16o

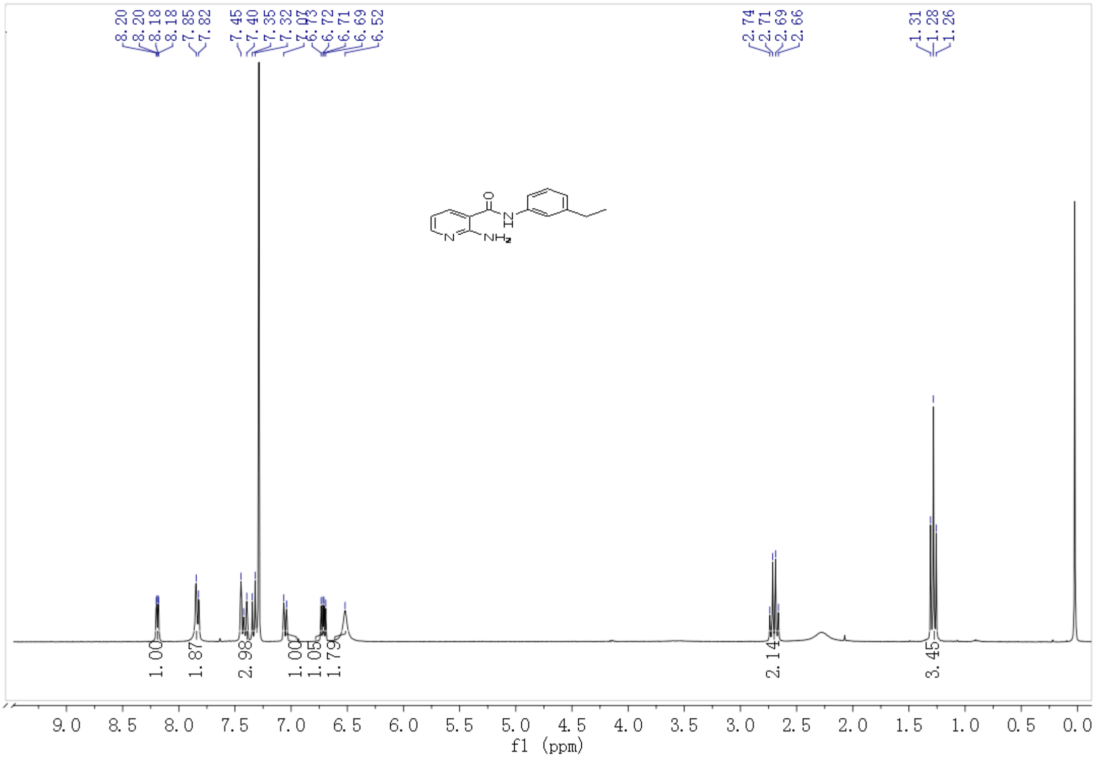

^{1}H NMR spectra of 16p

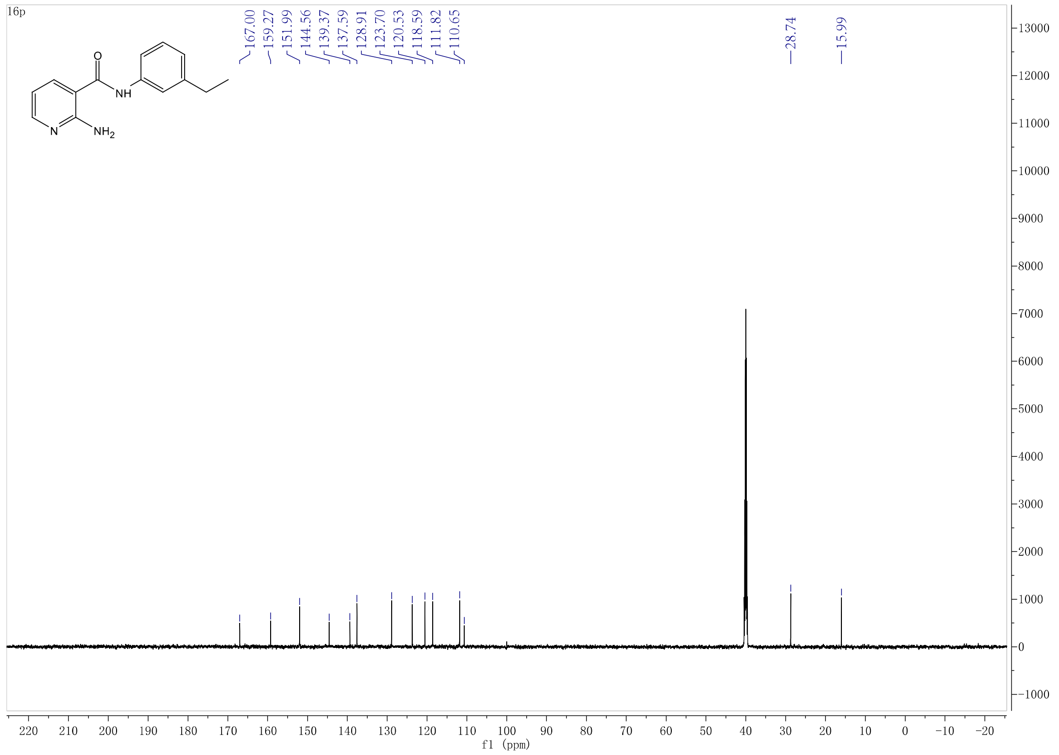

^{13}C NMR spectra of 16p

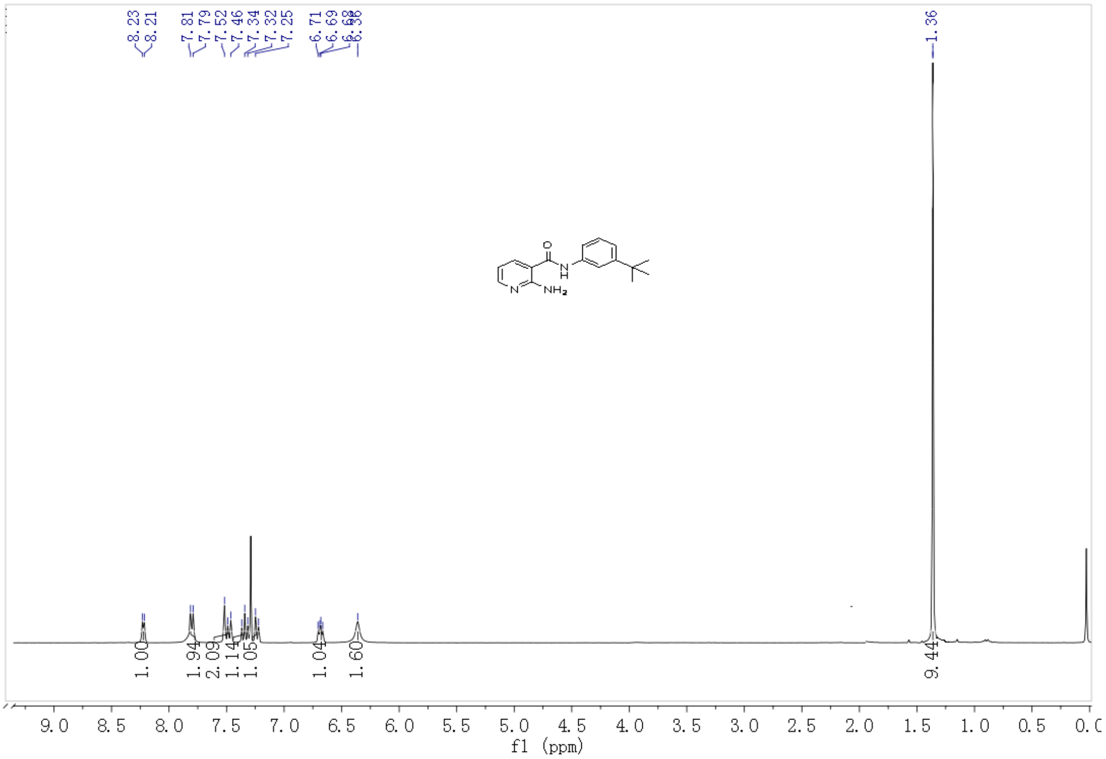

^{1}H NMR spectra of 16q

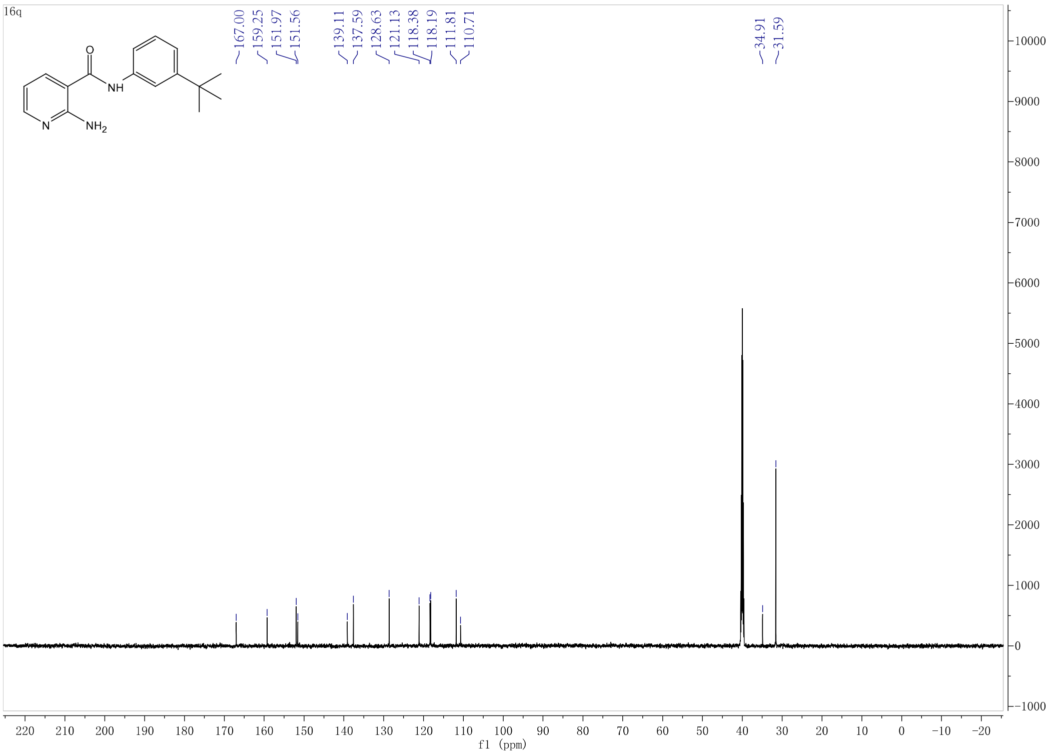

^{13}C NMR spectra of 16q

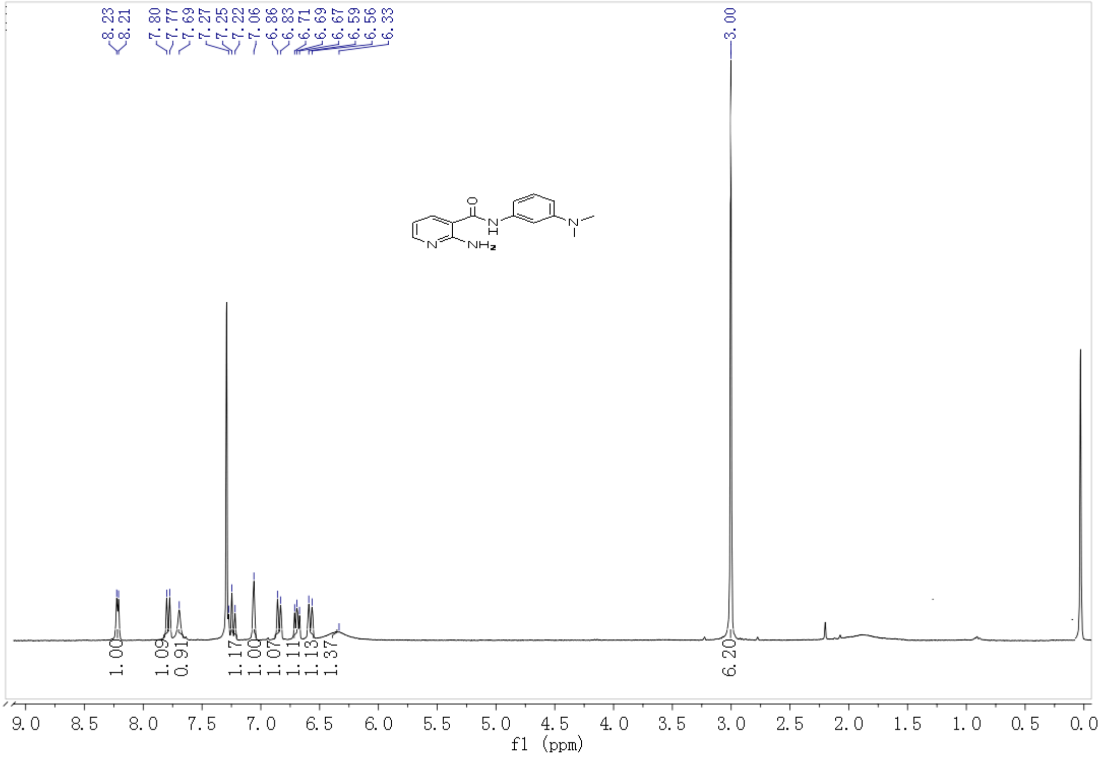

^{1}H NMR spectra of 16r

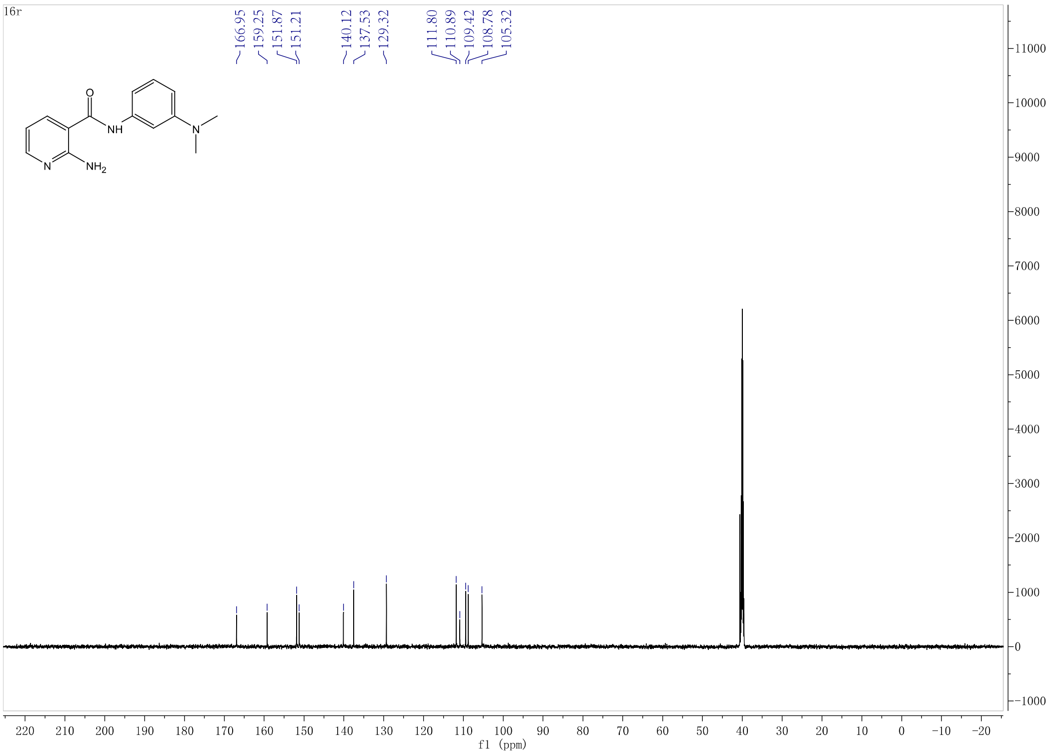

^{13}C NMR spectra of 16r

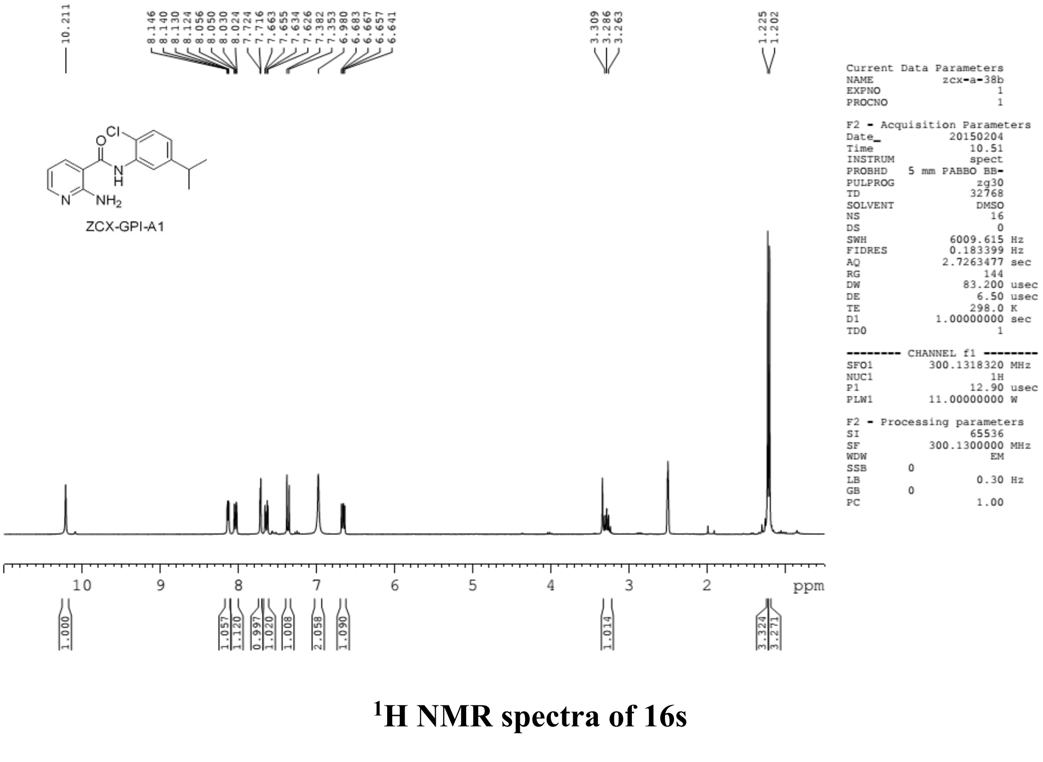

^{1}H NMR spectra of 16s

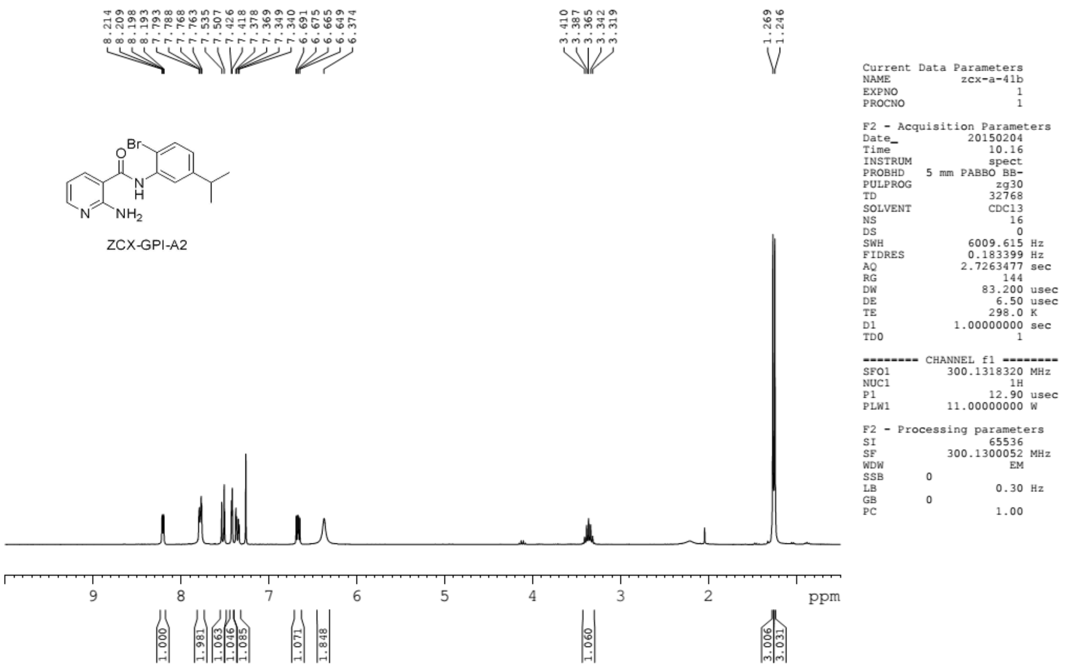

^{1}H NMR spectra of 16t

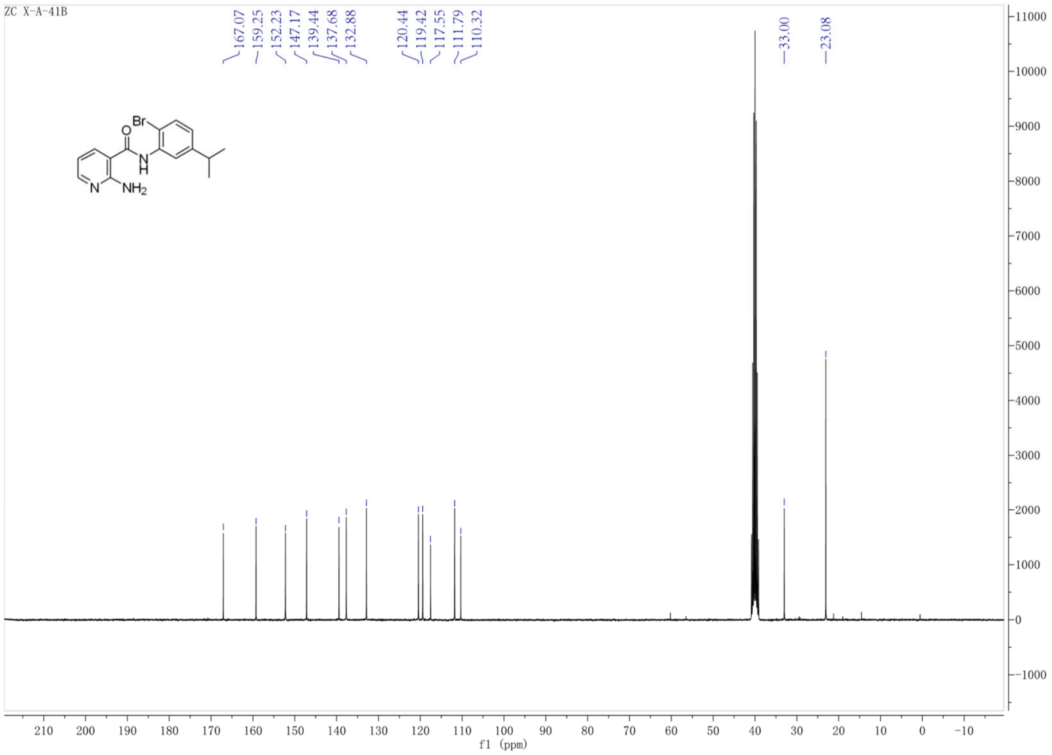

^{13}C NMR spectra of 16t

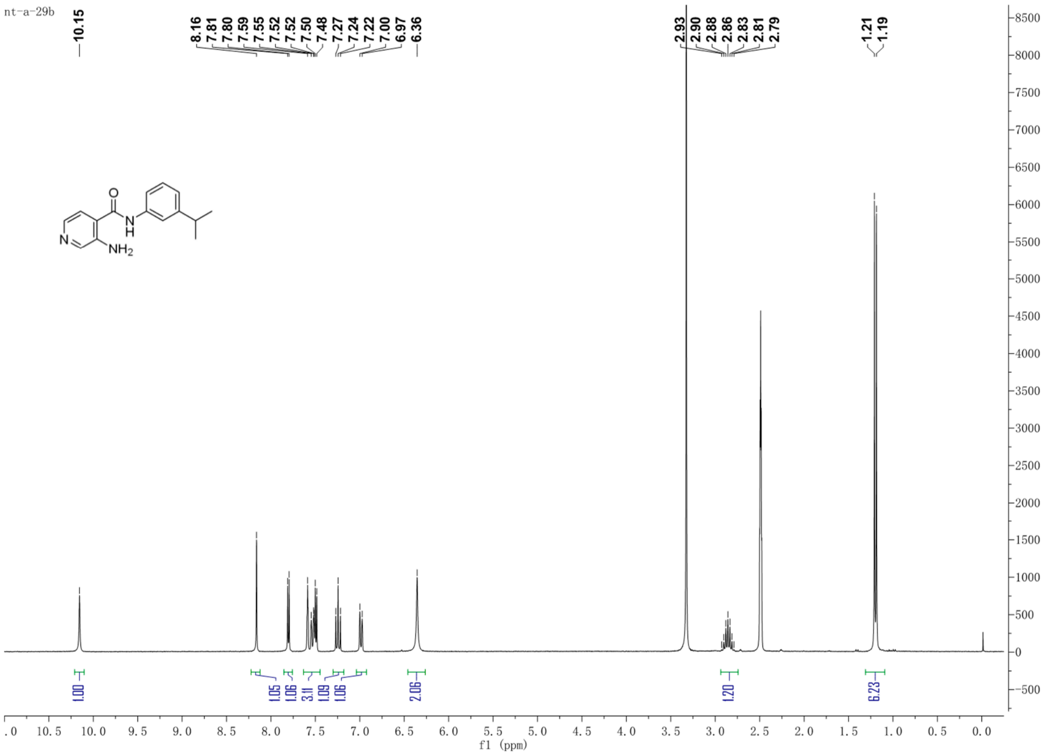

^{1}H NMR spectra of 16u

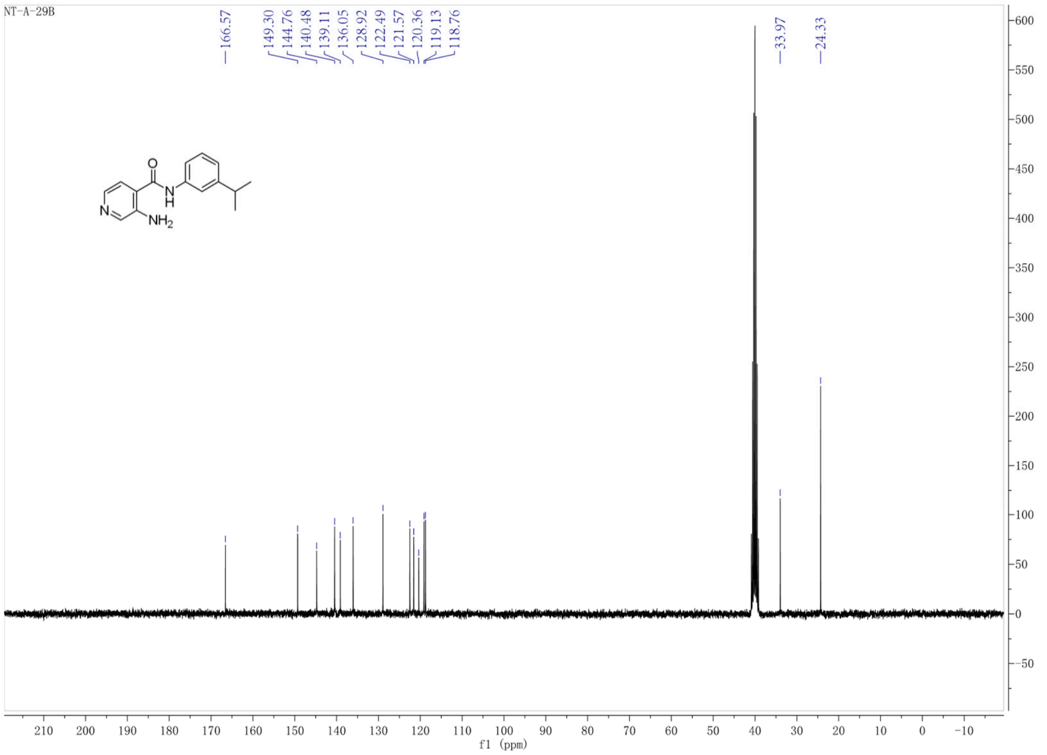

^{13}C NMR spectra of 16u

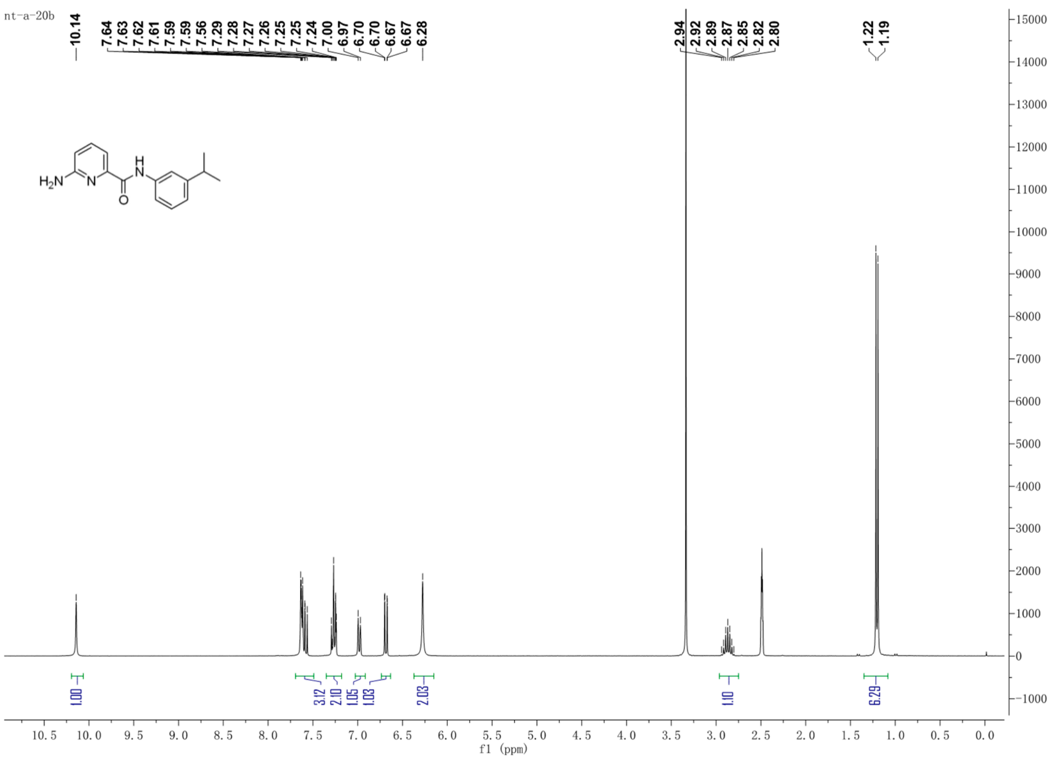

^{1}H NMR spectra of 16v

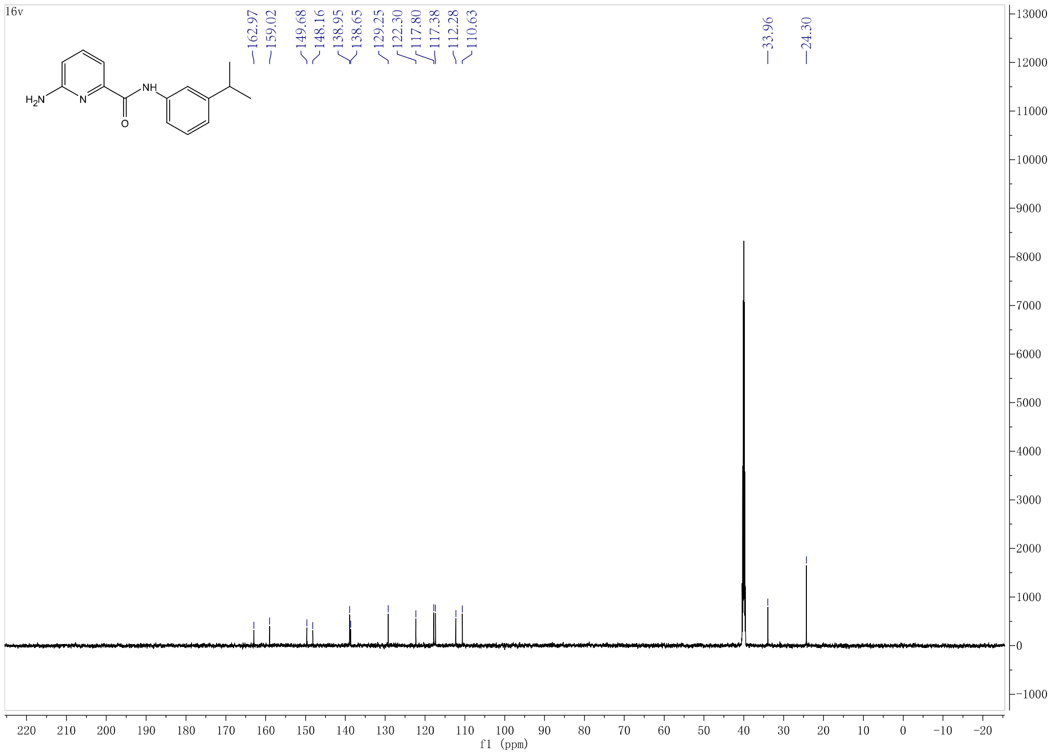

^{13}C NMR spectra of 16v

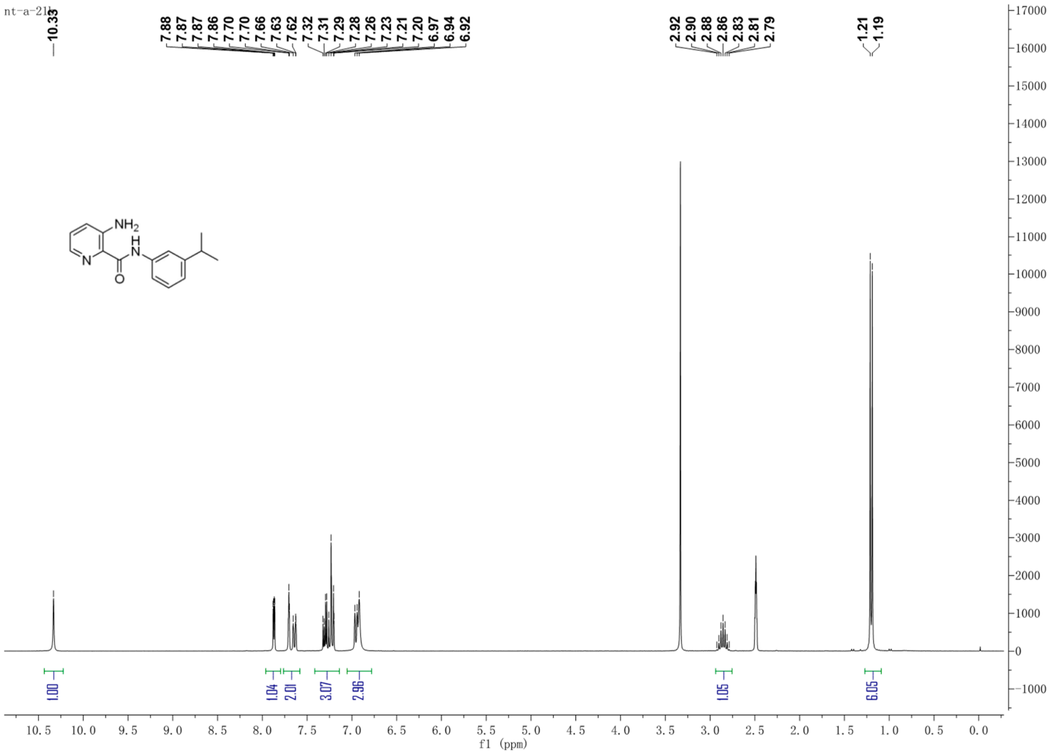

^{1}H NMR spectra of 16w

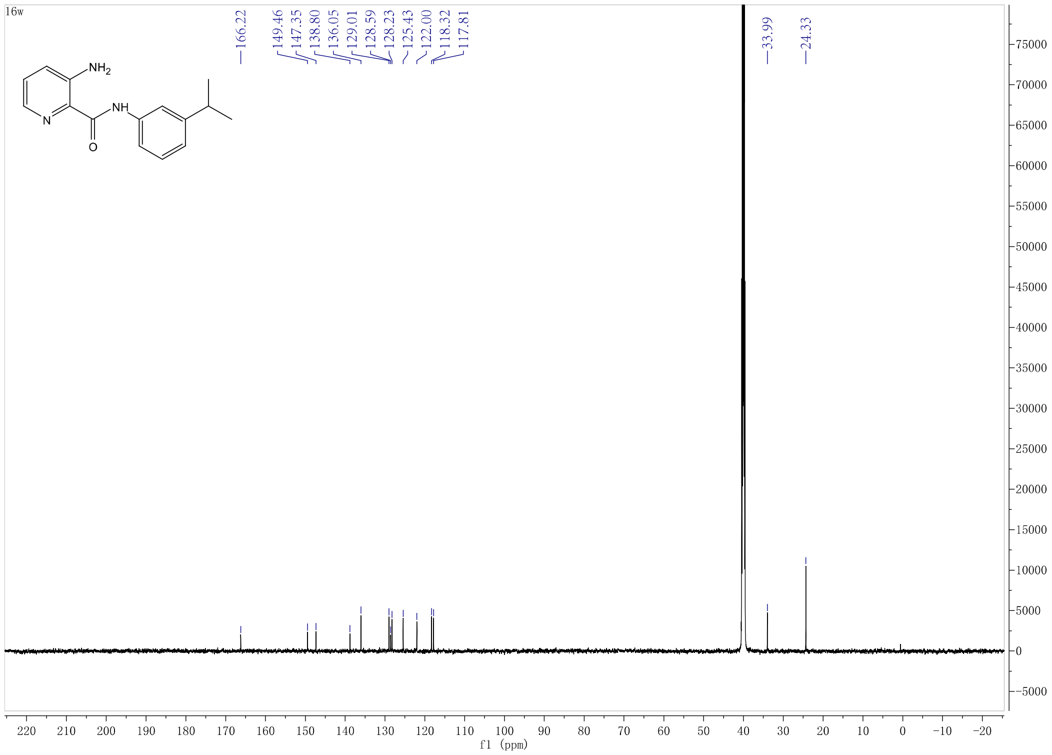

^{13}C NMR spectra of 16w

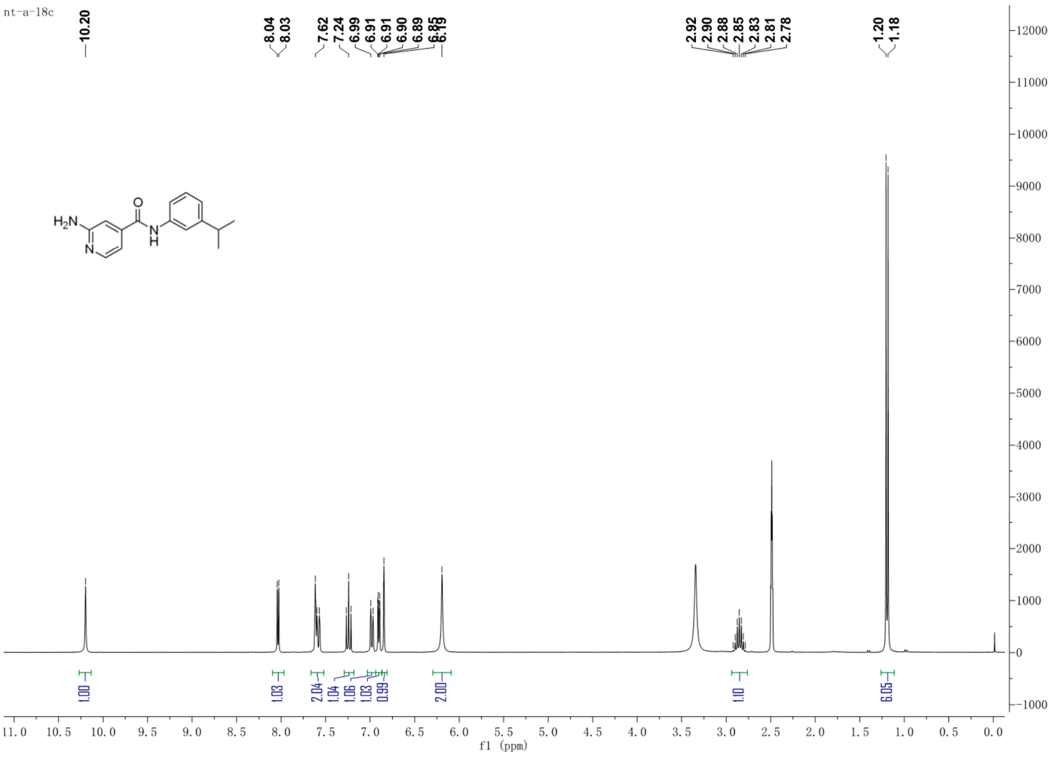

^{1}H NMR spectra of 16x

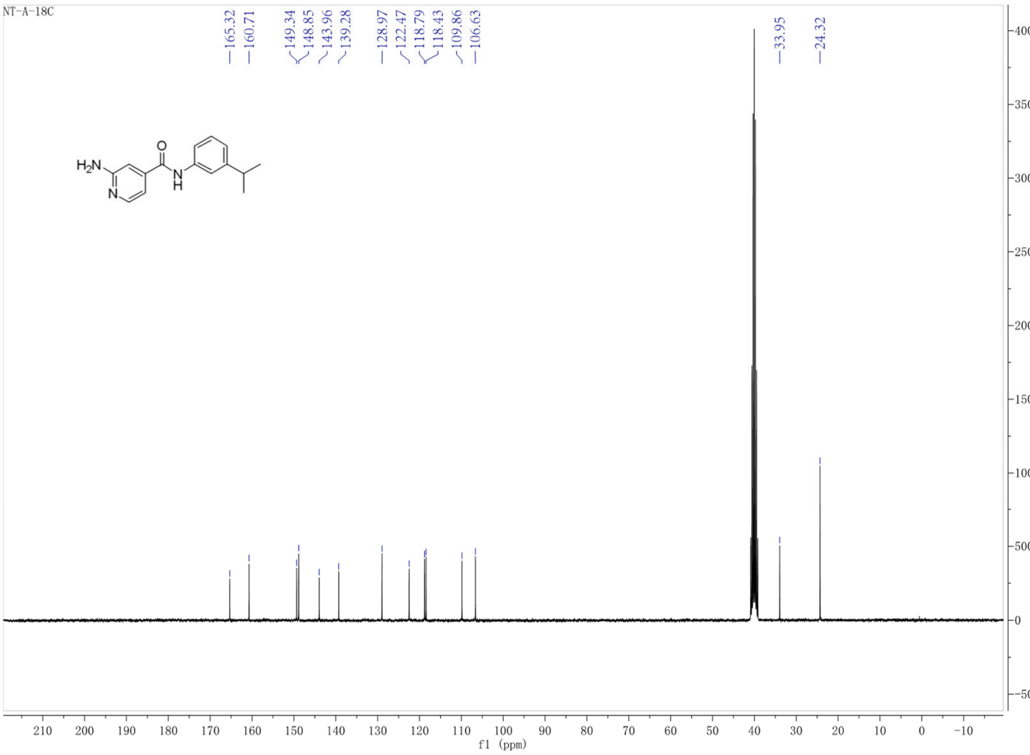

^{13}C NMR spectra of 16x

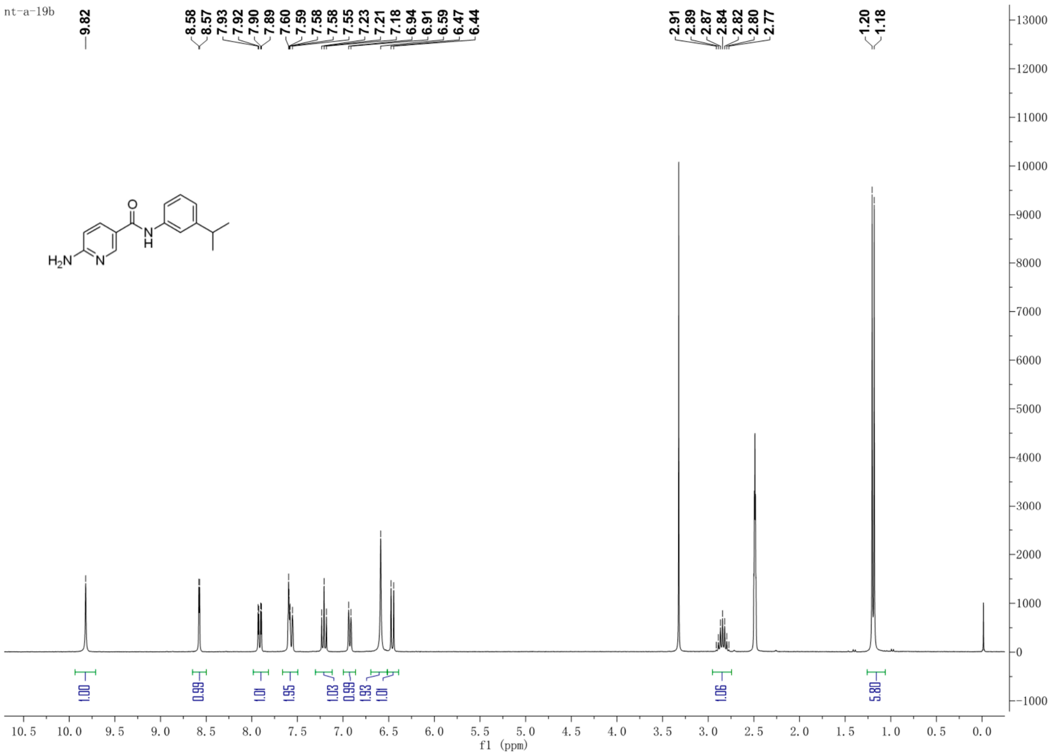

^{1}H NMR spectra of 16y

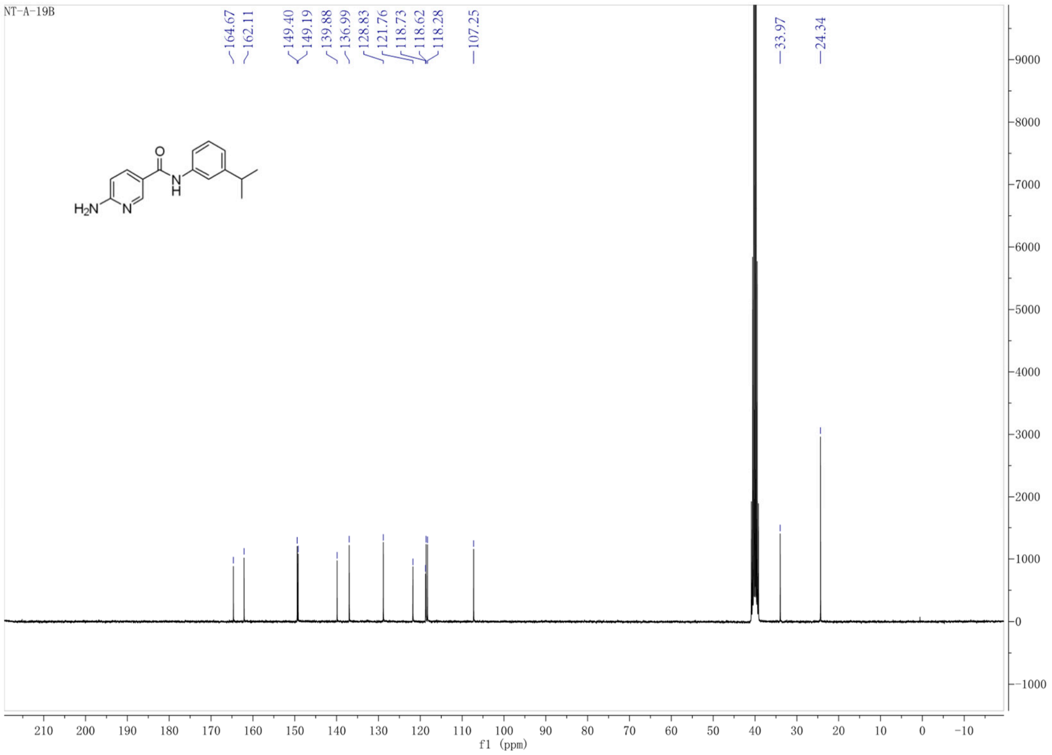

^{13}C NMR spectra of 16y

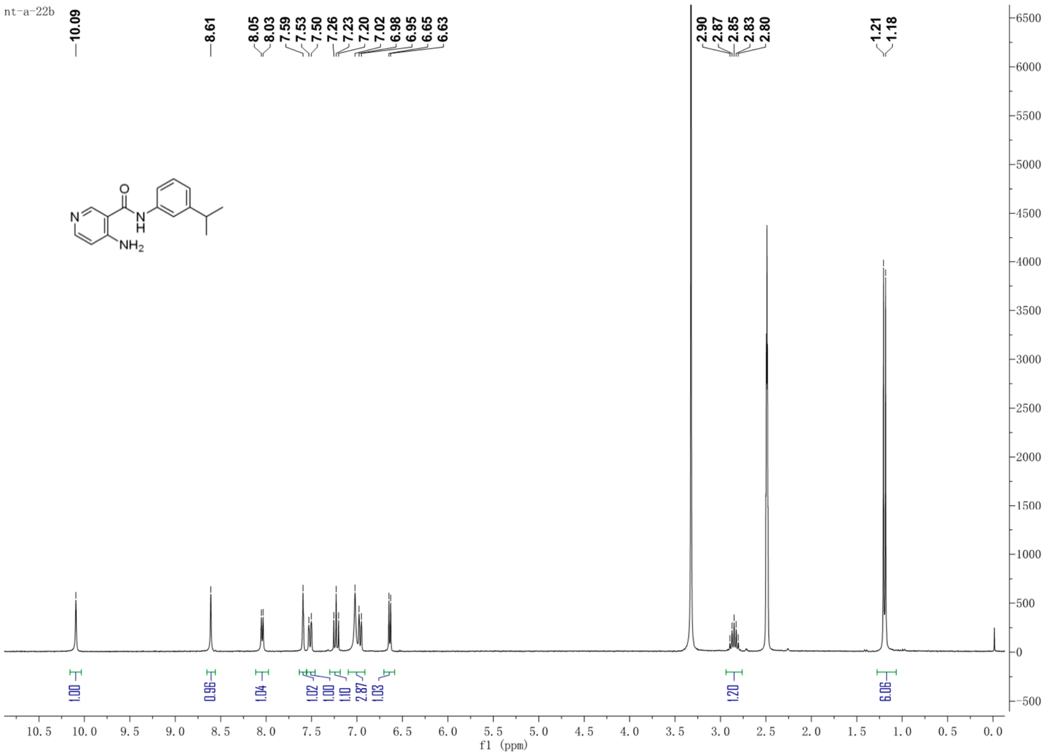

^{1}H NMR spectra of 16z

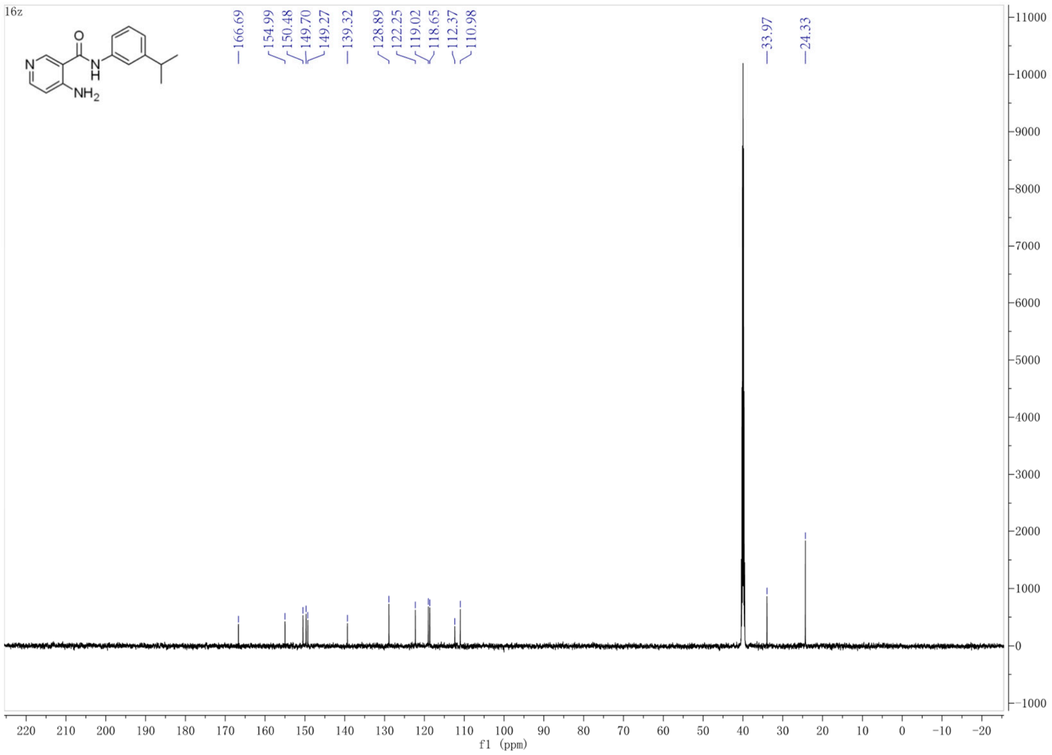

^{13}C NMR spectra of 16z

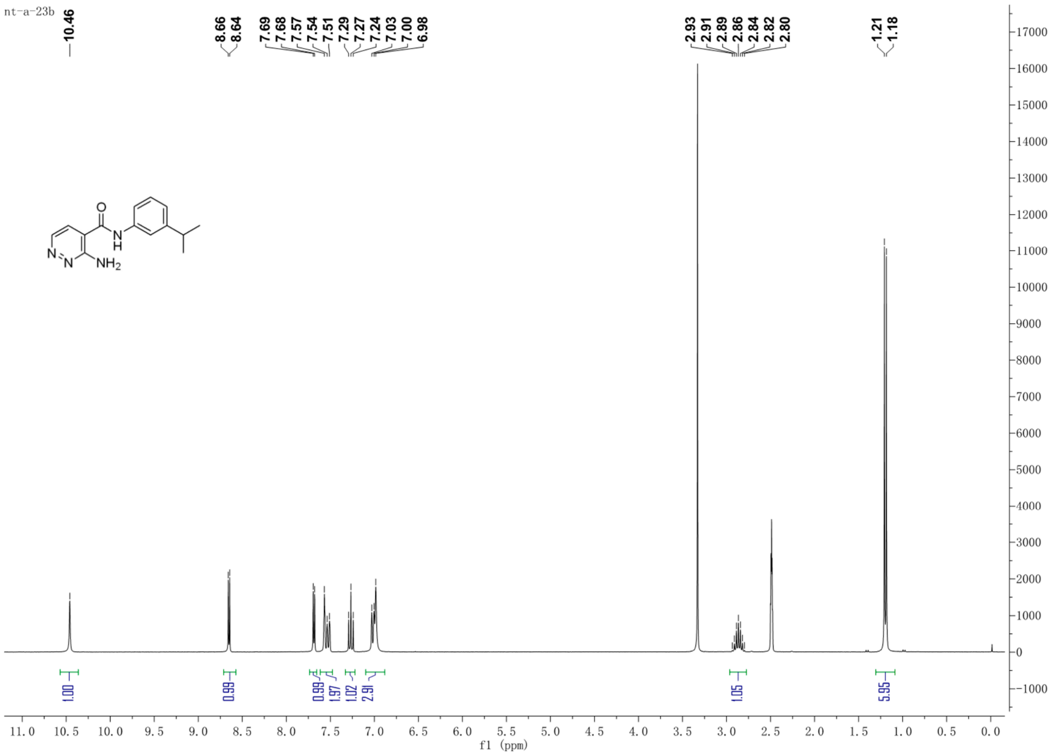

^{1}H NMR spectra of 17

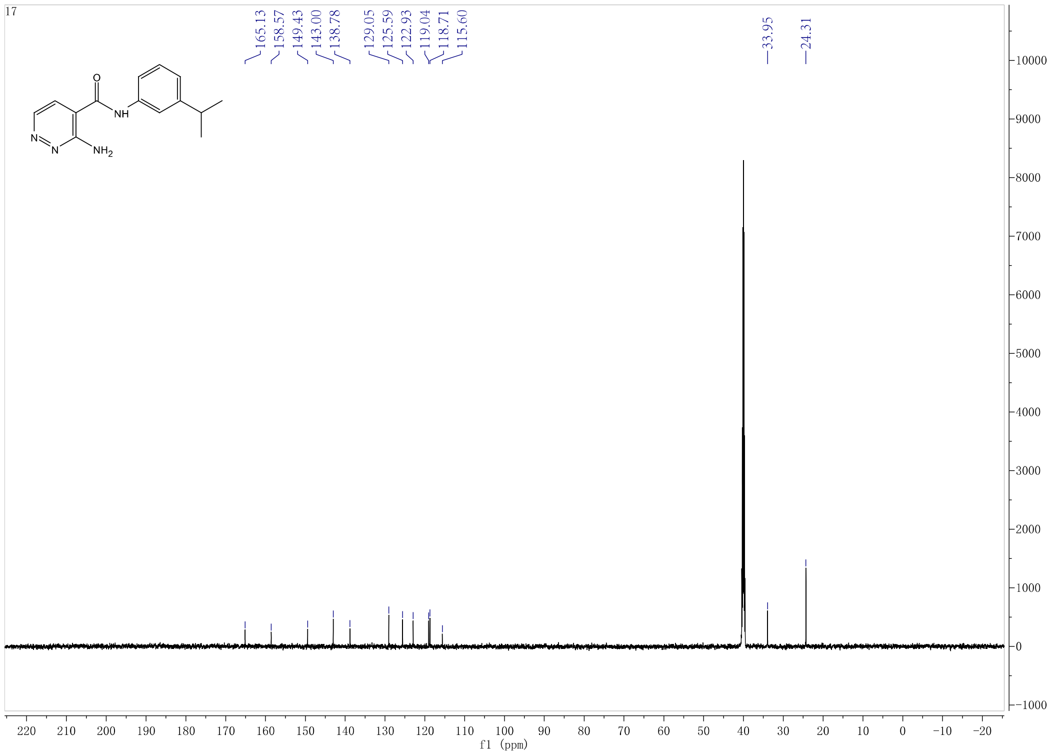

^{13}C NMR spectra of 17

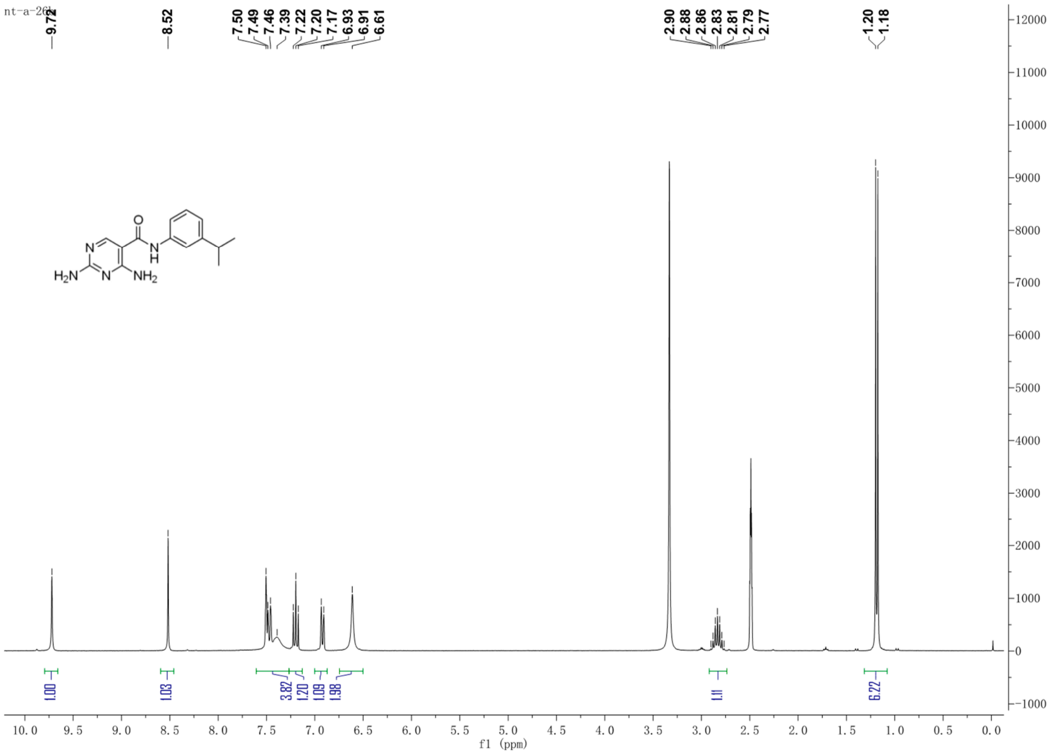

^{1}H NMR spectra of 18a

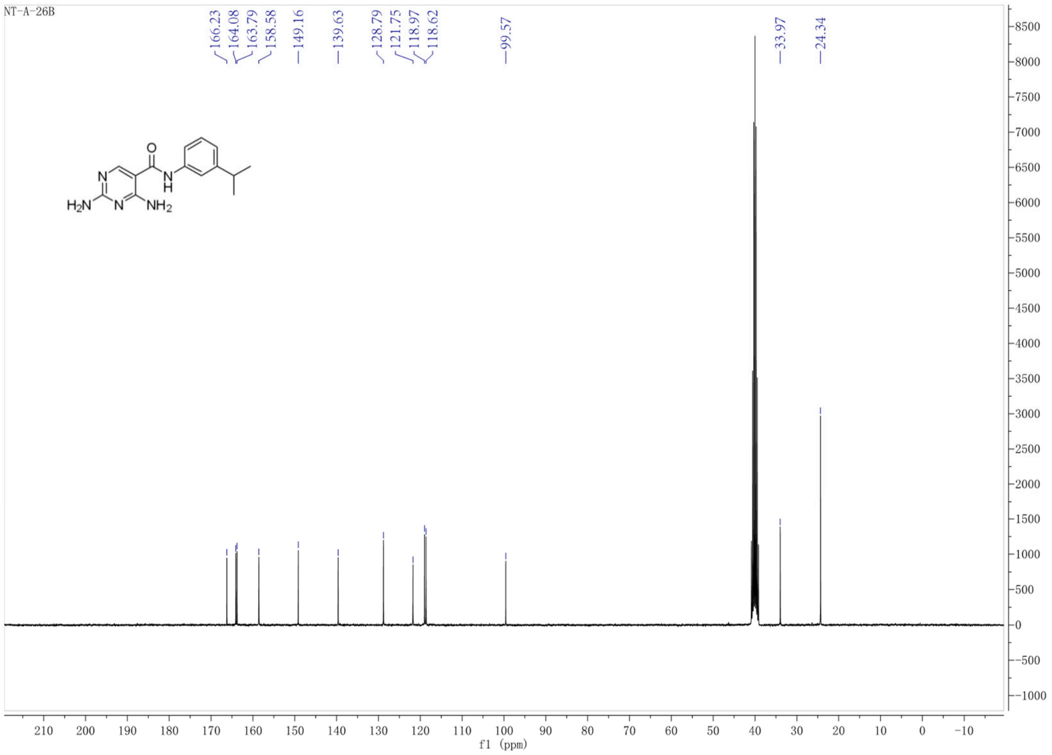

^{13}C NMR spectra of 18a

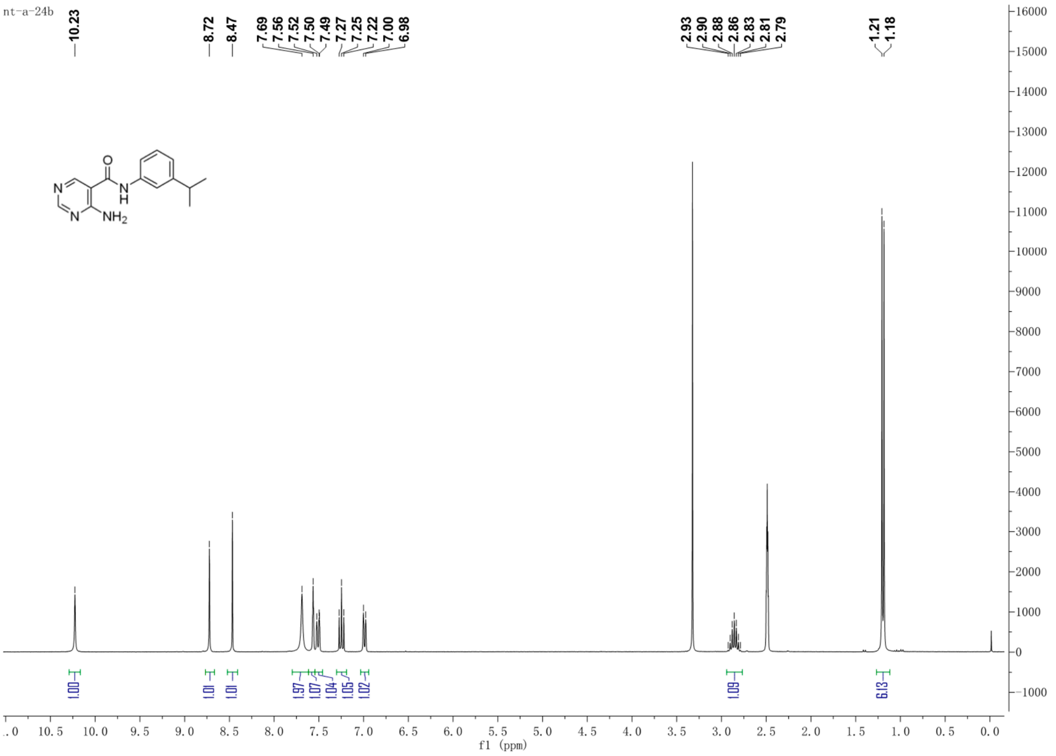

^{1}H NMR spectra of 18b

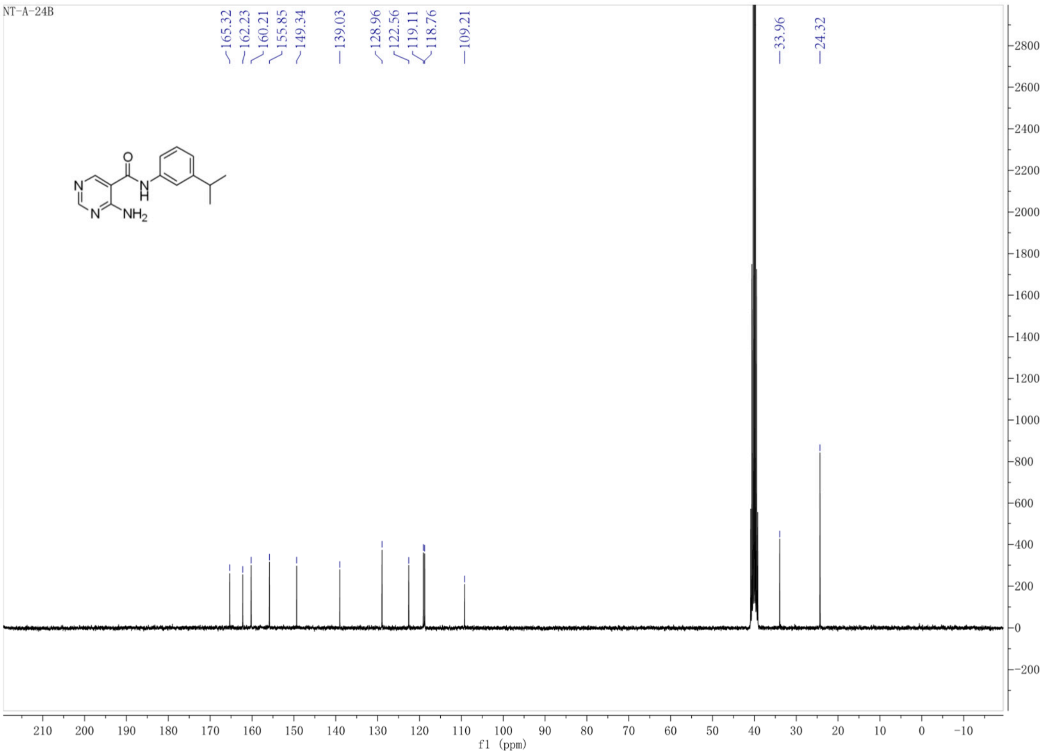

^{13}C NMR spectra of 18b

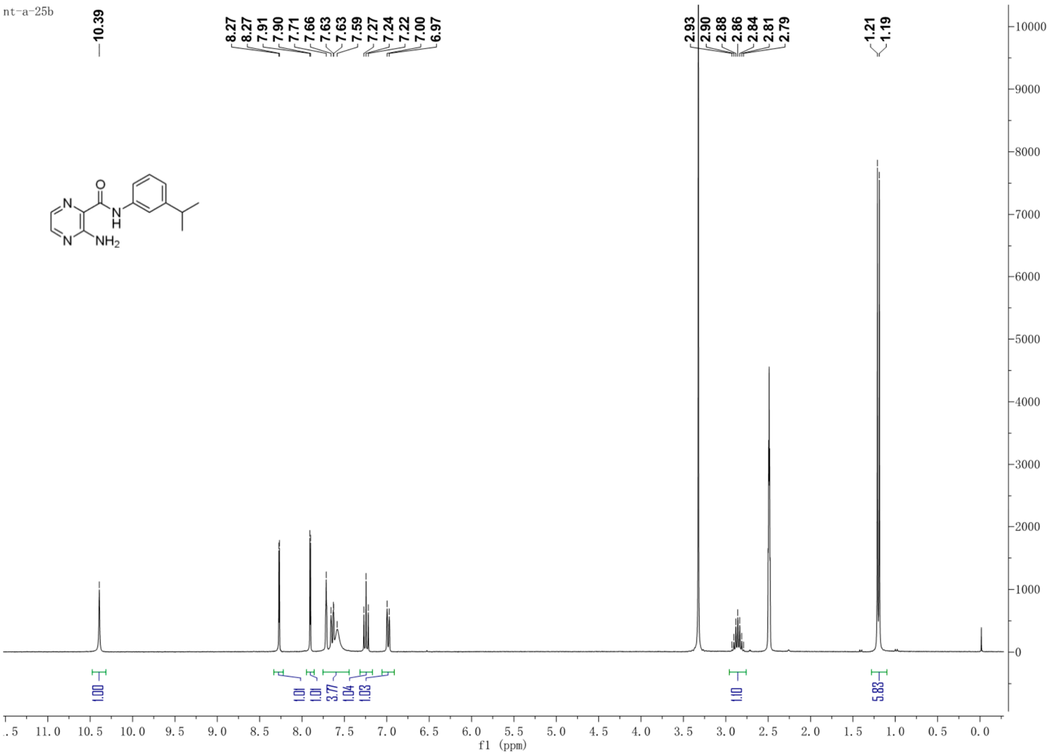

^1H NMR spectra of 19

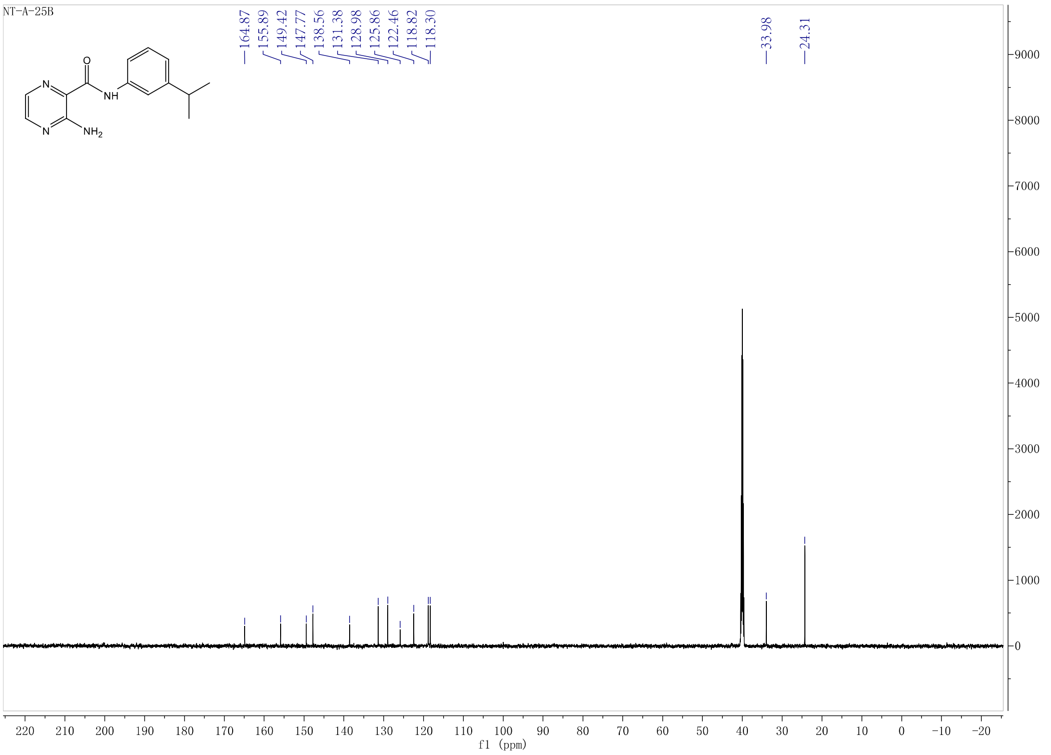

^{13}C NMR spectra of 19

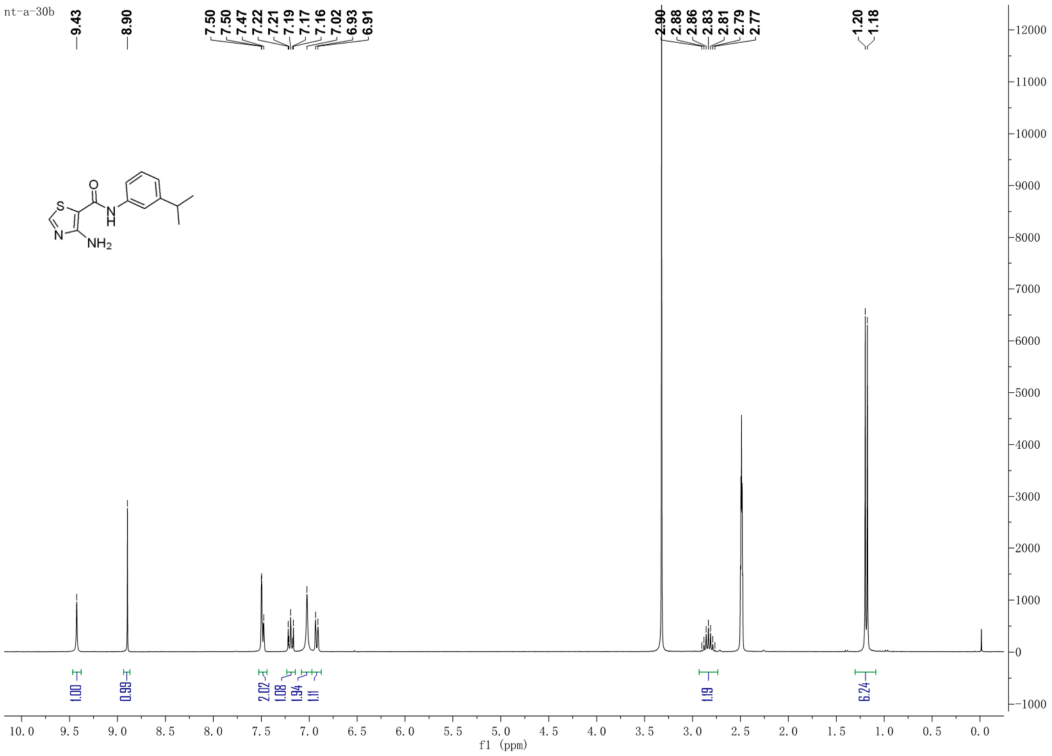

^{1}H NMR spectra of 20

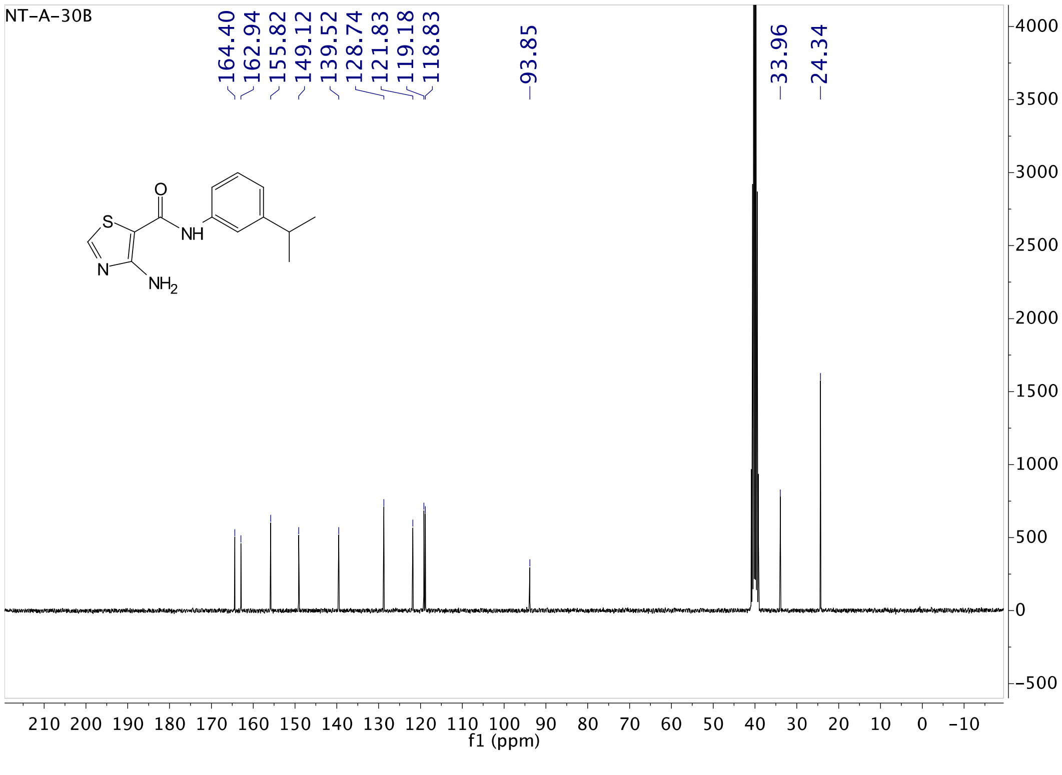

^{13}C NMR spectra of 20

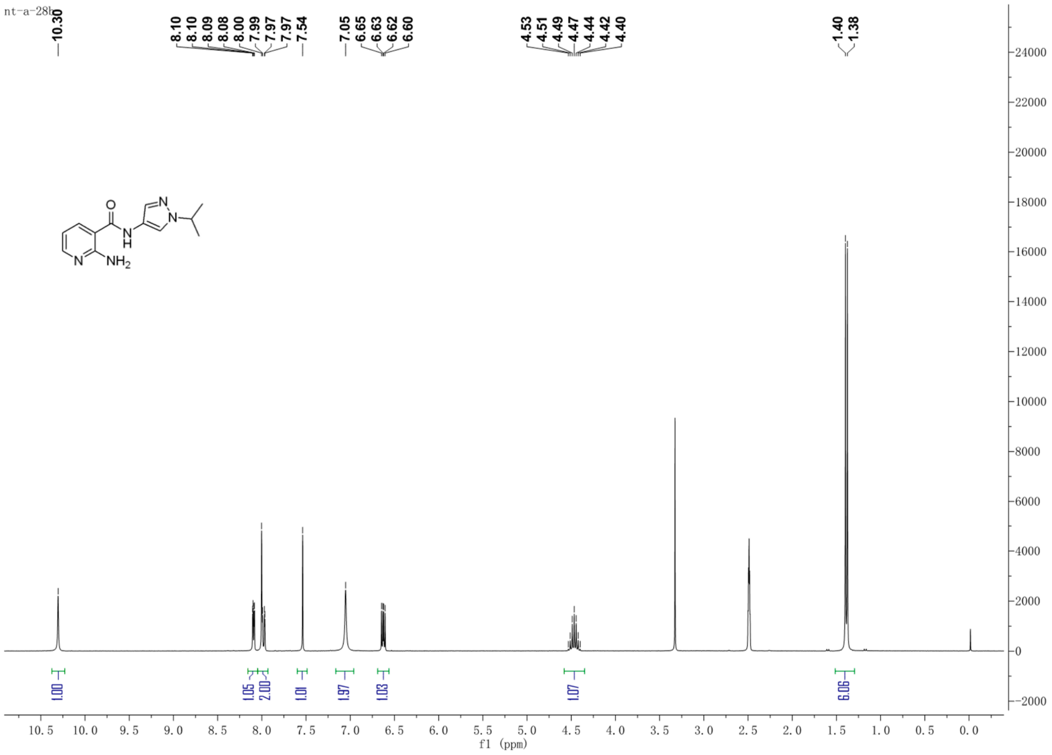

^{1}H NMR spectra of 21

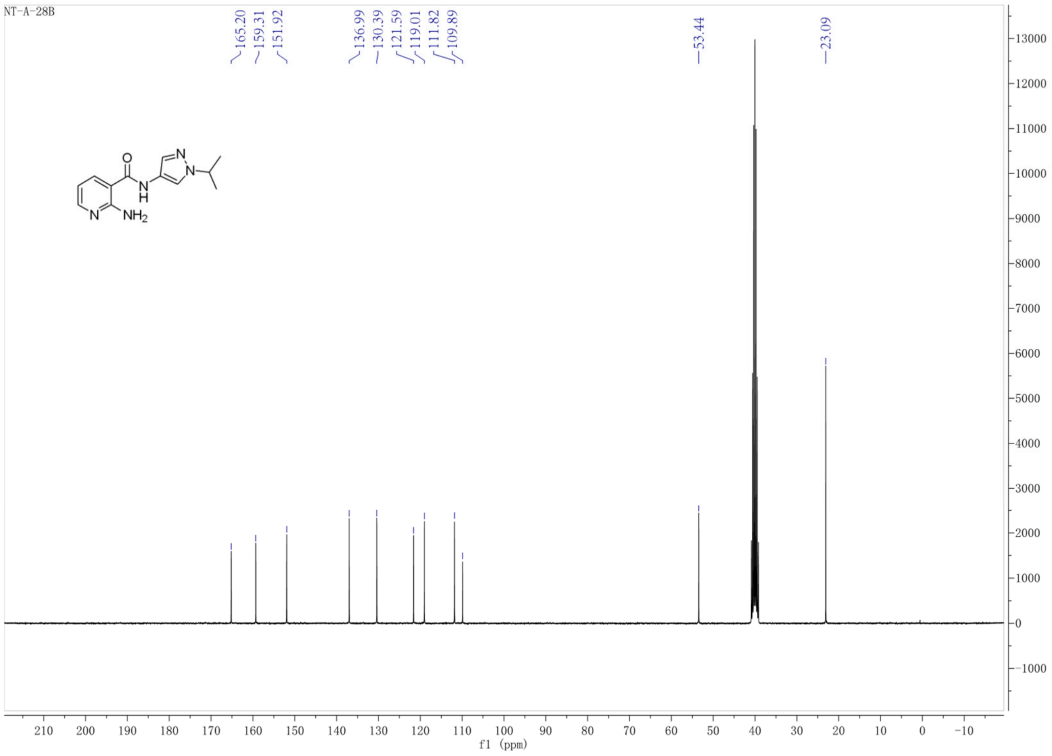

^{13}C NMR spectra of 21

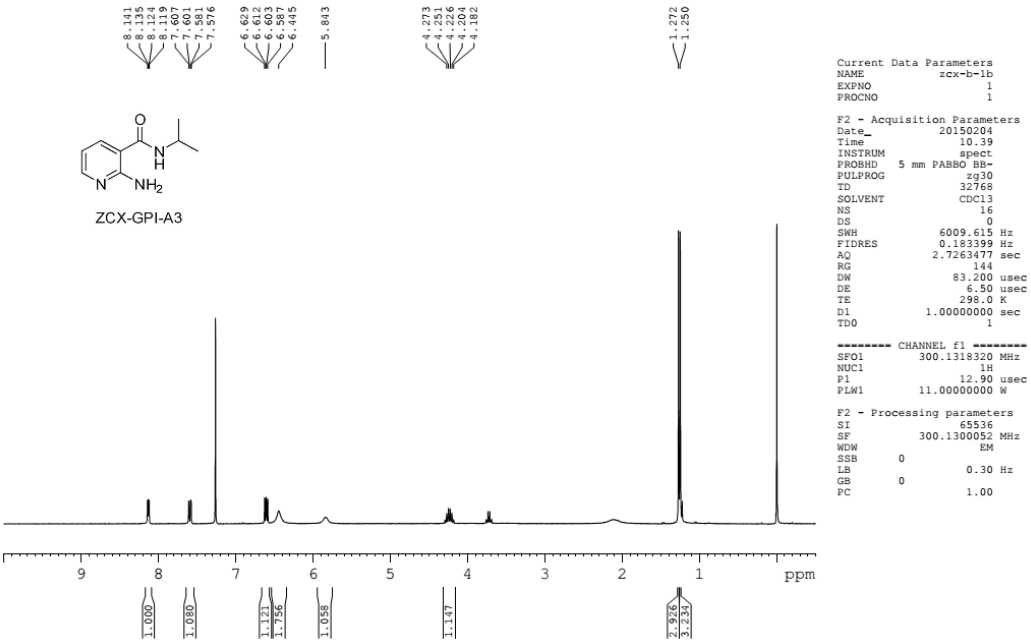

^{1}H NMR spectra of 22a

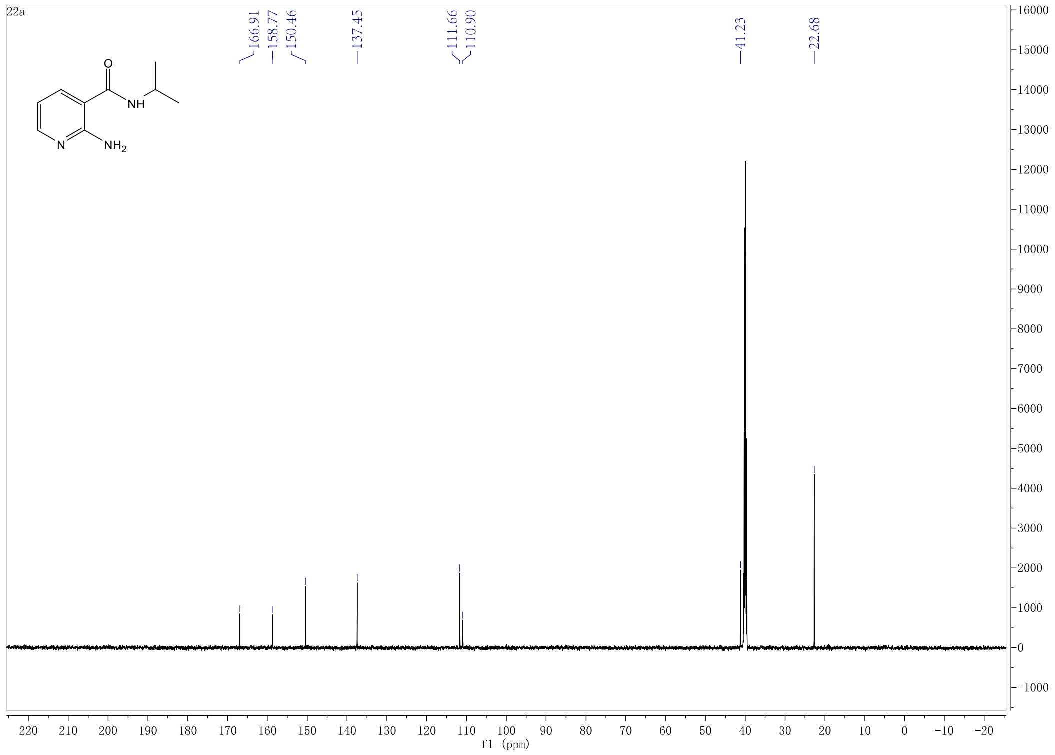

^{13}C NMR spectra of 22a

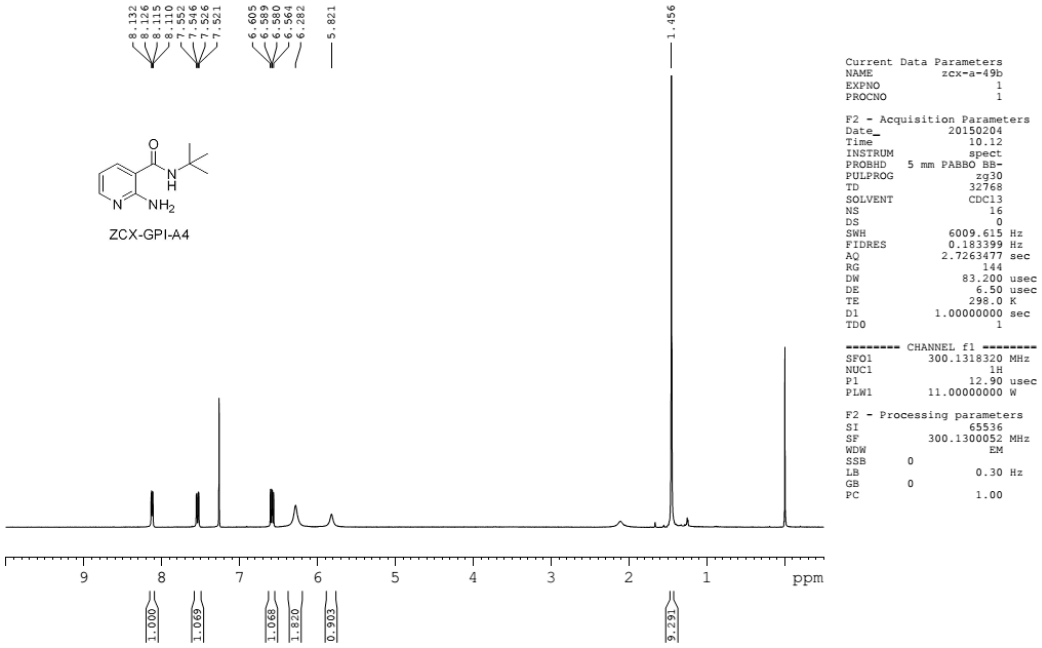

^{1}H NMR spectra of 22b

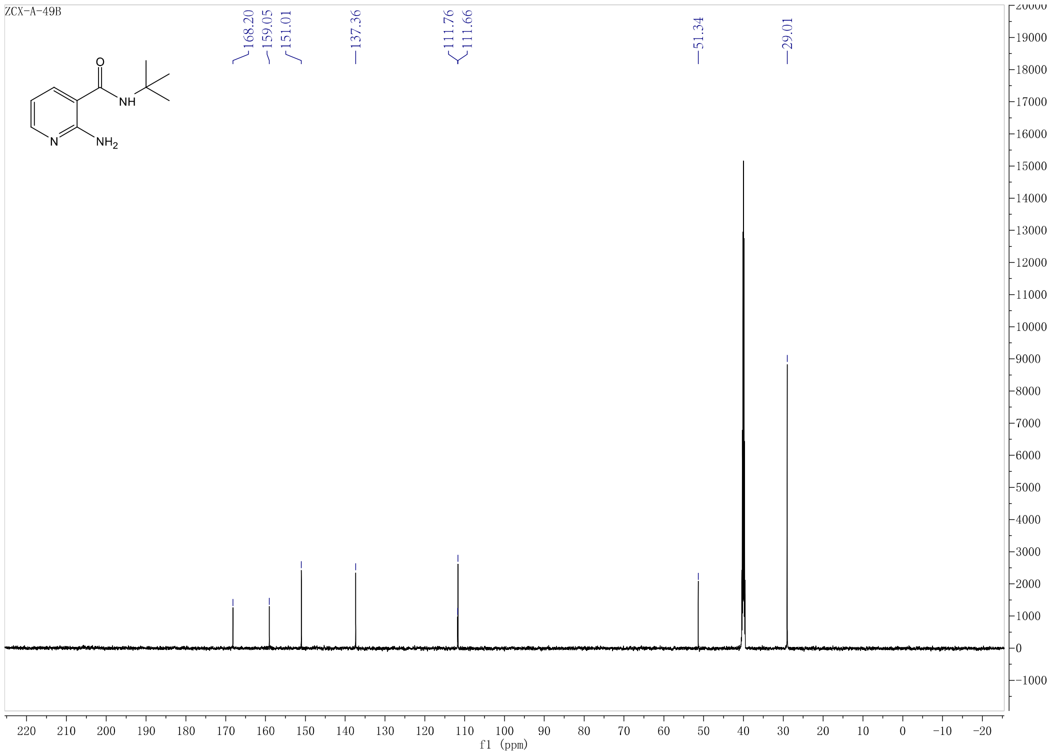

^{13}C NMR spectra of 22b

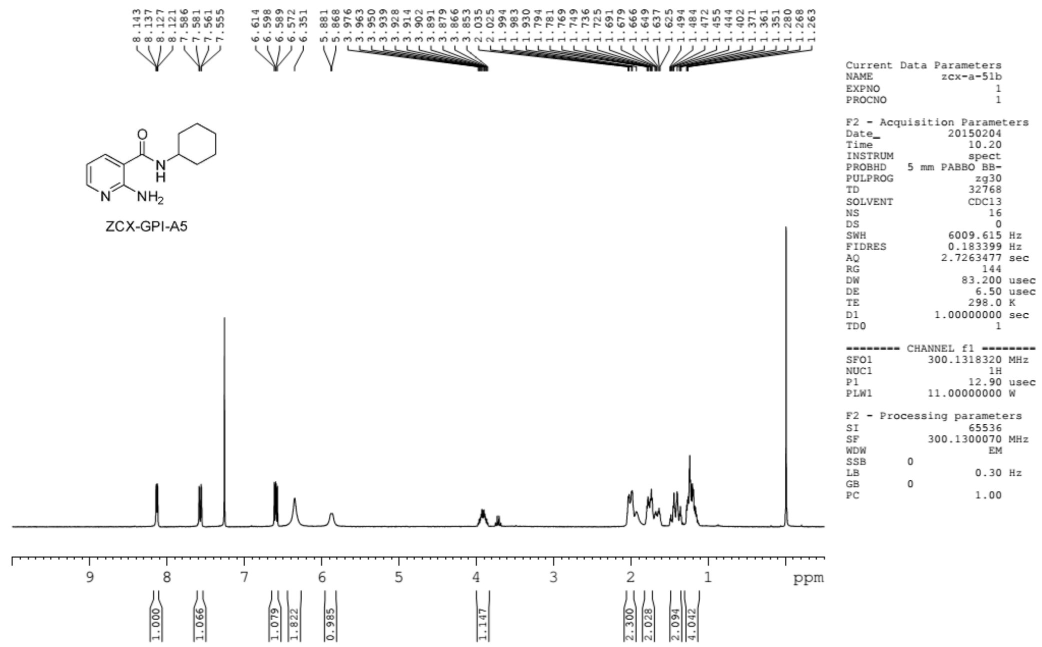

^{1}H NMR spectra of 22c

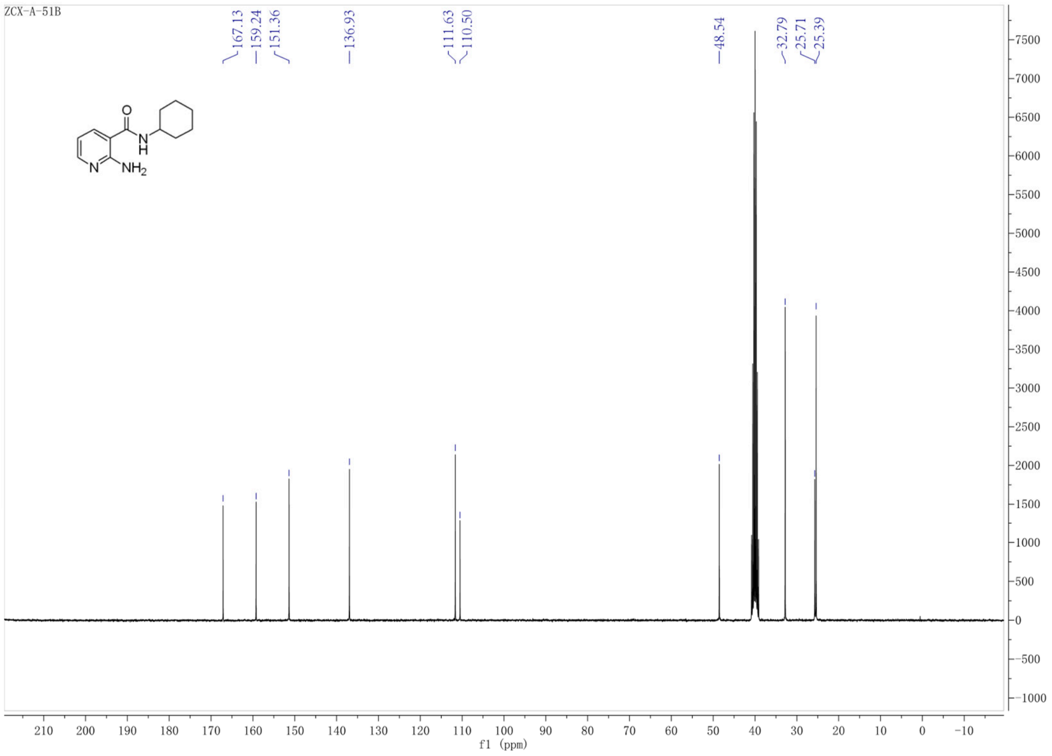

^{13}C NMR spectra of 22c

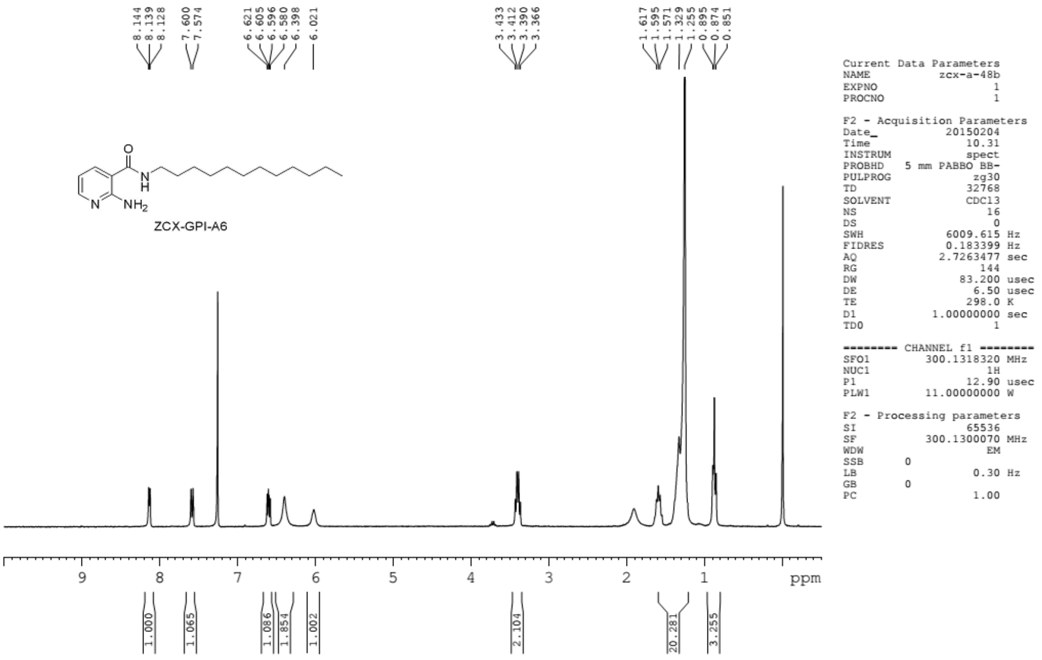

^{1}H NMR spectra of 22d

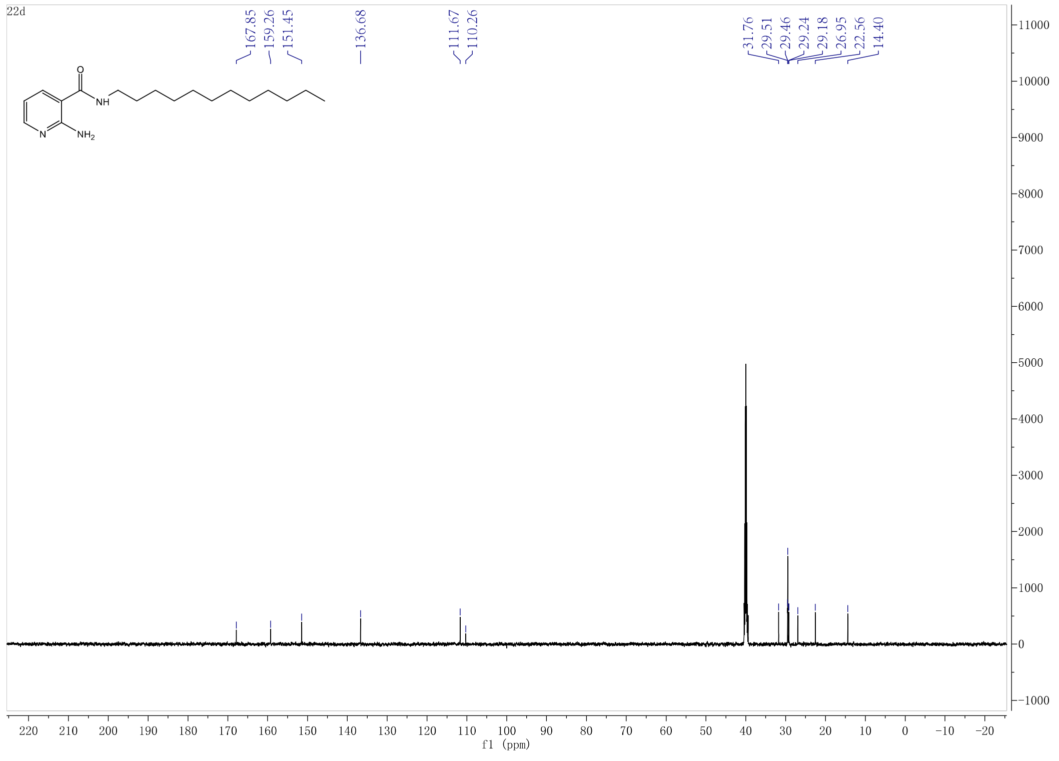

^{13}C NMR spectra of 22d

HPLC Spectra of Representative Compounds

```
Data File C:\CHEM32\1\DATA\ZMZ\TEST 2023-01-11 09-52-18\16G-2U.D
Sample Name: 16g-2u

    =====================================================================
    Acq. Operator   : SYSTEM                          Seq. Line :    9
    Acq. Instrument : 1260lc                           Location : Vial 41
    Injection Date  : 1/11/2023 11:43:21 AM                  Inj :    1
                                                     Inj Volume : 5.000 µl
    Different Inj Volume from Sample Entry!  Actual Inj Volume : 2.000 µl
    Method          : C:\CHEM32\1\DATA\ZMZ\TEST 2023-01-11 09-52-18\TEST NTJH.M (Sequence Method)
    Last changed    : 1/11/2023 9:52:18 AM by SYSTEM
```

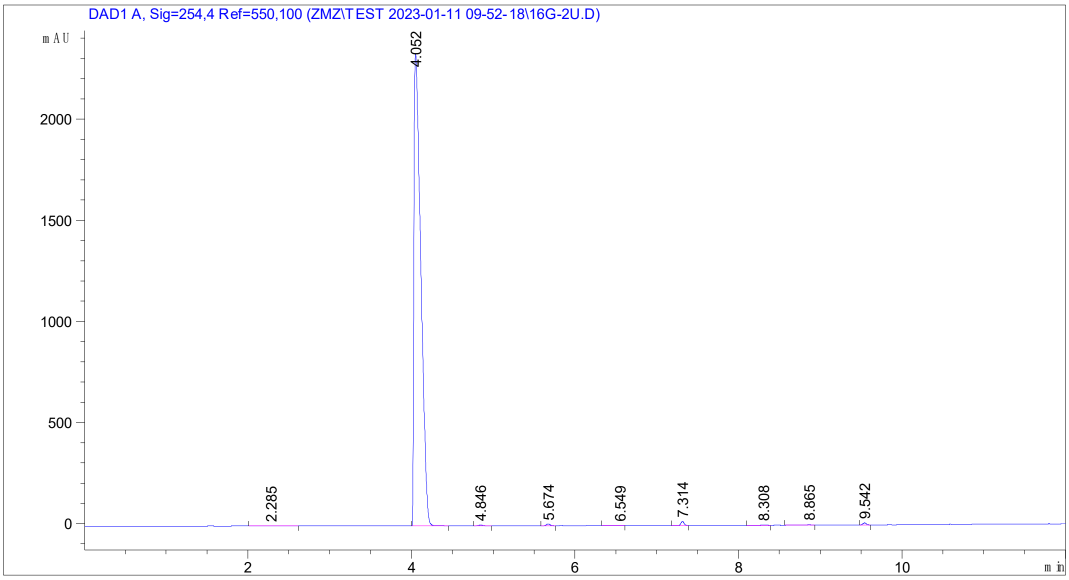


```
    =====================================================================
                             Area Percent Report
    =====================================================================

    Sorted By             :      Signal
    Multiplier            :      1.0000
    Dilution              :      1.0000
    Use Multiplier & Dilution Factor with ISTDs


    Signal 1: DAD1 A, Sig=254,4 Ref=550,100
```

Peak #	RetTime [min]	Type	Width [min]	Area [mAU*s]	Height [mAU]	Area %
1	2.285	BB	0.1964	31.20372	1.89748	0.2260
2	4.052	BB	0.0910	1.36156e4	2334.45874	98.6292
3	4.846	BB	0.0629	16.27699	3.96648	0.1179
4	5.674	BV	0.0497	28.58175	8.85165	0.2070
5	6.549	BV	0.0664	7.33513	1.54507	0.0531
6	7.314	BV	0.0414	55.62617	20.65764	0.4029
7	8.308	BB	0.0623	8.81839	1.98709	0.0639
8	8.865	BB	0.0692	13.28968	2.68589	0.0963
9	9.542	BB	0.0439	28.09934	9.96804	0.2035

HPLC spectra of 16g

Data File C:\CHEM32\1\DATA\ZMZ\TEST 2023-01-11 09-52-18\16J-2U.D
Sample Name: 16j-2u

===

Acq. Operator : SYSTEM Seq. Line : 12
Acq. Instrument : 1260lc Location : Vial 44
Injection Date : 1/11/2023 12:24:45 PM Inj : 1
Inj Volume : 5.000 µl
Different Inj Volume from Sample Entry! Actual Inj Volume : 2.000 µl
Acq. Method : C:\CHEM32\1\DATA\ZMZ\TEST 2023-01-11 09-52-18\TEST NTJH.M
Last changed : 1/11/2023 12:29:49 PM by SYSTEM
(modified after loading)
Analysis Method : C:\CHEM32\1\DATA\ZMZ\TEST 2023-01-11 09-52-18\TEST NTJH.M (Sequence Method)
Last changed : 1/11/2023 9:52:18 AM by SYSTEM

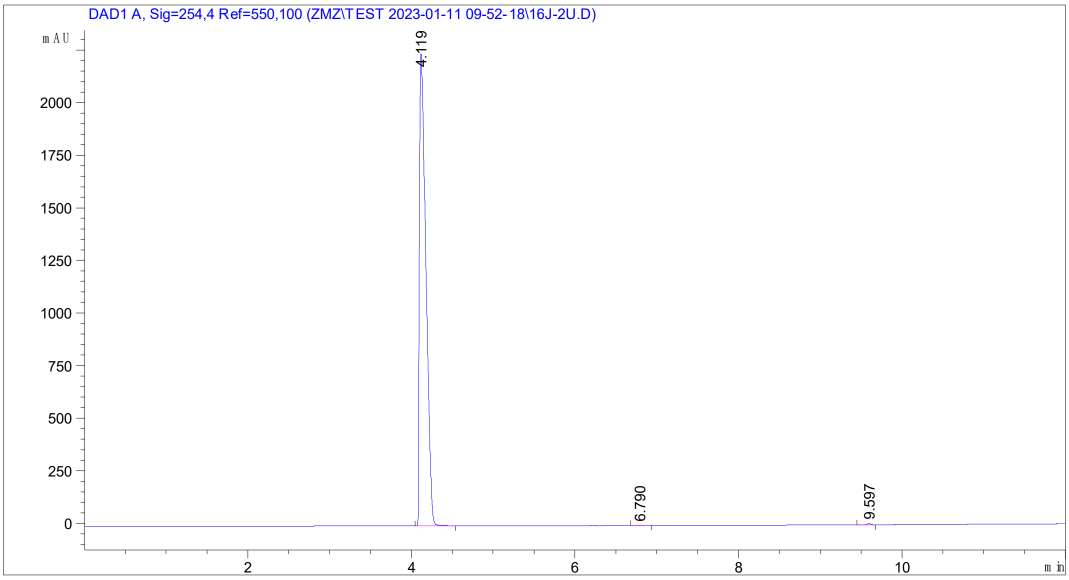

===

Area Percent Report

===

Sorted By : Signal
Multiplier : 1.0000
Dilution : 1.0000
Use Multiplier & Dilution Factor with ISTDs

Signal 1: DAD1 A, Sig=254,4 Ref=550,100

Peak #	RetTime [min]	Type	Width [min]	Area [mAU*s]	Height [mAU]	Area %
1	4.119	BV	0.0841	1.23168e4	2243.02368	99.8032
2	6.790	BB	0.0612	5.75675	1.27470	0.0466
3	9.597	BV	0.0453	18.52760	6.20405	0.1501

Totals : 1.23410e4 2250.50243

HPLC spectra of 16j

Data File C:\CHEM32\1\DATA\ZMZ\TEST 2023-01-11 09-52-18\16P.D
Sample Name: 16p

===

Acq. Operator : SYSTEM Seq. Line : 6
Acq. Instrument : 1260lc Location : Vial 45
Injection Date : 1/11/2023 11:02:04 AM Inj : 1
Inj Volume : 5.000 µl
Different Inj Volume from Sample Entry! Actual Inj Volume : 2.000 µl
Method : C:\CHEM32\1\DATA\ZMZ\TEST 2023-01-11 09-52-18\TEST NTJH.M (Sequence Method)
Last changed : 1/11/2023 9:52:18 AM by SYSTEM

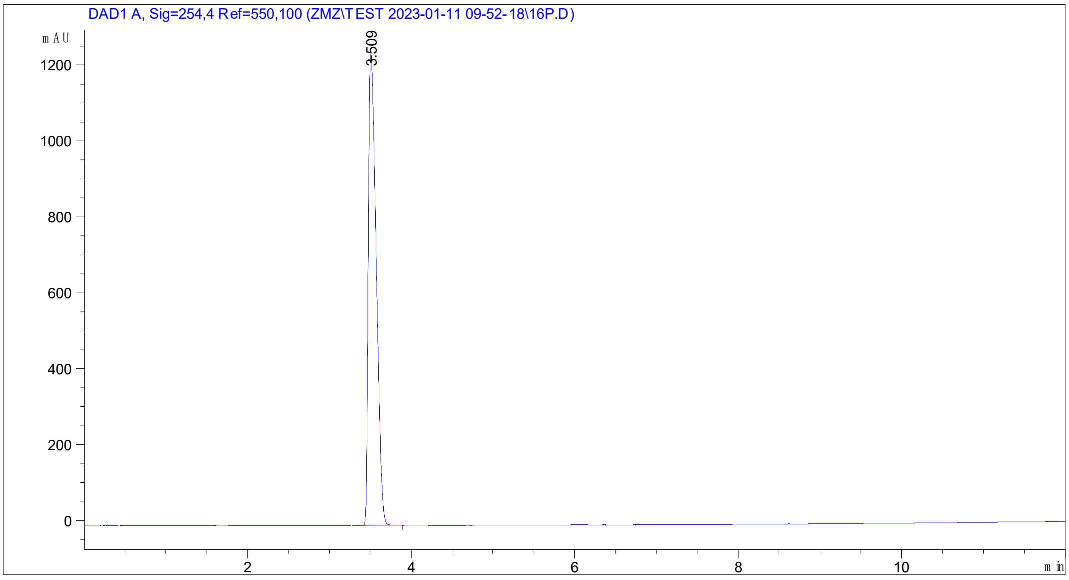

===

Area Percent Report

===

Sorted By : Signal
Multiplier : 1.0000
Dilution : 1.0000
Use Multiplier & Dilution Factor with ISTDs

Signal 1: DAD1 A, Sig=254,4 Ref=550,100

Peak #	RetTime [min]	Type	Width [min]	Area [mAU*s]	Height [mAU]	Area %
1	3.509	BB	0.1074	8352.11426	1243.42444	100.0000

Totals : 8352.11426 1243.42444

===

*** End of Report ***

HPLC spectra of 16p

Data File C:\CHEM32\1\DATA\ZMZ\TEST 2023-01-11 09-52-18\16T-2U.D
Sample Name: 16t-2u

===

Acq. Operator : SYSTEM Seq. Line : 11
Acq. Instrument : 1260lc Location : Vial 43
Injection Date : 1/11/2023 12:11:00 PM Inj : 1
Inj Volume : 5.000 µl
Different Inj Volume from Sample Entry! Actual Inj Volume : 2.000 µl
Method : C:\CHEM32\1\DATA\ZMZ\TEST 2023-01-11 09-52-18\TEST NTJH.M (Sequence Method)
Last changed : 1/11/2023 9:52:18 AM by SYSTEM

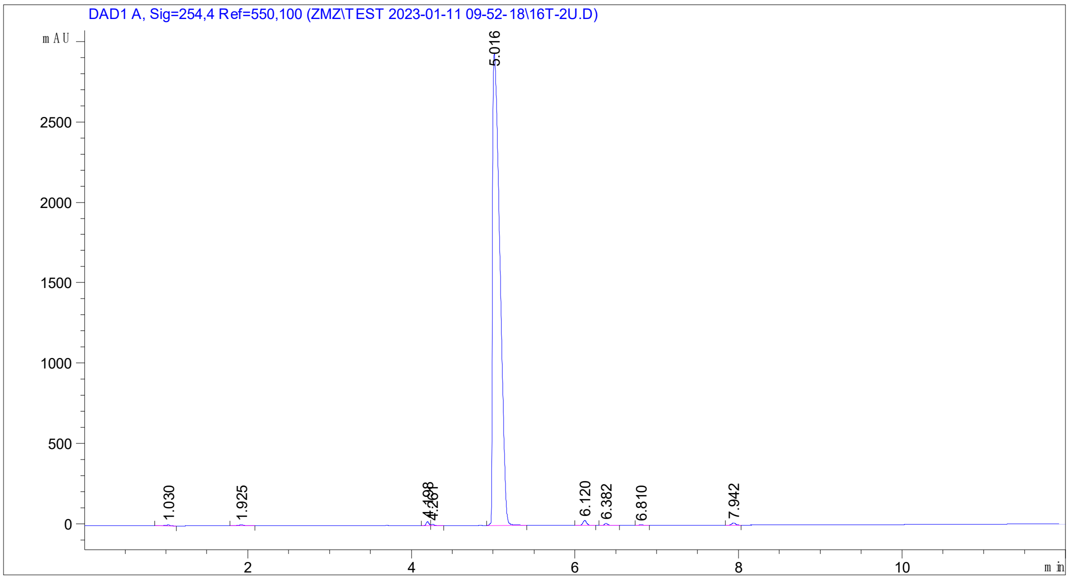

===
Area Percent Report
===

Sorted By : Signal
Multiplier : 1.0000
Dilution : 1.0000
Use Multiplier & Dilution Factor with ISTDs

Signal 1: DAD1 A, Sig=254,4 Ref=550,100

Peak #	RetTime [min]	Type	Width [min]	Area [mAU*s]	Height [mAU]	Area %
1	1.030	BB	0.0534	29.00068	7.28464	0.1583
2	1.925	BB	0.0601	29.14332	6.85114	0.1591
3	4.198	BV	0.0347	55.14526	24.55021	0.3010
4	4.261	VB	0.0359	18.40388	7.68926	0.1005
5	5.016	BV	0.0966	1.80087e4	2935.12622	98.3058
6	6.120	BB	0.0468	91.93481	30.36506	0.5019
7	6.382	BB	0.0442	31.55243	10.92328	0.1722
8	6.810	BB	0.0458	12.31413	4.19324	0.0672
9	7.942	BB	0.0474	42.86133	14.12904	0.2340

HPLC spectra of 16t

Data File C:\CHEM32\1\DATA\ZMZ\TEST 2023-01-11 09-52-18\19.D
Sample Name: 19

===

Acq. Operator : SYSTEM Seq. Line : 7
Acq. Instrument : 1260lc Location : Vial 46
Injection Date : 1/11/2023 11:15:49 AM Inj : 1
Inj Volume : 5.000 µl
Different Inj Volume from Sample Entry! Actual Inj Volume : 2.000 µl
Method : C:\CHEM32\1\DATA\ZMZ\TEST 2023-01-11 09-52-18\TEST NTJH.M (Sequence Method)
Last changed : 1/11/2023 9:52:18 AM by SYSTEM

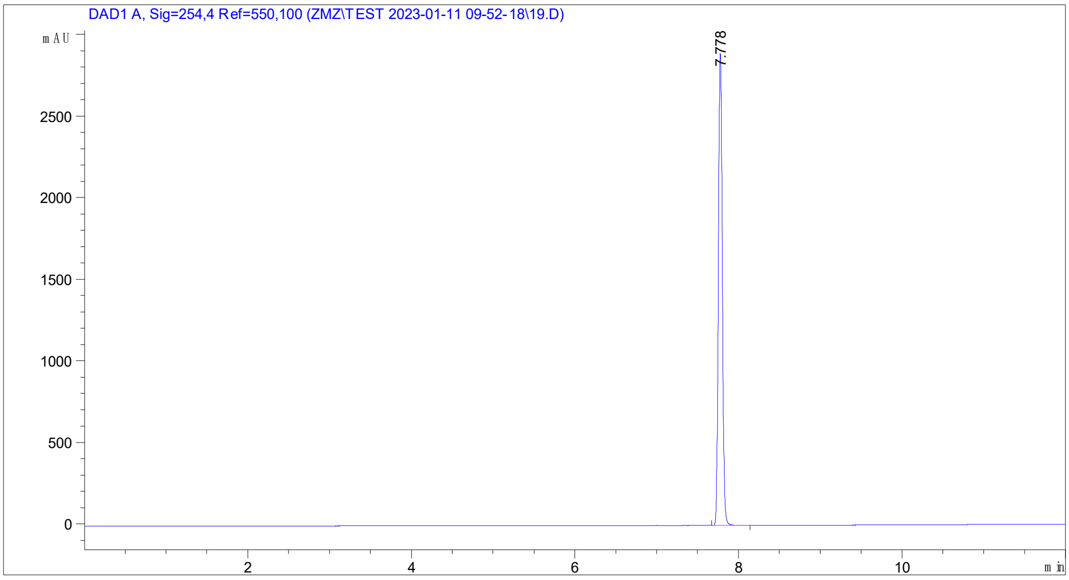

===

Area Percent Report

===

Sorted By : Signal
Multiplier : 1.0000
Dilution : 1.0000
Use Multiplier & Dilution Factor with ISTDs

Signal 1: DAD1 A, Sig=254,4 Ref=550,100

Peak #	RetTime [min]	Type	Width [min]	Area [mAU*s]	Height [mAU]	Area %
1	7.778	BV	0.0524	9641.09277	2891.20068	100.0000
Totals :				9641.09277	2891.20068	

===

*** End of Report ***

HPLC spectra of 19
